# Supplementary material for: ProteinShader: illustrative rendering of macromolecules
Source: BMC Struct Biol. 2009 Mar 30;9:19. doi: 10.1186/1472-6807-9-19 (PMC2672931; doi:10.1186/1472-6807-9-19)
Supplement: Additional file 1 — ProteinShader program without source code. This compressed file contains the complete ProteinShader program including associated libraries, but no source code. A README.txt file gives an overview of the ProteinShader distribution, and the index.html file in the help subdirectory has directions on getting started with the program as well as a set of tutorials. [file 1472-6807-9-19-S1.zip › ProteinShader-beta-0_9_4-binary/help/api/index-all.html]

 
 
 
 
 
 
Index (ProteinShader API)
 


 

 
function windowTitle()
{
    parent.document.title="Index (ProteinShader API)";
}
 
 
 

 

 


 
   
  
 
 
 
   
 
   
          Overview     
        Package   
        Class   
        Use   
          Tree     
          Deprecated     
       Index    
          Help     
   
 
 
  
 
 
 

 
  
 PREV 
 NEXT  
  
    FRAMES     
   NO FRAMES     
  
    All Classes  ');
  }
  //-->
 
 
    All Classes  
 


  
 
 
  
 

 A   B   C   D   E   F   G   H   I   J   K   L   M   N   O   P   Q   R   S   T   U   V   W   X   Y   Z   
    
 A  
 
   AAColorEnum   - Enum in  org.proteinshader.structure.enums  Provides an enumeration of an amino acid color scheme.   AAPortionMode   - Enum in  org.proteinshader.structure.visitor.enums  Provides an enumeration that can be used to control what portion of an
AminoAcid the Visitor will traverse.   AboutBox   - Class in  org.proteinshader.gui.components.menubar  A simple dialog box that displays a title, an image (PNG, GIG, or JPEG), and a 
close button.   AboutBox(Frame, String, String)   - 
Constructor for class org.proteinshader.gui.components.menubar. AboutBox 
 Constructs an AboutBox.
   AboutBox(Frame, String, File)   - 
Constructor for class org.proteinshader.gui.components.menubar. AboutBox 
 Constructs an AboutBox.
   AboutBoxListenerFactory   - Class in  org.proteinshader.gui.listeners.menubar  Creates the event listeners needed by the AboutBox.   AboutBoxListenerFactory(AboutBox)   - 
Constructor for class org.proteinshader.gui.listeners.menubar. AboutBoxListenerFactory 
 Constructs a AboutBoxListenerFactory.
   accept(File)   - 
Method in class org.proteinshader.gui.utils. FileExtensionFilter 
 Returns true if the file is a directory or has a ".png" extension.
   accept(Visitor)   - 
Method in class org.proteinshader.structure. AminoAcid 
 Accepts a Visitor and does a callback.
   accept(Visitor)   - 
Method in class org.proteinshader.structure. Atom 
 Accepts a Visitor and does a callback.
   accept(Visitor)   - 
Method in class org.proteinshader.structure. BetaStrand 
 Accepts a Visitor and does a callback.
   accept(Visitor)   - 
Method in class org.proteinshader.structure. Bond 
 Accepts a Visitor and does a callback.
   accept(Visitor)   - 
Method in class org.proteinshader.structure. Chain 
 Accepts a Visitor and does a callback.
   accept(Visitor)   - 
Method in class org.proteinshader.structure. Drawable 
 Accepts a Visitor and does a callback.
   accept(Visitor)   - 
Method in class org.proteinshader.structure. Helix 
 Accepts a Visitor and does a callback.
   accept(Visitor)   - 
Method in class org.proteinshader.structure. Heterogen 
 Accepts a Visitor and does a callback.
   accept(File)   - 
Method in class org.proteinshader.structure.io.filters. PDBFileFilter 
 Returns true if the file is a directory or has a "pdb" extension.
   accept(Visitor)   - 
Method in class org.proteinshader.structure. Loop 
 Accepts a Visitor and does a callback.
   accept(Visitor)   - 
Method in class org.proteinshader.structure. Model 
 Accepts a Visitor and does a callback.
   accept(Visitor)   - 
Method in class org.proteinshader.structure. Region 
 Accepts a Visitor and does a callback.
   accept(Visitor)   - 
Method in class org.proteinshader.structure. Residue 
 Accepts a Visitor and does a callback.
   accept(Visitor)   - 
Method in class org.proteinshader.structure. Segment 
 Accepts a Visitor and does a callback.
   accept(Visitor)   - 
Method in class org.proteinshader.structure. Structure 
 Accepts a Visitor and does a callback.
   accept(Visitor)   - 
Method in interface org.proteinshader.structure.visitor. Visitable 
 Accepts a Vistor so that a call back can be done.
   accept(Visitor)   - 
Method in class org.proteinshader.structure. Water 
 Accepts a Visitor and does a callback.
   add(SpringLoadedJFrame)   - 
Method in interface org.proteinshader.gui. Mediator 
 Adds the address of the control panel frame to the Mediator.
   add(ControlPanel)   - 
Method in interface org.proteinshader.gui. Mediator 
 Adds the address of the ControlPanel to the Mediator.
   add(MainMenuBar)   - 
Method in interface org.proteinshader.gui. Mediator 
 Adds the address of the MainMenuBar to the Mediator.
   add(Renderer)   - 
Method in interface org.proteinshader.gui. Mediator 
 Adds the address of the Renderer to the Mediator.
   add(SpringLoadedJFrame)   - 
Method in class org.proteinshader.gui. MediatorImpl 
 Adds the address of the ControlFrame to the Mediator.
   add(ControlPanel)   - 
Method in class org.proteinshader.gui. MediatorImpl 
 Adds the address of the ControlPanel to the Mediator.
   add(MainMenuBar)   - 
Method in class org.proteinshader.gui. MediatorImpl 
 Adds the address of the MainMenuBar to the Mediator.
   add(Renderer)   - 
Method in class org.proteinshader.gui. MediatorImpl 
 Adds the address of the Renderer to the Mediator.
   add(Vec3d)   - 
Method in class org.proteinshader.math. Point3d 
 Returns the point created by adding the vector given as an
    argument to the point that is the calling object.
   add(Vec3d)   - 
Method in class org.proteinshader.math. Vec3d 
 Creates a new vector by adding the vector given as an argument to
    the calling vector (the existing vectors are NOT modified).
   add(AtomModifier)   - 
Method in class org.proteinshader.structure.visitor. ModifierVisitor 
 Adds a AtomModifier.
   add(BondModifier)   - 
Method in class org.proteinshader.structure.visitor. ModifierVisitor 
 Adds a BondModifier.
   add(SegmentModifier)   - 
Method in class org.proteinshader.structure.visitor. ModifierVisitor 
 Adds a SegmentModifier.
   addAllOtherComponentsToCenterPanel()   - 
Method in class org.proteinshader.gui.components.controlpanel. OptimizePanel 
 This helper method for the constructor adds the radio buttons and
    the tiling spinner to the center panel.
   addAntialiasHalftoningPanel()   - 
Method in class org.proteinshader.gui.components.controlpanel. AntialiasingPanel 
 This helper method for the constructor adds the radio buttons and
    the tiling spinner to the center panel.
   addAntialiasScenePanel()   - 
Method in class org.proteinshader.gui.components.controlpanel. AntialiasingPanel 
 This helper method for the constructor adds a check box for turning whole
    scene antialiasing on and a combo box for setting the number of times to
    jitter the scene.
   addAtomsToAtomList(Residue)   - 
Method in class org.proteinshader.gui.components.controlpanel. SelectorPanel 
 Adds all Atoms in the Residue to the Atom list.
   addAutoTilingButtonPanelToNorth()   - 
Method in class org.proteinshader.gui.components.controlpanel. OptimizePanel 
 This helper method for the constructor adds a check box button
    for turning automatic tiling (level of detail) on/off.
   addGlyph(Glyph)   - 
Method in class org.proteinshader.graphics.typography. GlyphSet 
 Adds a Glyph object to this GlyphSet.
   addLine(String)   - 
Method in class org.proteinshader.structure. Record 
 Adds a line to the Record.
   addLoop(Loop)   - 
Method in class org.proteinshader.structure. Model 
 Adds a Loop to the Model.
   addNewAminoAcid(AminoAcidEnum, String)   - 
Method in class org.proteinshader.structure. Chain 
 Creates a new AminoAcid with the type and Residue ID given as
    arguments, adds the AminoAcid to the Chain's collection of
    AminoAcids, and returns a reference to the new AminoAcid.
   addNewAtom(int, AtomEnum, String, double, int, double, String, double, double, double, VisibilityEnum)   - 
Method in class org.proteinshader.structure. AminoAcid 
 Creates a new Atom with the type and Atom ID given as arguments,
    adds the Atom to the Residue's collection of Atoms, and
    returns a reference to the new Atom.
   addNewAtom(int, AtomEnum, String, double, int, double, String, double, double, double, VisibilityEnum)   - 
Method in class org.proteinshader.structure. Residue 
 Creates a new Atom with the type and Atom ID given as arguments,
    adds the Atom to the Residue's collection of Atoms, and
    returns a reference to the new Atom.
   addNewAtom(int, AtomEnum, String, String, String, String, String, double, int, double, String, double, double, double, VisibilityEnum)   - 
Method in class org.proteinshader.structure. Structure 
 Creates a new atom with the atomID given as an argument, adds the
    new Atom to the requested Model-Chain-AminoAcid, and then returns
    a reference to the new Atom.
   addNewBetaStrand(String, String, String, String, String, int, int)   - 
Method in class org.proteinshader.structure. Chain 
 Creates a new BetaStrand with the BetaStrand ID given as an
    argument, adds the BetaStrand to the Chain's collection of
    BetaStrands, and returns a reference to the new BetaStrand.
   addNewBetaStrand(String, String, String, String, String, String, int, int)   - 
Method in class org.proteinshader.structure. Model 
 The BetaStrand will be added to the Chain only if the sequence of
    Residues that the BetaStrand refers to has already been added.
   addNewBetaStrand(String, String, String, String, String, String, int, int)   - 
Method in class org.proteinshader.structure. Structure 
 The BetaStrand will be added to the equivalent Chain on each
    Model, but this addition will only work if the sequence of
    Residues that the BetaStrand refers to have already been added.
   addNewBond(Atom, BondEnum, VisibilityEnum)   - 
Method in class org.proteinshader.structure. Atom 
 Adds a new Bond with this Atom as the source Atom and the Atom
    given as an argument as the destination Atom.
   addNewCategory(String)   - 
Method in class org.proteinshader.structure. Description 
 Creates a Category, adds it to a hash, and then returns the new
    Category.
   addNewChain(String)   - 
Method in class org.proteinshader.structure. Model 
 Creates a new Chain with the chainID given as an argument, adds
    the Chain to the Model's collection of Chains, and returns a
    reference to the new Chain.
   addNewHelix(String, String, String, String, HelixEnum)   - 
Method in class org.proteinshader.structure. Chain 
 Creates a new Helix with the Helix ID given as an argument, adds
    the Helix to the Chain's collection of Helices, and returns a
    reference to the new Helix.
   addNewHelix(String, String, String, String, String, HelixEnum)   - 
Method in class org.proteinshader.structure. Model 
 The Helix will be added to the Chain only if the sequence of
    Residues that the Helix refers to has already been added.
   addNewHelix(String, String, String, String, String, HelixEnum)   - 
Method in class org.proteinshader.structure. Structure 
 The Helix will be added to the equivalent Chain on each Model, but
    this addition will only work if the sequence of Residues that the
    Helix refers to have already been added.
   addNewHetAtom(int, AtomEnum, String, String, String, String, String, double, int, double, String, double, double, double, VisibilityEnum)   - 
Method in class org.proteinshader.structure. Structure 
 Creates a new atom with the atomID given as an argument, adds the
    new Atom to the requested Model-Chain-(Heterogen or Water), and
    then returns a reference to the new Atom.
   addNewHeterogen(String, String)   - 
Method in class org.proteinshader.structure. Chain 
 Creates a new Heterogen with the name and Residue ID given as
    arguments, adds the Heterogen to the Chain's collection of
    Heterogens, and returns a reference to the new Heterogen.
   addNewLine(String, String, String)   - 
Method in class org.proteinshader.structure. Description 
 Adds a line to the requested Category/Record.
   addNewLoop(String, String, String)   - 
Method in class org.proteinshader.structure. Chain 
 Creates a new Loop with the Loop ID given as an argument, adds the
    Loop to the Chain's collection of Loops, and returns a reference
    to the new Loop.
   addNewModel(String)   - 
Method in class org.proteinshader.structure. Structure 
 Creates a Model, adds it to the Structure, and returns a reference
    to the new Model.
   addNewRecord(String)   - 
Method in class org.proteinshader.structure. Category 
 Creates a Record, adds it to a hash, and returns the new Record.
   addNewStructure(Structure)   - 
Method in class org.proteinshader.gui.components.controlpanel. ControlPanel 
 The Structure is used to set the Model menu of the SelectorPanel,
    which will automatically set the other menus and lists of the
    SelectorPanel.
   addNewWater(String)   - 
Method in class org.proteinshader.structure. Chain 
 Creates a new Water with the Residue ID given as an argument,
    adds the Water to the Chain's collection of Waters, and returns
    a reference to the new Water.
   addToMe(Vec3d)   - 
Method in class org.proteinshader.math. Point3d 
 Tranlates the calling Point3d by adding the vector xyz-coordinates
    to it.
   addToMe(Vec3d)   - 
Method in class org.proteinshader.math. Vec3d 
 Modifies the calling vector by adding the xyz-values of the vector
    given as an argument to the xyz-values of the calling vector.
   adjustMyTangent(Vec3d)   - 
Method in class org.proteinshader.math. Quaternion 
 Modifies this quaternion by rotating it such that if it is
    converted into a rotation matrix [N B T], the T vector will
    match the tangent given as an argument.
   adjustTangent(Vec3d)   - 
Method in class org.proteinshader.math. Quaternion 
 Returns a copy of this quaternion that has been rotated such that
    if it is converted into a rotation matrix [N B T], the T vector
    will match the tangent given as an argument.
   aim(GL)   - 
Method in class org.proteinshader.gui.viewing. Camera 
 Uses gluLookAt() to position and aim the camera.
   ALPHA_HEIGHT   - 
Static variable in class org.proteinshader.graphics. Ribbon 
 The default ribbon height (y-axis dimension) for
        an alpha-helix region is 3.0 angstroms.
   ALPHA_RADIUS   - 
Static variable in class org.proteinshader.graphics. Tube 
 The default radius for a Helix region waist polygon
         is 1.5 angstroms.
   ALPHA_RADIUS   - 
Static variable in class org.proteinshader.structure.io. TubeVertices 
 The default radius for a Helix region waist polygon
         is 1.5 angstroms.
   ALPHA_S_COORD_END   - 
Static variable in class org.proteinshader.graphics. Ribbon 
 The texture s-coordinate for alpha-helices ends at 1.0.
   ALPHA_S_COORD_START   - 
Static variable in class org.proteinshader.graphics. Ribbon 
 The texture s-coordinate for alpha-helices starts at 0.0.
   ALPHA_SLICES   - 
Static variable in class org.proteinshader.graphics. Tube 
 The default number of slices for a Helix region waist polygon
        is 20.
   ALPHA_SLICES   - 
Static variable in class org.proteinshader.structure.io. TubeVertices 
 The default number of slices for a Helix region waist polygon
        is 16.
   ALPHA_STACKS   - 
Static variable in class org.proteinshader.graphics. Ribbon 
 The default number of stacks for
        an alpha-helix segment is 10.
   ALPHA_STACKS   - 
Static variable in class org.proteinshader.graphics. Tube 
 The default number of stacks for
        an alpha-helix segment is 10.
   ALPHA_STACKS   - 
Static variable in class org.proteinshader.structure.io. TubeVertices 
 The default number of stacks for
        an alpha-helix segment is 10.
   ALPHA_THIN_SIDE_S_MAX   - 
Static variable in class org.proteinshader.graphics. Ribbon 
 The texture s-coordinate for the thin side of alpha-helices
        ends at 0.125.
   ALPHA_THIN_SIDE_S_MIN   - 
Static variable in class org.proteinshader.graphics. Ribbon 
 The texture s-coordinate for the thin side of alpha-helices
        starts at 0.0.
   ALPHA_WIDTH   - 
Static variable in class org.proteinshader.graphics. Ribbon 
 The default ribbon width (x-axis dimension) for an alpha-helix
        region is 0.2 angstroms.
   AMBIENT_LIGHT   - 
Static variable in class org.proteinshader.gui.viewing. Lighting 
 The ambient light is { 0.2f, 0.2f, 0.2f, 0.0f }.
   AminoAcid   - Class in  org.proteinshader.structure  This concrete subclass of the abstract class Residue stores
information on an amino acid in a PDB structure entry.   AminoAcid(AminoAcidEnum, String, String, String, String)   - 
Constructor for class org.proteinshader.structure. AminoAcid 
 Constructs an AminoAcid with the type and IDs given as arguments.
   AminoAcidEnum   - Enum in  org.proteinshader.structure.enums  Provides an enumeration of the amino acids found in protein.   AminoAcidLabelVisitor   - Class in  org.proteinshader.structure.visitor  Traverses the AminoAcids of a Structure and builds a non-redundant
list of residueIDs.   AminoAcidLabelVisitor()   - 
Constructor for class org.proteinshader.structure.visitor. AminoAcidLabelVisitor 
 Constructs an AminoAcidLabelVisitor.
   angleBetweenNormals(Quaternion)   - 
Method in class org.proteinshader.math. Quaternion 
 Returns the angle between the normals of the calling and argument
    quaternions, where the normal is the first column vector of the
    rotation matrix [N B T] that is the equivalent of a quaternion.
   AntialiasingPanel   - Class in  org.proteinshader.gui.components.controlpanel  This control panel allows the user to turn antialiasing (edge smoothing)
effects on or off.   AntialiasingPanel(Mediator, Frame)   - 
Constructor for class org.proteinshader.gui.components.controlpanel. AntialiasingPanel 
 Constructs an AntialiasingPanel.
   AntialiasingPanelListenerFactory   - Class in  org.proteinshader.gui.listeners.controlpanel  Creates the listeners for the AntialiasingPanel.   AntialiasingPanelListenerFactory(Mediator, AntialiasingPanel)   - 
Constructor for class org.proteinshader.gui.listeners.controlpanel. AntialiasingPanelListenerFactory 
 Constructs a AntialiasingPanelListenerFactory.
   antialiasScene(boolean)   - 
Method in class org.proteinshader.gui.components.controlpanel. AntialiasingPanel 
 Determines if the entire scene should be antialiased (smoothed out) by
    jittering the scene multiple times to the OpenGL accumulation buffer and
    blending the images.
   antialiasScene(JitterEnum)   - 
Method in class org.proteinshader.gui.components.controlpanel. AntialiasingPanel 
 The jitter value given as an argument is only applied to the scene if
    the 'Antialias entire scene' checkbox is currently selected.
   applyBendTexture()   - 
Method in class org.proteinshader.gui.components.controlpanel. DecorationsPanel 
 Applies the current "Bend Texture:" selection to whatever Segments
    are selected in the RadioPanel.
   applyColors(float, float, float)   - 
Method in class org.proteinshader.gui.components.controlpanel. ColorPanel 
 Applies the RGB color to whatever item or items are currently
    selected in the RadioPanel.
   applyDefaultColors1()   - 
Method in class org.proteinshader.gui.components.controlpanel. ColorPanel 
 Applies the default RGB color to whatever item or items are
    currently selected in the RadioPanel.
   applyDefaultColors2()   - 
Method in class org.proteinshader.gui.components.controlpanel. ColorPanel 
 Applies the default RGB color to whatever item or items are
    currently selected in the RadioPanel.
   applyDefaultScale()   - 
Method in class org.proteinshader.gui.components.controlpanel. AtomScalePanel 
 Applies the default scale to the Atoms (spheres) of whatever item
    or items are currently selected in the RadioPanel, but only if the
    current style is SPACE_FILLING or BALLS_AND_STICKS.
   applyDefaultTilingNumber()   - 
Method in class org.proteinshader.gui.components.controlpanel. OptimizePanel 
 Applies the default number tiling number (slices and/or stacks)
    for the currently selected geometric object.
   applyEndCapColor(GL, float, float)   - 
Method in class org.proteinshader.graphics. ExtrudedShape 
 Calls the setMaterial() method of superclass Shape with the
    END_CAP_COLOR along with the alpha value and specular exponent
    given as aguments.
   applyHalftoningAndBendTextures()   - 
Method in class org.proteinshader.gui.components.controlpanel. DecorationsPanel 
 Applies the current "Patterns" texture selection to whatever
    Segments are selected in the RadioPanel.
   applyHalftoningTexture()   - 
Method in class org.proteinshader.gui.components.controlpanel. DecorationsPanel 
 Applies the current "Patterns" texture selection to whatever
    Segments are selected in the RadioPanel.
   applyPatternsTexture()   - 
Method in class org.proteinshader.gui.components.controlpanel. DecorationsPanel 
 Applies the current "Patterns" texture selection to whatever
    Segments are selected in the RadioPanel.
   applyScale(double)   - 
Method in class org.proteinshader.gui.components.controlpanel. AtomScalePanel 
 Applies the scale to the Atoms (spheres) of whatever item or items
    are currently selected in the RadioPanel, but only if the style
    is SPACE_FILLING or BALLS_AND_STICKS.
   applySpinnerTilingNumber()   - 
Method in class org.proteinshader.gui.components.controlpanel. OptimizePanel 
 Applies the tiling number in the spinner to the currently selected
    geometric object.
   applyStartCapColor(GL, float, float)   - 
Method in class org.proteinshader.graphics. ExtrudedShape 
 Calls the setMaterial() method of superclass Shape with the
    START_CAP_COLOR along with the alpha value and specular exponent
    given as aguments.
   applyThinSidesColor(GL, float, float)   - 
Method in class org.proteinshader.graphics. Ribbon 
 Calls the setMaterial() method of superclass Shape with the
    THIN_SIDES_COLOR along with the alpha value and specular exponent
    given as aguments.
   applyVisibility(VisibilityEnum, float)   - 
Method in class org.proteinshader.gui.components.controlpanel. VisibilityPanel 
 Applies the requested visiblity status and alpha value to whatever
    item or items are currently selected in the RadioPanel.
   ascendingMergeSort(Drawable[])   - 
Method in class org.proteinshader.structure.sort. DrawableSorter 
 Sorts an array of Drawable objects in ascending order based on
    camera distance.
   Atom   - Class in  org.proteinshader.structure  Extends abstract class Drawable to add data that is unique to an atom
(atomID, type, temperature, occupancy, radius,  etc. ).   Atom(int, AtomEnum, String, String, String, String, String, double, int, double, String, double, double, double, VisibilityEnum)   - 
Constructor for class org.proteinshader.structure. Atom 
 This constructor uses the type argument (an AtomEnum) to set the
    default radius and CPK color for the Atom.
   ATOM_VISIBILITY   - 
Static variable in class org.proteinshader.structure.io. PDBLineParser 
 The default visibilty for Atoms.
   AtomColorPanel   - Class in  org.proteinshader.gui.components.controlpanel  This control panel allows the user to modify the color of Atoms.   AtomColorPanel(Mediator, Frame, RadioPanel)   - 
Constructor for class org.proteinshader.gui.components.controlpanel. AtomColorPanel 
 Constructs a ColorPanel.
   AtomEnum   - Enum in  org.proteinshader.structure.enums  Provides an enumeration of atom types: C, H, N, O, S,  etc. 

  
Includes the periodic table of the elements from hydrogen (atomic
number 1) to roentgenium (atomic number 111).   AtomModifier   - Class in  org.proteinshader.structure.visitor.modifiers  This helper class for the ModifierVisitor knows how to accept an Atom
object and modify its attributes.   AtomModifier()   - 
Constructor for class org.proteinshader.structure.visitor.modifiers. AtomModifier 
 Constructs an AtomModifier.
   AtomScalePanel   - Class in  org.proteinshader.gui.components.controlpanel  This control panel allows the user to modify the scale for the radii
of Atoms in Space-Filling or Balls-and-Sticks style displays.   AtomScalePanel(Mediator, Frame, RadioPanel)   - 
Constructor for class org.proteinshader.gui.components.controlpanel. AtomScalePanel 
 Constructs a AtomScalePanel.
   AtomVisibilityPanel   - Class in  org.proteinshader.gui.components.controlpanel  This control panel allows the user to modify the visibility status and
alpha value of Atoms.   AtomVisibilityPanel(Mediator, Frame, RadioPanel)   - 
Constructor for class org.proteinshader.gui.components.controlpanel. AtomVisibilityPanel 
 Constructs a VisibilityPanel.
   AUTO_TILING   - 
Static variable in class org.proteinshader.graphics.adapter. StructureToGraphics 
 Automatic tiling (level of detail) for spheres and
        cylinders is set to true by default.
   AXIS_RADIUS   - 
Static variable in class org.proteinshader.graphics. FrenetFrames 
 The radius for an axis is 0.05 angtroms.
 
 
    
 B  
 
   B1_MASK   - 
Static variable in class org.proteinshader.gui.listeners. CanvasMouseListener 
 Abbreviated version of mouse button 1 mask for class InputEvent.
   B2_MASK   - 
Static variable in class org.proteinshader.gui.listeners. CanvasMouseListener 
 Abbreviated version of mouse button 2 mask for class InputEvent.
   B3_MASK   - 
Static variable in class org.proteinshader.gui.listeners. CanvasMouseListener 
 Abbreviated version of mouse button 3 mask for class InputEvent.
   BACKGROUND   - 
Static variable in class org.proteinshader.gui.components.controlpanel. ControlPanel 
 The background color for the control panel is light cyan
        (0.9, 1.0, 1.0).
   BACKGROUND   - 
Static variable in class org.proteinshader.gui. Renderer 
 The background color for the canvas is white (1, 1, 1, 0).
   BackgroundMenu   - Class in  org.proteinshader.gui.components.menubar  Provides the 'Background' menu of the MainMenuBar, which allows the
user to change the background color of the canvas.   BackgroundMenu(Mediator, Frame)   - 
Constructor for class org.proteinshader.gui.components.menubar. BackgroundMenu 
 Constructs a BackgroundMenu.
   BackgroundMenuListenerFactory   - Class in  org.proteinshader.gui.listeners.menubar  Creates the event listeners needed by the BackgoundMenu of the MainMenuBar.   BackgroundMenuListenerFactory(Mediator, BackgroundMenu)   - 
Constructor for class org.proteinshader.gui.listeners.menubar. BackgroundMenuListenerFactory 
 Constructs a BackgroundMenuListenerFactory.
   BALL_SCALE   - 
Static variable in enum org.proteinshader.structure.enums. AtomEnum 
 The ball radius (for stick-and-ball models) is calculated by
        multiplying the covalent radius by the BALL_SCALE, which is
        currently set at 0.2.
   BANNER_WIDTH   - 
Static variable in class org.proteinshader.math. LocalFrameDemo 
 The banner width is the number of asterisks
        to print on the line line above or below a
        banner title.
   BANNER_WIDTH   - 
Static variable in class org.proteinshader.math. QuaternionDemo 
 The banner width is the number of asterisks to
        print on the line line above or below a banner
        title.
   BANNER_WIDTH   - 
Static variable in class org.proteinshader.math. SlerpDemo 
 The banner width is the number of asterisks to
        print on the line line above or below a banner
        title.
   BASE   - 
Static variable in class org.proteinshader.graphics. FrenetFrames 
 The small sphere at the base of the xyz-axes is gray.
   BEND_CONFIG   - 
Static variable in class org.proteinshader.graphics.textures. TextureFactory 
 Stores the name of the halftoning configuration file,
        which is currently set to 'halftoning_textures.conf'.
   BEND_DIRECTORY   - 
Static variable in class org.proteinshader.graphics.textures. TextureFactory 
 Stores the relative pathname of the halftoning textures
        directory.
   BETA_HEIGHT   - 
Static variable in class org.proteinshader.graphics. Ribbon 
 The default is 4.0 angstroms.
   BETA_RADIUS   - 
Static variable in class org.proteinshader.graphics. Tube 
 The default radius for a BetaStrand region waist polygon
         is 1.5 angstroms.
   BETA_RADIUS   - 
Static variable in class org.proteinshader.structure.io. TubeVertices 
 The default radius for a BetaStrand region waist polygon
         is 1.5 angstroms.
   BETA_S_COORD_END   - 
Static variable in class org.proteinshader.graphics. Ribbon 
 The texture s-coordinate for beta-strands ends at 1.0.
   BETA_S_COORD_START   - 
Static variable in class org.proteinshader.graphics. Ribbon 
 The texture s-coordinate for beta-strands starts at 0.0.
   BETA_SLICES   - 
Static variable in class org.proteinshader.graphics. Tube 
 The default number of slices for a BetaStrand region waist
        polygon is 20.
   BETA_SLICES   - 
Static variable in class org.proteinshader.structure.io. TubeVertices 
 The default number of slices for a BetaStrand region waist
        polygon is 16.
   BETA_STACKS   - 
Static variable in class org.proteinshader.graphics. Ribbon 
 The default number of stacks for
        a beta-strand segment is 10.
   BETA_STACKS   - 
Static variable in class org.proteinshader.graphics. Tube 
 The default number of stacks for
        a beta-strand segment is 10.
   BETA_STACKS   - 
Static variable in class org.proteinshader.structure.io. TubeVertices 
 The default number of stacks for
        a beta-strand segment is 10.
   BETA_THIN_SIDE_S_MAX   - 
Static variable in class org.proteinshader.graphics. Ribbon 
 The texture s-coordinate for the thin side of beta-strands
        ends at 0.125.
   BETA_THIN_SIDE_S_MIN   - 
Static variable in class org.proteinshader.graphics. Ribbon 
 The texture s-coordinate for the thin side of beta-strands
        starts at 0.0.
   BETA_WIDTH   - 
Static variable in class org.proteinshader.graphics. Ribbon 
 The default ribbon width (x-axis dimension) for a BetaStrand
        region is 0.2 angstroms.
   BetaStrand   - Class in  org.proteinshader.structure  This concrete subclass of Region holds information specific to a
protein BetaStrand.   BetaStrand(String, String, String, String, String, Chain, int, int)   - 
Constructor for class org.proteinshader.structure. BetaStrand 
 Constructs a BetaStrand.
   BetaStrandRecord   - Class in  org.proteinshader.structure.io  This class is used by the PDBLineParser to temporarily store SHEET
records.   BetaStrandRecord(String, String, String, String, String, int, int)   - 
Constructor for class org.proteinshader.structure.io. BetaStrandRecord 
 Constructs a BetaStrandRecord.
   BetaStrandSegmentFactory   - Class in  org.proteinshader.structure.factory  Creates Segment objects for a BetaStrandSegmentFactory.   BetaStrandSegmentFactory()   - 
Constructor for class org.proteinshader.structure.factory. BetaStrandSegmentFactory 
 Constructs a BetaStrandSegmentFactory.
   blendObjectEdges(boolean)   - 
Method in class org.proteinshader.graphics.adapter. StructureToGraphics 
 Turns on or off the ability of the halftoning shaders to soften aliasing
    (jagged edges) by rendering translucent black silhouettes of tube and 
    ribbon segments (and using jitter) before calling on the halftoning shaders.
   blendObjectEdges(boolean)   - 
Method in interface org.proteinshader.gui. Mediator 
 Turns on or off the ability of the halftoning shaders to soften aliasing
    (jagged edges) by rendering translucent black silhouettes of tube and 
    ribbon segments (and using jitter) before calling on the halftoning shaders.
   blendObjectEdges(boolean)   - 
Method in class org.proteinshader.gui. MediatorImpl 
 Turns on or off the ability of the halftoning shaders to soften aliasing
    (jagged edges) by rendering translucent black silhouettes of tube and 
    ribbon segments (and using jitter) before calling on the halftoning shaders.
   blendObjectEdges(boolean)   - 
Method in class org.proteinshader.gui. Renderer 
 Turns on or off the ability of the halftoning shaders to soften aliasing
    (jagged edges) by rendering translucent black silhouettes of tube and 
    ribbon segments (and using jitter) before calling on the halftoning shaders.
   Bond   - Class in  org.proteinshader.structure  Extends abstract class Drawable to add data that is unique to a bond
between two atoms.   Bond(Atom, Atom, BondEnum, VisibilityEnum)   - 
Constructor for class org.proteinshader.structure. Bond 
 Constructs a Bond for the source and destination Atoms given as
    arguments.
   BOND_VISIBILITY   - 
Static variable in class org.proteinshader.structure.io. PDBLineParser 
 The default visibilty for Bonds.
   BondDestroyerVisitor   - Class in  org.proteinshader.structure.visitor  Removes Bonds on all Atoms that it visits.   BondDestroyerVisitor()   - 
Constructor for class org.proteinshader.structure.visitor. BondDestroyerVisitor 
 Constructs a BondDestroyerVisitor.
   BondEnum   - Enum in  org.proteinshader.structure.enums  Provides an enumeration for molecular bonds.   BondGeneratorVisitor   - Class in  org.proteinshader.structure.visitor  Generates standard Bonds for AminoAcids and Waters.   BondGeneratorVisitor()   - 
Constructor for class org.proteinshader.structure.visitor. BondGeneratorVisitor 
 Constructs a BondGeneratorVisitor.
   BondLengthException   - Exception in  org.proteinshader.structure.exceptions  Can be used to report that a Bond is unacceptably short or long.   BondLengthException(double, double, double, AtomEnum, AtomEnum, int, int)   - 
Constructor for exception org.proteinshader.structure.exceptions. BondLengthException 
 Sets the default message to "A Bond length was unacceptable".
   BondLengthException(String, double, double, double, AtomEnum, AtomEnum, int, int)   - 
Constructor for exception org.proteinshader.structure.exceptions. BondLengthException 
 The message given this constructor can be retrieved using
    getMessage().
   BondModifier   - Class in  org.proteinshader.structure.visitor.modifiers  This helper class for the ModifierVisitor knows how to accept a Bond
object and modify its attributes.   BondModifier()   - 
Constructor for class org.proteinshader.structure.visitor.modifiers. BondModifier 
 Constructs a BondModifier.
   BondPredictor   - Class in  org.proteinshader.structure.visitor  Knows how to test if the distance between two Atoms is acceptable for
a covalent Bond.   BondPredictor()   - 
Constructor for class org.proteinshader.structure.visitor. BondPredictor 
 Constructs a BondPredictor with the Bond length tolerance
    and minimum acceptable length set to DEFAULT_TOLERANCE and
    DEFAULT_MIN_BOND_LENGTH, respectively.
   BondPredictor(double, double)   - 
Constructor for class org.proteinshader.structure.visitor. BondPredictor 
 Constructs a BondPredictor with the requested tolerance and
    minimum Bond length.
   BORDER_COLOR   - 
Static variable in class org.proteinshader.gui.components.controlpanel. ControlPanel 
 The border color for the control panel is light cyan
        (0.7, 1.0, 1.0).
   BOTTOM_MARGIN   - 
Static variable in class org.proteinshader.gui. ProteinShaderGUI 
 The bottom margin is the minimum number of
        pixels to leave between the top of the GUI
        window and the top of the monitor.
   BoundsVisitor   - Class in  org.proteinshader.structure.visitor  Knows how to find the min and max xyz-coordinate values for all Atoms
in each Model.   BoundsVisitor()   - 
Constructor for class org.proteinshader.structure.visitor. BoundsVisitor 
 Constructs a BoundsVisitor.
   BYTES_PER_PIXEL   - 
Static variable in class org.proteinshader.graphics.typography. GLF2GlyphSetFactory 
 The '.glf' image uses 2 bytes per pixel.
   BYTES_PER_PIXEL   - 
Static variable in class org.proteinshader.graphics.typography. GLF2Header 
 The mipmap uses two bytes per
        pixel (luminescence and alpha).
   BYTES_PER_PIXEL   - 
Static variable in class org.proteinshader.graphics.typography. TextLabelManager 
 The blank texture map has 2 bytes per pixel.
 
 
    
 C  
 
   cacheCylinderDisplayList(GL, Cylinder, CylinderListInfo)   - 
Method in class org.proteinshader.graphics.displaylists. CylinderReferences 
 Caches a new OpenGL display list for a BALLS_AND_STICKS cylinder or
    a STICKS cylinder with the requested number of slices and stacks.
   cacheDefaultCylinders(GL, Cylinder)   - 
Method in class org.proteinshader.graphics.displaylists. CylinderReferences 
 Caches a collection of cylinders using OpenGL display lists.
   cacheDefaultGeometricObjects(GL)   - 
Method in class org.proteinshader.graphics.adapter. StructureToGraphics 
 Uses the default values for tiling (slices and stacks) to cache
    geometric objects (spheres and cylinders) in the form of OpenGL
    display lists.
   cacheDefaultSpheres(GL, Sphere)   - 
Method in class org.proteinshader.graphics.displaylists. SphereReferences 
 Caches a collection of spheres using OpenGL display lists.
   cacheDisplayLists(GL, List<String>)   - 
Method in class org.proteinshader.graphics.typography. TextLabelManager 
 Caches an OpenGL display list holding glTexSubImage2D( bitmap )
    for each textLabel in the list given as an argument.
   cacheGeometricObject(GL, GeometricListInfo)   - 
Method in class org.proteinshader.graphics.adapter. StructureToGraphics 
 Caches a new OpenGL display list for a geometric object.
   cacheGeometricObject(GeometricListInfo)   - 
Method in interface org.proteinshader.gui. Mediator 
 Caches a new geomtric object (sphere or cylinder) in the form of
    an OpenGL display list and redraws the canvas.
   cacheGeometricObject(GeometricListInfo)   - 
Method in class org.proteinshader.gui. MediatorImpl 
 Caches a new geomtric object (sphere or cylinder) in the form of
    an OpenGL display list and redraws the canvas.
   cacheGeometricObject(GeometricListInfo)   - 
Method in class org.proteinshader.gui. Renderer 
 Stores a request to cache a new geomtric object so that the
    request can be processed the next time display() is called.
   cacheSegmentGeometry(GL, Model, StyleEnum)   - 
Method in class org.proteinshader.graphics.adapter. StructureToGraphics 
 Calls on the SegmentReferences object to cache OpenGL display
    lists for the Segments of the current Model.
   cacheSegmentGeometry(GL, Model, StyleEnum)   - 
Method in class org.proteinshader.graphics.displaylists. SegmentReferences 
 Caches an OpenGL display list for each Segment of each Region object in the
    Model.
   cacheSphereDisplayList(GL, Sphere, SphereListInfo)   - 
Method in class org.proteinshader.graphics.displaylists. SphereReferences 
 Caches a new OpenGL display list for a SPACE_FILLING sphere or a
    BALLS_AND_STICKS sphere with the requested tiling (number of slices
    and stacks).
   cacheTextLabels(GL, List<String>)   - 
Method in class org.proteinshader.graphics.adapter. StructureToGraphics 
 Informs the TextLabelManager that OpenGL display lists for text
    label sub need to be cached.
   cacheTextLabels(List<String>)   - 
Method in class org.proteinshader.gui. Renderer 
 Informs the Renderer that new textLabels should be cached because
    a new Structure has been loaded.
   calculateBounds()   - 
Method in class org.proteinshader.structure. Structure 
 Sets the min and max values for x, y, and z on each Model held by
    the Structure.
   calculateDepth(Drawable)   - 
Method in class org.proteinshader.gui.viewing. Camera 
 Calculates the depth (z-coordinate in camera space) of the xyz-center of 
    the Drawable object.
   calculateDepth(double, double, double)   - 
Method in class org.proteinshader.gui.viewing. Camera 
 Calculates the depth (z-coordinate in camera space) of the xyz-center of 
    the Drawable object.
   calculateDistance(Drawable)   - 
Method in class org.proteinshader.gui.viewing. Camera 
 Calculates the distance between the camera and the xyz-center of the 
    Drawable object by using the Pythagorean theorem.
   calculateDistance(double, double, double)   - 
Method in class org.proteinshader.gui.viewing. Camera 
 Calculates the distance between the camera and the xyz-center of the 
    Drawable object by using the Pythagorean theorem.
   calculateFrenetFrames()   - 
Method in class org.proteinshader.structure. Structure 
 Before Regions objects (Helix, BetaStrand, and Loop) are added
    to the Structure, a rotation (the equivalent to a discrete Frenet
    Frame) should be calculated for each AminoAcid.
   calculatePoint(double)   - 
Method in class org.proteinshader.math. Hermite 
 Uses Hermite interpolation to calculate a point on the curve
    between the start and end points given the constructor.
   calculateReverseTangent(double)   - 
Method in class org.proteinshader.math. Hermite 
 Gets the tangent from the calculateTangent() method and reverses
    its direction.
   calculateRotation(AminoAcid, AminoAcid, AminoAcid)   - 
Method in class org.proteinshader.structure.visitor. FrenetFrameGeneratorVisitor 
 Calculates the equivalent of a discrete Frenet frame (a rotation
    matrix [N B T]) and uses it to set the rotation attribute of the
    current AminoAcid.
   calculateRotation(Atom, Atom, Atom)   - 
Method in class org.proteinshader.structure.visitor. FrenetFrameGeneratorVisitor 
 Calculates a rotation matrix [N B T] based on the xyz-centers of
    the three Atoms given as arguments, and then converts the rotation
    matrix into a Quaternion that is returned.
   calculateTangent(double)   - 
Method in class org.proteinshader.math. Hermite 
 Uses Hermite interpolation to calculate the tangent of a point on
    the cubic equation between the start and end points given the
    constructor.
   calculateTimeAndRotation()   - 
Method in class org.proteinshader.gui.viewing. Rotation 
 Calculates an x-axis and y-axis rotation based on the current
    system time and the previous time this method was called.
   calculateTranslation(double)   - 
Method in class org.proteinshader.math. Hermite 
 If the xyz-point obtained by calculatePoint() is going to be
    used to translate an object from the origin to the point, then it
    is convenient for a vector to be returned instead of a point.
   Camera   - Class in  org.proteinshader.gui.viewing  Used to store and manipulate camera information.   Camera()   - 
Constructor for class org.proteinshader.gui.viewing. Camera 
 Constructs a Renderer.
   CAMERA_XY_SCALE   - 
Static variable in class org.proteinshader.gui.listeners. CanvasMouseListener 
 The camera xy scale factor is used to slow down
        camera movement in the xy-plane.
   CAMERA_ZOOM_SCALE   - 
Static variable in class org.proteinshader.gui.listeners. CanvasMouseListener 
 The camera zoom factor is used to slow down camera movement
        in the z-axis (for mouse dragging).
   CanvasKeyListener   - Class in  org.proteinshader.gui.listeners  Responds to keyboard shortcuts when the canvas has the focus.   CanvasKeyListener(Mediator, Renderer, GLCanvas)   - 
Constructor for class org.proteinshader.gui.listeners. CanvasKeyListener 
 Constructs a CanvasKeyListener.
   CanvasMouseListener   - Class in  org.proteinshader.gui.listeners  Converts mouse movements across the canvas to image rotations or
camera movements.   CanvasMouseListener(Renderer)   - 
Constructor for class org.proteinshader.gui.listeners. CanvasMouseListener 
 Constructs a CanvasMouseListener.
   CanvasMouseListener.Mode   - Enum in  org.proteinshader.gui.listeners  The mode controls image rotation and camera movements.   capture(GL, GLAutoDrawable)   - 
Method in class org.proteinshader.gui.viewing. ScreenShot 
 Take a screen shot of the canvas and saves it to a PNG, JPG, or GIF file.
   CartoonColorPanel   - Class in  org.proteinshader.gui.components.controlpanel  This control panel allows the user to modify the color of Segments.   CartoonColorPanel(Mediator, Frame, RadioPanel)   - 
Constructor for class org.proteinshader.gui.components.controlpanel. CartoonColorPanel 
 Constructs a CartoonColorPanel.
   CartoonSideChainsPanel   - Class in  org.proteinshader.gui.components.controlpanel  This control panel allows the user to display side
chain Atoms and Bonds while in a Cartoon-style display.   CartoonSideChainsPanel(Mediator, Frame, RadioPanel)   - 
Constructor for class org.proteinshader.gui.components.controlpanel. CartoonSideChainsPanel 
 Constructs a CartoonSideChainsPanel.
   CartoonSideChainsPanelListenerFactory   - Class in  org.proteinshader.gui.listeners.controlpanel  Creates the listener for the CartoonSideChainsPanel.   CartoonSideChainsPanelListenerFactory(Mediator, CartoonSideChainsPanel)   - 
Constructor for class org.proteinshader.gui.listeners.controlpanel. CartoonSideChainsPanelListenerFactory 
 Constructs a CartoonSideChainsPanelListenerFactory.
   cartoonSubmenu   - 
Static variable in class org.proteinshader.gui.components.menubar. StyleMenu 
 This list determines which menu items are placed in the
        'Cartoon' submenu (currently Tubes, Ribbons, and Frenet
        Frames).
   CartoonVisibilityPanel   - Class in  org.proteinshader.gui.components.controlpanel  This control panel allows the user to modify the visibility status and
alpha value of Segments.   CartoonVisibilityPanel(Mediator, Frame, RadioPanel)   - 
Constructor for class org.proteinshader.gui.components.controlpanel. CartoonVisibilityPanel 
 Constructs a CartoonVisibilityPanel.
   Category   - Class in  org.proteinshader.structure  A Category object serves as a container for the Records in a PDB
structure entry.   Category(String)   - 
Constructor for class org.proteinshader.structure. Category 
 Constructs a Category with the name specified as an argument.
   CategoryEnum   - Enum in  org.proteinshader.structure.io.enums  This file provides an enumeration of the categories that RecordEnums
fit into.   CategoryEnumDemo   - Class in  org.proteinshader.structure.io.enums  CategoryEnumDemo is for testing CategoryEnum.   CategoryEnumDemo()   - 
Constructor for class org.proteinshader.structure.io.enums. CategoryEnumDemo 
  
   CenteredListCellRenderer   - Class in  org.proteinshader.gui.utils  Centers the horizontal alignment of text in a JList or JComboBox.   CenteredListCellRenderer()   - 
Constructor for class org.proteinshader.gui.utils. CenteredListCellRenderer 
 Creates a CenteredListCellRenderer.
   Chain   - Class in  org.proteinshader.structure  A Chain serves as a container for Residues (AminoAcid, Heterogen, and
Water) and Regions (Loop, Helix, and BetaStrand).   Chain(String, String, String)   - 
Constructor for class org.proteinshader.structure. Chain 
 Constructs a Chain with the requested chainID, modelID, and
    structureID.
   changeSubpanel()   - 
Method in class org.proteinshader.gui.components.controlpanel. ModifierPanel 
 Sets the currently displayed subpanel to the panel selected in the
    subpanel menu at the top of the ModifierPanel.
   CHAR_INFO_BYTES   - 
Static variable in class org.proteinshader.graphics.typography. GLF2Header 
 Each character info block in a '.glf' file has 24 bytes.
   CHAR_PER_LINE   - 
Static variable in class org.proteinshader.structure.visitor. SFWriterVisitor 
  
   cleanUpMemory(GL)   - 
Method in class org.proteinshader.graphics.displaylists. SegmentReferences 
 Cleans up OpenGL graphics card memory allocated for OpenGL display
    lists for the previous Model.
   clear(GL)   - 
Method in class org.proteinshader.graphics.displaylists. SegmentListInfo 
 Frees graphics card memory for OpenGL display lists.
   clear()   - 
Method in class org.proteinshader.structure. Record 
 Clears all lines from the Record.
   clear()   - 
Method in class org.proteinshader.structure.visitor.modifiers. AtomModifier 
 Clears the memory of what modifications have been requested.
   clear()   - 
Method in class org.proteinshader.structure.visitor.modifiers. DrawableModifier 
 Clears the memory of what modifications have been requested.
   clear()   - 
Method in class org.proteinshader.structure.visitor.modifiers. SegmentModifier 
 Clears the memory of what modifications have been requested.
   clear()   - 
Method in class org.proteinshader.structure.visitor. ModifierVisitor 
 Clears all memory of previously added Modifiers.
   clear()   - 
Method in class org.proteinshader.structure.visitor. Visitor 
 Resets Visitor to default of ResidueMode.ALL,
    RegionMode.NO_REGIONS, AAPortionMode.ENTIRE_AA, and restrictions
    (AminoAcid type, Atom type, and Atom ID) to null.
   clearAllBonds()   - 
Method in class org.proteinshader.structure. Structure 
 Clears all Bonds (AminoAcids, Heterogens, and Waters) from the
    Structure.
   clearAtoms()   - 
Method in class org.proteinshader.gui.components.controlpanel. SelectorPanel 
 Clears the Atom list.
   clearAtomSerialNoCache()   - 
Method in class org.proteinshader.structure. Structure 
 Clears the cache that remembers Atom serial numbers and allows
    them to be used to retrieve an Atom.
   clearBonds()   - 
Method in class org.proteinshader.structure. Atom 
 Deletes all Bonds held by this Atom.
   clearBonds(ResidueMode)   - 
Method in class org.proteinshader.structure. Structure 
 Clears Bonds from the Structure.
   clearChains()   - 
Method in class org.proteinshader.gui.components.controlpanel. SelectorPanel 
 Clears the Chain menu, Residue list, and Atom list.
   clearColorChangeMemory()   - 
Method in class org.proteinshader.structure.visitor.modifiers. DrawableModifier 
 Clears any memory of color change that have been requested.
   clearList()   - 
Method in class org.proteinshader.structure.visitor. AminoAcidLabelVisitor 
 Clears the list of labels.
   clearListsOfVisibles()   - 
Method in class org.proteinshader.structure. Model 
 Clears the lists of opaque and translucent Drawable objects.
   clearModels()   - 
Method in class org.proteinshader.gui.components.controlpanel. SelectorPanel 
 Clears all menus and lists.
   clearResidues()   - 
Method in class org.proteinshader.gui.components.controlpanel. SelectorPanel 
 Clears the Residue list and Atom list.
   clearTimer()   - 
Method in class org.proteinshader.gui.components. SpringLoadedJFrame 
 Stops the timer (if it is running) and sets it to null.
   clickStartAnimation()   - 
Method in class org.proteinshader.gui.components.controlpanel. ControlPanel 
 Calls on the startAnimation() method of the MotionPanel (equivalent to
    clicking the Start button on the MotionPanel).
   clickStartAnimation()   - 
Method in class org.proteinshader.gui.components.controlpanel. ModifierPanel 
 Calls on the startAnimation() method of the MotionPanel (equivalent to
    clicking the Start button on the MotionPanel).
   clickStartAnimation()   - 
Method in interface org.proteinshader.gui. Mediator 
 Calls on the startAnimation() method of the MotionPanel (equivalent to
    clicking the Start button on the MotionPanel).
   clickStartAnimation()   - 
Method in class org.proteinshader.gui. MediatorImpl 
 Calls on the startAnimation() method of the MotionPanel (equivalent to
    clicking the Start button on the MotionPanel).
   clickStopAnimation()   - 
Method in class org.proteinshader.gui.components.controlpanel. ControlPanel 
 Calls on the stopAnimation() method of the MotionPanel (equivalent to
    clicking the Stop button on the MotionPanel).
   clickStopAnimation()   - 
Method in class org.proteinshader.gui.components.controlpanel. ModifierPanel 
 Calls on the stopAnimation() method of the MotionPanel (equivalent to
    clicking the Stop button on the MotionPanel).
   clickStopAnimation()   - 
Method in interface org.proteinshader.gui. Mediator 
 Calls on the stopAnimation() method of the MotionPanel (equivalent to
    clicking the Stop button on the MotionPanel).
   clickStopAnimation()   - 
Method in class org.proteinshader.gui. MediatorImpl 
 Calls on the stopAnimation() method of the MotionPanel (equivalent to
    clicking the Stop button on the MotionPanel).
   clone()   - 
Method in class org.proteinshader.math. Hermite 
 Creates a clone of the calling Hermite object and returns it.
   clone()   - 
Method in class org.proteinshader.math. LocalFrame 
 Returns a clone of the calling LocalFrame.
   clone()   - 
Method in class org.proteinshader.math. Point3d 
 Returns a clone of this point.
   clone()   - 
Method in class org.proteinshader.math. Quaternion 
 Creates a clone of this quaternion and returns it.
   clone()   - 
Method in class org.proteinshader.math. Vec3d 
 Returns a clone of the calling vector.
   closeAtomListDialog()   - 
Method in class org.proteinshader.gui.components.controlpanel. SelectorPanel 
 Sets the Atom list dialog to invisible and enables the "Atoms"
    button.
   closeControlFrame()   - 
Method in interface org.proteinshader.gui. Mediator 
 Closes the spring-loaded frame holding the control panel.
   closeControlFrame()   - 
Method in class org.proteinshader.gui. MediatorImpl 
 Closes the spring-loaded frame holding the ControlPanel.
   closeControlFrame()   - 
Method in class org.proteinshader.gui. ProteinShaderGUI 
 Closes the spring-loaded frame holding the control panel.
   closeOutputFile()   - 
Method in class org.proteinshader.structure.visitor. SFWriterVisitor 
 This method flushes the output buffer to make sure that all data
    is written before closing the output file.
   COEFFICIENT   - 
Static variable in class org.proteinshader.graphics.displaylists. CylinderReferences 
 The empirically-determined coefficient for the power
        equation is 29.059.
   COEFFICIENT   - 
Static variable in class org.proteinshader.graphics.displaylists. SphereReferences 
 The empirically-determined coefficient for the power
        equation is 226.1.
   ColorOutOfRangeException   - Exception in  org.proteinshader.structure.exceptions  Used to report that a color value is out of range.   ColorOutOfRangeException()   - 
Constructor for exception org.proteinshader.structure.exceptions. ColorOutOfRangeException 
 Sets the default message to "ERROR: A color value was out of
    range".
   ColorOutOfRangeException(String)   - 
Constructor for exception org.proteinshader.structure.exceptions. ColorOutOfRangeException 
 The message given this constructor can be retrieved using
    getMessage().
   ColorPanel   - Class in  org.proteinshader.gui.components.controlpanel  This control panel allows the user to modify the color of Drawable
objects.   ColorPanel(Mediator, Frame, RadioPanel, String, String)   - 
Constructor for class org.proteinshader.gui.components.controlpanel. ColorPanel 
 Constructs a ColorPanel.
   ColorPanelListenerFactory   - Class in  org.proteinshader.gui.listeners.controlpanel  Creates the listener for the  ColorPanel.   ColorPanelListenerFactory(Mediator, ColorPanel)   - 
Constructor for class org.proteinshader.gui.listeners.controlpanel. ColorPanelListenerFactory 
 Constructs a ColorPanelListenerFactory.
   compareTo(Texture)   - 
Method in class org.proteinshader.graphics.textures. Texture 
 Compares this Texture object to the Texture object given as an
    argument by making a lexicographic comparison of their menu names.
   compile(GL, ShaderEnum)   - 
Method in class org.proteinshader.graphics.shader. ShaderProgramFactory 
 Reads a pair of vertex and fragment shader files and then compiles
    and links them.
   compileAllShaders(GL)   - 
Method in class org.proteinshader.graphics.shader. ShaderProgramFactory 
 Compiles all shaders pairs defined by the ShaderEnum.
   compileShaders(GL)   - 
Method in class org.proteinshader.graphics.adapter. StructureToGraphics 
 Compiles the OpenGL Shading Language vertex and fragment shaders
    that will be needed for lighting calculations and texture mapping.
   compileShaders(GL)   - 
Method in class org.proteinshader.graphics.shader. ShaderManager 
 Compiles the OpenGL Shading Language vertex and fragment shaders
    needed for Phong lighting, text label mapping, and real-time
    hafltoning.
   componentHidden(ComponentEvent)   - 
Method in class org.proteinshader.gui.components. SpringLoadedJFrame 
 This method is called automatically when the parent frame is made
    invisible, so it will hide this frame if it is visible.
   componentMoved(ComponentEvent)   - 
Method in class org.proteinshader.gui.components. SpringLoadedJFrame 
 This method is called automatically when the parent frame's
    position changes, and it will recalculate this frame's bounds.
   componentResized(ComponentEvent)   - 
Method in class org.proteinshader.gui.components. SpringLoadedJFrame 
 This method is called automatically when the parent frame's size
    changes, and it will recalculate this frame's bounds.
   componentShown(ComponentEvent)   - 
Method in class org.proteinshader.gui.components. SpringLoadedJFrame 
 This method is called automatically when the parent frame has been
    made visible, so it will set this frame visible if it had been
    hidden with the parent.
   ConfigReader   - Interface in  org.proteinshader.graphics.textures  Reads a configuration file to obtain a list of menu names
and matching filenames for textures.   conjugate()   - 
Method in class org.proteinshader.math. Quaternion 
 Creates a clone of the calling Quaternion and then converts it
    to the conjugate, q'.
   conjugateMe()   - 
Method in class org.proteinshader.math. Quaternion 
 Converts the calling Quaternion to its conjugate.
   ControlPanel   - Class in  org.proteinshader.gui.components.controlpanel  Presents the SelectorPanel and ModifierPanel.   ControlPanel(Mediator, Frame)   - 
Constructor for class org.proteinshader.gui.components.controlpanel. ControlPanel 
 Constructs a ControlPanel.
   convertToAngstroms(double, double)   - 
Method in class org.proteinshader.gui.viewing. Camera 
 Converts pixels to angstroms based on depth in camera space.
   convertToQuaternionAndPrint(Vec3d, Vec3d, Vec3d)   - 
Method in class org.proteinshader.math. QuaternionDemo 
 Converts rotation matrix [N B T] to a quaternion and prints it.
   CPKColorEnum   - Enum in  org.proteinshader.structure.enums  Provides an enumeration of the CPK color scheme that is needed by
AtomEnum.   creatCustomMenuItemListener()   - 
Method in class org.proteinshader.gui.listeners.menubar. BackgroundMenuListenerFactory 
 Creates an action listener for the 'Custom' menu items.
   createAboutMenuItemListener()   - 
Method in class org.proteinshader.gui.listeners.menubar. HelpMenuListenerFactory 
 Creates an action listener for the 'About' menu item.
   createAlphaSliderChangeListener()   - 
Method in class org.proteinshader.gui.listeners.controlpanel. VisibilityPanelListenerFactory 
 Creates a change listener for the alpha slider.
   createAminoAcidsCheckBoxListener()   - 
Method in class org.proteinshader.gui.listeners.menubar. VisibilityMenuListenerFactory 
 Creates an action listener for the 'Amino Acids' check box menu
    item.
   createApplyButtonActionListener()   - 
Method in class org.proteinshader.gui.listeners.controlpanel. OptimizePanelListenerFactory 
 Creates an action listener for the "Apply" button.
   createApplyButtonActionListener()   - 
Method in class org.proteinshader.gui.listeners.controlpanel. ScalePanelListenerFactory 
 Creates an action listener for the 'Apply' button.
   createAtomDialogBoxListener()   - 
Method in class org.proteinshader.gui.listeners.controlpanel. SelectorPanelListenerFactory 
 Creates a window listener for the Atom list dialog box.
   createAtomListListener()   - 
Method in class org.proteinshader.gui.listeners.controlpanel. SelectorPanelListenerFactory 
 Creates a list selection listener for the Atom list.
   createAtomsButtonListener()   - 
Method in class org.proteinshader.gui.listeners.controlpanel. SelectorPanelListenerFactory 
 Creates an action listener for the "Atoms" button.
   createAutoTilingButtonItemListener()   - 
Method in class org.proteinshader.gui.listeners.controlpanel. OptimizePanelListenerFactory 
 Creates a change listener for the "Auto Tiling" check box button.
   createBallsAndSticksButtonActionListener()   - 
Method in class org.proteinshader.gui.listeners.controlpanel. CartoonSideChainsPanelListenerFactory 
 Creates an action listener for the "Balls and Sticks" button.
   createBendMenuActionListener()   - 
Method in class org.proteinshader.gui.listeners.controlpanel. DecorationsPanelListenerFactory 
 Creates an action listener for the 'Bend Texture:' menu.
   createBendTextures(GL)   - 
Method in class org.proteinshader.graphics.textures. TextureFactory 
 Creates Texture objects based on files named in BEND_CONFIG.
   createBlackMenuItemListener()   - 
Method in class org.proteinshader.gui.listeners.menubar. BackgroundMenuListenerFactory 
 Creates an action listener for the 'Black' menu items.
   createChainMenuListener()   - 
Method in class org.proteinshader.gui.listeners.controlpanel. SelectorPanelListenerFactory 
 Creates an action listener for the Chain menu.
   createChooserButtonActionListener()   - 
Method in class org.proteinshader.gui.listeners.controlpanel. ColorPanelListenerFactory 
 Creates an action listener for the "Chooser" button.
   createChooserChangeListener()   - 
Method in class org.proteinshader.gui.listeners.controlpanel. ColorPanelListenerFactory 
 Creates a ChangeListener that will get the color selection from
    the JColorChooser and calls on the ColorPanel to change the color
    of whatever items are currently selected.
   createChooserChangeListener()   - 
Method in class org.proteinshader.gui.listeners.menubar. BackgroundMenuListenerFactory 
 Creates a ChangeListener that will get the color selection from
    the JColorChooser and calls on the Mediator to change the
    background color of the canvas.
   createCloseButtonListener()   - 
Method in class org.proteinshader.gui.listeners.controlpanel. SelectorPanelListenerFactory 
 Creates an action listener for the "Close" button on the Atom list
    dialog box.
   createCloseButtonListener()   - 
Method in class org.proteinshader.gui.listeners.menubar. AboutBoxListenerFactory 
 Creates an action listener for the 'Close' button.
   createControlFrameListener()   - 
Method in class org.proteinshader.gui.listeners. WindowListenerFactory 
 Creates a window listener for the spring-loaded frame that holds
    the control panel.
   createControlPanelCheckBoxListener()   - 
Method in class org.proteinshader.gui.listeners.menubar. ToolsMenuListenerFactory 
 Creates an item listener for the "Control Panel" check box menu
    item.
   createControlsMenuListener()   - 
Method in class org.proteinshader.gui.listeners.controlpanel. ModifierPanelListenerFactory 
 Creates an action listener for the menu of control panels.
   createCylinderCapsButtonActionListener()   - 
Method in class org.proteinshader.gui.listeners.controlpanel. OptimizePanelListenerFactory 
 Creates an action listener for the "Cylinder Caps"  radio button.
   createCylinderCapsMenuActionListener()   - 
Method in class org.proteinshader.gui.listeners.controlpanel. OptimizePanelListenerFactory 
 Creates an action listener for the "Cylinder Caps" menu.
   createCylindersButtonActionListener()   - 
Method in class org.proteinshader.gui.listeners.controlpanel. OptimizePanelListenerFactory 
 Creates an action listener for the "Cylinders"  radio button.
   createCylindersMenuActionListener()   - 
Method in class org.proteinshader.gui.listeners.controlpanel. OptimizePanelListenerFactory 
 Creates an action listener for the "Cylinders" menu.
   createDefault1ButtonActionListener()   - 
Method in class org.proteinshader.gui.listeners.controlpanel. ColorPanelListenerFactory 
 Creates an action listener for the first default button.
   createDefault2ButtonActionListener()   - 
Method in class org.proteinshader.gui.listeners.controlpanel. ColorPanelListenerFactory 
 Creates an action listener for the second default button.
   createDefaultButtonActionListener()   - 
Method in class org.proteinshader.gui.listeners.controlpanel. OptimizePanelListenerFactory 
 Creates an action listener for the "Default" button.
   createDefaultButtonActionListener()   - 
Method in class org.proteinshader.gui.listeners.controlpanel. ScalePanelListenerFactory 
 Creates an action listener for the 'Default' button.
   createDisplayList(GL, CylinderListInfo)   - 
Method in class org.proteinshader.graphics. Cylinder 
 Creates an OpenGL display list for drawing a cylinder with
    the radii, slices, stacks, and end caps specified in the
    CylinderListInfo object given as an argument.
   createDisplayList(GL, double, double, double, int, int, boolean, boolean, int, int)   - 
Method in class org.proteinshader.graphics. Cylinder 
 Stores the OpenGL commands for drawing a cylinder as an OpenGL
    display list.
   createDisplayList(GL, LocalFrame[])   - 
Method in class org.proteinshader.graphics. ExtrudedShape 
 Uses an array of LocalFrame objects to draw a segment of a tube
    and saves the geometry (a collection of vertices) as an OpenGL
    display list.
   createDisplayList(GL, SphereListInfo)   - 
Method in class org.proteinshader.graphics. Sphere 
 Creates an OpenGL display list for drawing a sphere with the
    radius, number of slices, and number of stacks specified in the
    SphereListInfo object given as an argument.
   createDisplayList(GL, double, int, int)   - 
Method in class org.proteinshader.graphics. Sphere 
 Creates an OpenGL display list that holds the commands for drawing
    a sphere.
   createDisplayLists(GL, LocalFrame[], SegmentListInfo)   - 
Method in class org.proteinshader.graphics. ExtrudedShape 
 Creates three OpenGL display lists: one created by calling draw(),
    one created by calling drawStartCap(), and one created by calling
    drawEndCap().
   createDisplayLists(GL, LocalFrame[], SegmentListInfo)   - 
Method in class org.proteinshader.graphics. Ribbon 
 Creates an OpenGL display list for the thin sides of the ribbon,
    and also calls on the createDisplayLists() method of superclass
    ExtrudedShape to save the OpenGL display lists for the main
    (broad surface) part of the ribbon and for the start and end caps.
   createEndCapDisplayList(GL, LocalFrame[])   - 
Method in class org.proteinshader.graphics. ExtrudedShape 
 Uses the last LocalFrame in the array to position a segment end
    cap that is saved as an OpenGL display list.
   createExtraLinesCheckBoxActionListener()   - 
Method in class org.proteinshader.gui.listeners.controlpanel. DecorationsPanelListenerFactory 
 Creates and action listener for the "Extra Lines" check box.
   createGlyphSetFactory(String, String)   - 
Method in class org.proteinshader.graphics.typography. GlyphSetFactoryFactory 
 Uses Java reflection to load the concrete GlyphSetFactory that
    is requested in the configuration file.
   createGraphicsCardMenuItemListener()   - 
Method in class org.proteinshader.gui.listeners.menubar. HelpMenuListenerFactory 
 Creates an action listener for the 'Graphic Card Info' menu item.
   createGrayMenuItemListener()   - 
Method in class org.proteinshader.gui.listeners.menubar. BackgroundMenuListenerFactory 
 Creates an action listener for the 'Gray' menu items.
   createHalftoningButtonActionListener()   - 
Method in class org.proteinshader.gui.listeners.controlpanel. DecorationsPanelListenerFactory 
 Creates an action listener for the 'Halftoning' radio button.
   createHalftoningCheckBoxListener()   - 
Method in class org.proteinshader.gui.listeners.controlpanel. AntialiasingPanelListenerFactory 
 Creates a change listener for the 'Antialias halftoning images' check box.
   createHalftoningMenuActionListener()   - 
Method in class org.proteinshader.gui.listeners.controlpanel. DecorationsPanelListenerFactory 
 Creates an action listener for the 'Halftoning Texture:' menu.
   createHalftoningTextures(GL)   - 
Method in class org.proteinshader.graphics.textures. TextureFactory 
 Creates Texture objects based on files named in HALFTONING_CONFIG.
   createHeterogensCheckBoxListener()   - 
Method in class org.proteinshader.gui.listeners.menubar. VisibilityMenuListenerFactory 
 Creates an action listener for the 'Heterogens' check box menu
    item.
   createImageFileMenuItemListener()   - 
Method in class org.proteinshader.gui.listeners.menubar. FileMenuListenerFactory 
 Creates an action listener for the "PNG File..." menu item.
   createInvisibleButtonActionListener()   - 
Method in class org.proteinshader.gui.listeners.controlpanel. VisibilityPanelListenerFactory 
 Creates an action listener for the "Invisible" button.
   createJitterMenuActionListener()   - 
Method in class org.proteinshader.gui.listeners.controlpanel. AntialiasingPanelListenerFactory 
 Creates a listener for the Jitter menu.
   createLabel(String, int, int)   - 
Method in class org.proteinshader.graphics.typography. TextLabelFactory 
 Uses Glyph objects to create a TextLabel with a Buffer containing
    an image based on the text String given as an argument.
   createLightGrayMenuItemListener()   - 
Method in class org.proteinshader.gui.listeners.menubar. BackgroundMenuListenerFactory 
 Creates an action listener for the 'Light Gray' menu items.
   createLocalHelpMenuItemListener()   - 
Method in class org.proteinshader.gui.listeners.menubar. HelpMenuListenerFactory 
 Creates an action listener for the 'Open Local Help Files in Web Browser' 
    menu item.
   createMenuActionListener()   - 
Method in class org.proteinshader.gui.listeners.controlpanel. RadioPanelListenerFactory 
 Creates an action listener for the combo box menus in the
    RadioPanel.
   createModelMenuListener()   - 
Method in class org.proteinshader.gui.listeners.controlpanel. SelectorPanelListenerFactory 
 Creates an action listener for the Model menu.
   createNoneButtonActionListener()   - 
Method in class org.proteinshader.gui.listeners.controlpanel. CartoonSideChainsPanelListenerFactory 
 Creates an action listener for the "Space Filling" button.
   createOpaqueButtonActionListener()   - 
Method in class org.proteinshader.gui.listeners.controlpanel. VisibilityPanelListenerFactory 
 Creates an action listener for the "Opaque" button.
   createOpenMenuItemListener()   - 
Method in class org.proteinshader.gui.listeners.menubar. FileMenuListenerFactory 
 Creates an action listener for the "Open" menu item.
   createOrientationMenuItemsListener()   - 
Method in class org.proteinshader.gui.listeners.menubar. OrientationMenuListenerFactory 
 Creates an action listener for the OrientationEnum menu items.
   createPatternsButtonActionListener()   - 
Method in class org.proteinshader.gui.listeners.controlpanel. DecorationsPanelListenerFactory 
 Creates an action listener for the 'Patterns' radio button.
   createPatternsMenuActionListener()   - 
Method in class org.proteinshader.gui.listeners.controlpanel. DecorationsPanelListenerFactory 
 Creates an action listener for the 'Patterns' texture menu.
   createPatternsTextures(GL)   - 
Method in class org.proteinshader.graphics.textures. TextureFactory 
 Creates Texture objects based on files named in PATTERNS_CONFIG.
   createPlainButtonActionListener()   - 
Method in class org.proteinshader.gui.listeners.controlpanel. DecorationsPanelListenerFactory 
 Creates an action listener for the 'Plain' radio button.
   createQuitMenuItemListener()   - 
Method in class org.proteinshader.gui.listeners.menubar. FileMenuListenerFactory 
 Creates an action listener for the "Quit" menu item.
   createRadioButtonListener()   - 
Method in class org.proteinshader.gui.listeners.menubar. StyleMenuListenerFactory 
 Creates the action listener for all radio buttons in the StyleMenu.
   createRadioButtonsActionListener()   - 
Method in class org.proteinshader.gui.listeners.controlpanel. RadioPanelListenerFactory 
 Creates an action listener for the radio buttons.
   createRepeats(char, int)   - 
Method in class org.proteinshader.math. LocalFrameDemo 
 Returns a String with the character given as an argument repeated
    n number of times.
   createRepeats(char, int)   - 
Method in class org.proteinshader.math. QuaternionDemo 
 Returns a String with the character given as an argument repeated
    n number of times.
   createRepeats(char, int)   - 
Method in class org.proteinshader.math. SlerpDemo 
 Returns a String with the character given as an argument repeated
    n number of times.
   createResidueListListener()   - 
Method in class org.proteinshader.gui.listeners.controlpanel. SelectorPanelListenerFactory 
 Creates a list selection listener for the Residue list.
   createResidueMenuListener()   - 
Method in class org.proteinshader.gui.listeners.controlpanel. SelectorPanelListenerFactory 
 Creates an action listener for the Residue type menu.
   createSceneCheckBoxListener()   - 
Method in class org.proteinshader.gui.listeners.controlpanel. AntialiasingPanelListenerFactory 
 Creates a change listener for the 'Antialias entire scene' check box.
   createSegments(Region)   - 
Method in class org.proteinshader.structure.factory. BetaStrandSegmentFactory 
 After using the superclass createSegments() method, the
    LocalFrames held by some of the Segment objects will be modified
    so that beta-strands will have a pleated effect rather than a
    twisted ribbon appearance.
   createSegments(Region)   - 
Method in class org.proteinshader.structure.factory. SegmentFactory 
 Creates the Segment objects for a Region.
   createSegments(SegmentFactory)   - 
Method in class org.proteinshader.structure. Region 
 This protected method should be called by the constructor of
    concrete subclasses of Region so that the Segment objects for
    the Region can be created with the appropriate type of
    SegmentFactory.
   createShaderProgram(GL, String, String, ShaderEnum)   - 
Method in class org.proteinshader.graphics.shader. ShaderProgramFactory 
 Reads source code files for a vertex shader and a fragment shader
    and creates an OpenGL shader program object.
   createShaderProgram(GL, File, File, ShaderEnum)   - 
Method in class org.proteinshader.graphics.shader. ShaderProgramFactory 
 Reads source code files for a vertex shader and a fragment shader
    and creates an OpenGL shader program object.
   createSourceForgeMenuItemListener()   - 
Method in class org.proteinshader.gui.listeners.menubar. HelpMenuListenerFactory 
 Creates an action listener for the 'Go to ProteinShader Web Site' menu item.
   createSpaceFillingButtonActionListener()   - 
Method in class org.proteinshader.gui.listeners.controlpanel. CartoonSideChainsPanelListenerFactory 
 Creates an action listener for the "Space Filling" button.
   createSpheresButtonActionListener()   - 
Method in class org.proteinshader.gui.listeners.controlpanel. OptimizePanelListenerFactory 
 Creates an action listener for the "Spheres"  radio button.
   createSpheresMenuActionListener()   - 
Method in class org.proteinshader.gui.listeners.controlpanel. OptimizePanelListenerFactory 
 Creates an action listener for the "Spheres" menu.
   createSpinnerChangeListener()   - 
Method in class org.proteinshader.gui.listeners.controlpanel. OptimizePanelListenerFactory 
 Creates a change listener for a spinner.
   createSpinnerChangeListener()   - 
Method in class org.proteinshader.gui.listeners.controlpanel. ScalePanelListenerFactory 
 Creates an change listener for a spinner.
   createStartButtonActionListener()   - 
Method in class org.proteinshader.gui.listeners.controlpanel. MotionPanelListenerFactory 
 Creates an action listener for the 'Start' button.
   createStartCapDisplayList(GL, LocalFrame[])   - 
Method in class org.proteinshader.graphics. ExtrudedShape 
 Uses the first LocalFrame in the array to position a segment start
    cap that is saved as an OpenGL display list.
   createSticksButtonActionListener()   - 
Method in class org.proteinshader.gui.listeners.controlpanel. CartoonSideChainsPanelListenerFactory 
 Creates an action listener for the "Sticks" button.
   createStopButtonActionListener()   - 
Method in class org.proteinshader.gui.listeners.controlpanel. MotionPanelListenerFactory 
 Creates an action listener for the 'Stop' button.
   createTextFieldActionListener()   - 
Method in class org.proteinshader.gui.listeners.controlpanel. MotionPanelListenerFactory 
 Creates an action listener for the 'X-Axis' text field.
   createTextLabelsButtonActionListener()   - 
Method in class org.proteinshader.gui.listeners.controlpanel. DecorationsPanelListenerFactory 
 Creates an action listener for the 'Text Labels' radio button.
   createTextures(GL, String, String)   - 
Method in class org.proteinshader.graphics.textures. TextureFactory 
 Creates Texture objects based on files named in the CONFIG_FILE.
   createThinSidesDisplayList(GL, LocalFrame[])   - 
Method in class org.proteinshader.graphics. Ribbon 
 Caches an OpenGL display list for the thin sides of a segment of a
    ribbon.
   createTranslucentButtonActionListener()   - 
Method in class org.proteinshader.gui.listeners.controlpanel. VisibilityPanelListenerFactory 
 Creates an action listener for the "Translucent" button.
   createWatersCheckBoxListener()   - 
Method in class org.proteinshader.gui.listeners.menubar. VisibilityMenuListenerFactory 
 Creates an action listener for the 'Waters' check box menu item.
   createWebSiteButtonListener()   - 
Method in class org.proteinshader.gui.listeners.menubar. AboutBoxListenerFactory 
 Creates an action listener for the 'Go to Web Site' button.
   createWhiteMenuItemListener()   - 
Method in class org.proteinshader.gui.listeners.menubar. BackgroundMenuListenerFactory 
 Creates an action listener for the 'White' menu items.
   createWireframeButtonActionListener()   - 
Method in class org.proteinshader.gui.listeners.controlpanel. DecorationsPanelListenerFactory 
 Creates an action listener for the 'Wireframe' radio button.
   createXAxisSliderChangeListener()   - 
Method in class org.proteinshader.gui.listeners.controlpanel. MotionPanelListenerFactory 
 Creates a change listener for the 'X-Axis' slider that is used to
    adjust the rotation speed.
   createYAxisSliderChangeListener()   - 
Method in class org.proteinshader.gui.listeners.controlpanel. MotionPanelListenerFactory 
 Creates a change listener for the 'Y-Axis' slider that is used to
    adjust the rotation speed.
   cross(Vec3d)   - 
Method in class org.proteinshader.math. Vec3d 
 Creates a new vector by taking the cross product of the calling
    vector and the vector given as an argument.
   crossMe(Vec3d)   - 
Method in class org.proteinshader.math. Vec3d 
 Modifies the calling vector by calculating the cross product of
    the calling vector and the vector given as an argument and storing
    the result in the calling vector.
   Cylinder   - Class in  org.proteinshader.graphics  Knows how to draw a simple cylinder useful for representing the bonds
between atoms.   Cylinder()   - 
Constructor for class org.proteinshader.graphics. Cylinder 
 Constructs a Cylinder.
   CylinderListInfo   - Class in  org.proteinshader.graphics.displaylists  Stores information on an OpenGL display list for a cylinder.   CylinderListInfo()   - 
Constructor for class org.proteinshader.graphics.displaylists. CylinderListInfo 
 Constructs a CylinderListInfo.
   CylinderListInfo(int, StyleEnum, double, double, double, int, int, boolean, boolean, int, int)   - 
Constructor for class org.proteinshader.graphics.displaylists. CylinderListInfo 
 Constructs a CylinderListInfo.
   CylinderReferences   - Class in  org.proteinshader.graphics.displaylists  Stores information on multiple OpenGL display lists that can be used
for rendering cylinders with different degrees of detail.   CylinderReferences()   - 
Constructor for class org.proteinshader.graphics.displaylists. CylinderReferences 
 Constructs a CylinderReferences object.
 
 
    
 D  
 
   DATA_DIRECTORY   - 
Static variable in class org.proteinshader.gui.components.menubar. FileMenu 
 Specifies the default directory to read PDB files from.
   DATA_TYPE   - 
Static variable in class org.proteinshader.graphics.typography. GLF2GlyphSetFactory 
 The '.glf' data type is GL.GL_UNSIGNED_BYTE.
   DECIMAL_FORMAT   - 
Static variable in class org.proteinshader.math. Point3d 
 As a convienence for debugging, the toString() method
        will return a representation of a point in the general
        form "(x, y, z)", where the number of decimal places for
        each value will be determined this decimal format String,
        which is "0.000".
   DECIMAL_FORMAT   - 
Static variable in class org.proteinshader.math. Quaternion 
 As a convenience for debugging, the toString() method
        will return a representation of a quaternion in the general
        form "(x, y, z, w)", where the number of decimal places for
        each value will be determined this decimal format String,
        which is "0.000".
   DECIMAL_FORMAT   - 
Static variable in class org.proteinshader.math. Vec3d 
 As a convienence for debugging, the toString() method
        will return a representation of a vector in the general
        form "(x, y, z)", where the number of decimal places for
        each value will be determined this decimal format String,
        which is "0.000".
   DECORATION   - 
Static variable in class org.proteinshader.structure. Segment 
 The default decoration is HALFTONING.
   DecorationEnum   - Enum in  org.proteinshader.structure.enums  Provides an enumeration to specify if a Segment object should be plain,
with text labels, or with halftoning when the Segment is rendered as a
three-dimensional tube or ribbon.   DecorationsPanel   - Class in  org.proteinshader.gui.components.controlpanel  This control panel allows the user to select a decoration type (Plain,
Text Labels, or Halftoning) for Segments of a tube or ribbon.   DecorationsPanel(Mediator, Frame, RadioPanel)   - 
Constructor for class org.proteinshader.gui.components.controlpanel. DecorationsPanel 
 Constructs a DecorationsPanel.
   DecorationsPanelListenerFactory   - Class in  org.proteinshader.gui.listeners.controlpanel  Creates the listeners for the DecorationsPanel.   DecorationsPanelListenerFactory(Mediator, DecorationsPanel)   - 
Constructor for class org.proteinshader.gui.listeners.controlpanel. DecorationsPanelListenerFactory 
 Constructs a DecorationsPanelListenerFactory.
   DEFAULT   - 
Static variable in class org.proteinshader.graphics. Silhouette 
 Determines if translucent black silhouettes are used by default 
        for antialiasing the dark edges of tubes or ribbons drawn with 
        halftoning.
   DEFAULT_ALPHA   - 
Static variable in class org.proteinshader.structure. Drawable 
 The default value for alpha is 0.0.
   DEFAULT_BLUE   - 
Static variable in class org.proteinshader.structure. Drawable 
 The default value for blue is 0.0.
   DEFAULT_CAP_TILING   - 
Static variable in class org.proteinshader.graphics.displaylists. CylinderReferences 
 The default tiling (number of slices and stacks) for
        a capping sphere is 5.
   DEFAULT_CHAIN_ID   - 
Static variable in class org.proteinshader.structure.io. PDBLineParser 
 Default chainID to use if the
        chainID field of a record is blank.
   DEFAULT_EXTENSION   - 
Static variable in class org.proteinshader.gui.utils. FileExtensionFilter 
  
   DEFAULT_FILENAME   - 
Static variable in class org.proteinshader.gui.utils. ImageFileChooser 
 The default filename for saving a file is canvas.
   DEFAULT_GREEN   - 
Static variable in class org.proteinshader.structure. Drawable 
 The default value for green is 0.0.
   DEFAULT_HEIGHT   - 
Static variable in class org.proteinshader.graphics.displaylists. CylinderReferences 
 The default cylinder height is 1.0.
   DEFAULT_JITTER   - 
Static variable in class org.proteinshader.gui.components.controlpanel. AntialiasingPanel 
 Sets the initial menu selection for the number of times to jitter
        when applying antialiasing to the entire scene (n = 3).
   DEFAULT_LONG_FACTOR   - 
Static variable in class org.proteinshader.gui.components. SpringLoadedJFrame 
 Default height for LEFT or RIGHT spring-loaded frame, but
        default width for TOP or BOTTOM spring-loaded frame.
   DEFAULT_MIN_BOND_LENGTH   - 
Static variable in class org.proteinshader.structure.visitor. BondPredictor 
 The default minimum Bond length is 0.40 Angstroms.
   DEFAULT_MODEL_ID   - 
Static variable in class org.proteinshader.structure.io. PDBLineParser 
 Default modelID to use if there is only one unnamed model.
   DEFAULT_RADIUS   - 
Static variable in class org.proteinshader.graphics.displaylists. CylinderReferences 
 Cylinders intended for a Sticks type of display will use
        a default cylinder radius is 0.15.
   DEFAULT_RADIUS   - 
Static variable in class org.proteinshader.graphics.displaylists. SphereReferences 
 The spheres cached by this class will use a default radius
        of 1.0.
   DEFAULT_RADIUS   - 
Static variable in class org.proteinshader.structure. Drawable 
 The default radius is 1.0.
   DEFAULT_RED   - 
Static variable in class org.proteinshader.structure. Drawable 
 The default value for red is 0.0.
   DEFAULT_SHAPE   - 
Static variable in class org.proteinshader.structure. Helix 
 The default Helix shape is ALPHA_RIBBON.
   DEFAULT_SHORT_FACTOR   - 
Static variable in class org.proteinshader.gui.components. SpringLoadedJFrame 
 0.25 is the default width for LEFT or RIGHT
        spring-loaded frame, but the default height
        for TOP or BOTTOM spring-loaded frame.
   DEFAULT_SLICES   - 
Static variable in class org.proteinshader.graphics.displaylists. CylinderReferences 
 The default number of slices for a cylinder is 10.
   DEFAULT_SPECULAR_EXP   - 
Static variable in class org.proteinshader.structure. Drawable 
 The default value for the specular exponent is 20.0.
   DEFAULT_STACKS   - 
Static variable in class org.proteinshader.graphics.displaylists. CylinderReferences 
 The default number of stacks for a cylinder is 1.
   DEFAULT_TILING   - 
Static variable in class org.proteinshader.graphics.displaylists. SphereReferences 
 The default tiling is 12, and is used to set the initial
        number of slices and stacks for a generic sphere for
        SPACE_FILLING style displays and a generic sphere for
        BALLS_AND_STICKS style displays.
   DEFAULT_TOLERANCE   - 
Static variable in class org.proteinshader.structure.visitor. BondPredictor 
 The default tolerance for determining if a
        Bond length is acceptable is 0.56 Angstroms
        (the same value used by the RasMol program).
   DEFAULT_TYPE   - 
Static variable in class org.proteinshader.structure. Helix 
 The default Helix type is RH_ALPHA.
   DELAY   - 
Static variable in class org.proteinshader.gui.components. SpringLoadedJFrame 
 Sets the delay for the timer circuit
        used to open the spring-loaded frame.
   deleteDisplayList(GL, int)   - 
Method in class org.proteinshader.graphics. Shape 
 Deletes an OpenGL display list.
   deleteDisplayLists(GL)   - 
Method in class org.proteinshader.graphics.typography. TextLabelManager 
 Frees graphics card memory for any OpenGL display lists currently
    being used to store text labels.
   deleteShaderProgram(GL, ShaderProgram)   - 
Method in class org.proteinshader.graphics.shader. ShaderProgramFactory 
 Calls delete on each OpenGL shader and program object name held by
    the ShaderProgram given as an argument.
   deleteShaders(GL)   - 
Method in class org.proteinshader.graphics.shader. ShaderManager 
 Frees graphics card memory for all shaders.
   deleteTablaRasa(GL)   - 
Method in class org.proteinshader.graphics.typography. TextLabelManager 
 Frees graphics card memory for the tabla rasa.
   deleteTextures(GL)   - 
Method in class org.proteinshader.graphics.textures. TextureManager 
 Frees graphics card memory for all previously cached textures.
   Description   - Class in  org.proteinshader.structure  A Description object serves as a container for the Category objects
that hold Records describing a PDB structure entry.   Description(String)   - 
Constructor for class org.proteinshader.structure. Description 
 Constructs a Description with the structureID specified as an
    argument.
   DIALOG_HEIGHT   - 
Static variable in class org.proteinshader.gui.components.controlpanel. SelectorPanel 
 The height of the Atoms list dialog box.
   DIALOG_WIDTH   - 
Static variable in class org.proteinshader.gui.components.controlpanel. SelectorPanel 
 The width of the Atoms list dialog box.
   DIFFUSE_LIGHT   - 
Static variable in class org.proteinshader.gui.viewing. Lighting 
 The diffuse light is { 0.95f, 0.95f, 0.95f, 0.0f }.
   disableLighting(GL)   - 
Method in class org.proteinshader.gui.viewing. Lighting 
 Disables the lighting.
   display(GLAutoDrawable)   - 
Method in class org.proteinshader.gui. Renderer 
 Draws the currently selected Model on the canvas.
   displayAlphaCarbon()   - 
Method in class org.proteinshader.structure. Segment 
 Returns true if the alpha-carbon should be displayed.
   displayChanged(GLAutoDrawable, boolean, boolean)   - 
Method in class org.proteinshader.gui. Renderer 
 Called on if the display mode or display device is changed while
    the program is running.
   displayErrorAndExit(String, String)   - 
Method in interface org.proteinshader.gui. Mediator 
 Display an error message in a JOptionPane before exiting.
   displayErrorAndExit(String, String)   - 
Method in class org.proteinshader.gui. MediatorImpl 
 Display an error message in a JOptionPane before exiting.
   displayErrorAndExit(String, String)   - 
Method in class org.proteinshader.gui. ProteinShaderGUI 
 Display an error message in a JOptionPane before exiting.
   displayWarning(String, String)   - 
Method in interface org.proteinshader.gui. Mediator 
 Display a warning message in a JOptionPane.
   displayWarning(String, String)   - 
Method in class org.proteinshader.gui. MediatorImpl 
 Display a warning message in a JOptionPane.
   displayWarning(String, String)   - 
Method in class org.proteinshader.gui. ProteinShaderGUI 
 Display a warning message in a JOptionPane.
   distance(Drawable)   - 
Method in class org.proteinshader.structure. Drawable 
 Returns the distance in angstroms between the xyz-center of the
    calling Drawable object and the xyz-center of the Drawable object
    given as an argument.
   dot(Vec3d)   - 
Method in class org.proteinshader.math. Vec3d 
 Returns the dot product of the calling vector and the vector given
    as an argument.
   draw(GL, Drawable)   - 
Method in class org.proteinshader.graphics.adapter. StructureToGraphics 
 Determines if a Drawable object is an Atom, Bond, or Segment, and
    then calls on the appropriate draw() method.
   draw(GL, Atom)   - 
Method in class org.proteinshader.graphics.adapter. StructureToGraphics 
 Draws an Atom as a sphere.
   draw(GL, Bond)   - 
Method in class org.proteinshader.graphics.adapter. StructureToGraphics 
 Draws the Bond as a single cylinder for a Balls-and-Sticks style
    display, but two cylinders for a Sticks style display.
   draw(GL, Segment)   - 
Method in class org.proteinshader.graphics.adapter. StructureToGraphics 
 Draws a Segment of a Region (GeneralLoop, Helix, or BetaStrand).
   draw(GL, double, double, double, int, int, boolean, boolean, int, int)   - 
Method in class org.proteinshader.graphics. Cylinder 
 Draws a cylinder oriented along the z-axis, with the base of
    the cylinder at the origin and the top of the cylinder at
    xyz = (0, 0, height).
   draw(GL, LocalFrame[])   - 
Method in class org.proteinshader.graphics. ExtrudedShape 
 Uses an array of LocalFrame objects to draw a segment of an
    extruded shape.
   draw(GL, LocalFrame[])   - 
Method in class org.proteinshader.graphics. FrenetFrames 
 Uses an array of LocalFrame objects to draw xyz-axes.
   draw(GL, LocalFrame, double, double, int, float, float, float, float)   - 
Method in class org.proteinshader.graphics. FrenetFrames 
 Draws an xyz-axis based on the rotation and translation in the
    LocalFrame given as an argument.
   draw(GL, LocalFrame[])   - 
Method in class org.proteinshader.graphics. Ribbon 
 Uses an array of LocalFrame objects to draw the broad surfaces of
    of a ribbon.
   draw(GL, double, int, int)   - 
Method in class org.proteinshader.graphics. Sphere 
 Draws a sphere with texture coordinates, normals, and tangents.
   draw(GL, LocalFrame[])   - 
Method in class org.proteinshader.graphics. Tube 
 Uses an array of LocalFrame objects to draw a segment of a tube.
   draw(LocalFrame[])   - 
Method in class org.proteinshader.structure.io. TubeVertices 
 Uses an array of LocalFrame objects to draw a segment of a tube.
   Drawable   - Class in  org.proteinshader.structure  This abstract class defines attributes and methods that are shared by
all drawable objects, including the xyz-coordinates for the Drawable's
center and methods that will allow each object to be sorted based on
distance from the camera.   Drawable(double, double, double, VisibilityEnum, DrawableEnum)   - 
Constructor for class org.proteinshader.structure. Drawable 
 Constructor for use by subclasses.
   Drawable(double, double, double, VisibilityEnum, DrawableEnum, float, float, float, float)   - 
Constructor for class org.proteinshader.structure. Drawable 
 Constructor for use by subclasses.
   DrawableEnum   - Enum in  org.proteinshader.structure.enums  Provides an enumeration to specify if a Drawable object is an
Atom, Bond, or Segment.   DrawableModifier   - Class in  org.proteinshader.structure.visitor.modifiers  This helper class for the ModifierVisitor knows how to accept a
Drawable object and modify its attributes.   DrawableModifier()   - 
Constructor for class org.proteinshader.structure.visitor.modifiers. DrawableModifier 
 Constructs a DrawableModifier
   DrawableSorter   - Class in  org.proteinshader.structure.sort  Knows how to sort an array of Drawables by their distance from the
camera.   DrawableSorter()   - 
Constructor for class org.proteinshader.structure.sort. DrawableSorter 
 Constructs a DrawableSorter.
   drawEndCap(GL, LocalFrame)   - 
Method in class org.proteinshader.graphics. ExtrudedShape 
 Creates a cap for the end of a segment by drawing the waist
    polygon.
   drawEndCap(GL, LocalFrame)   - 
Method in class org.proteinshader.graphics. FrenetFrames 
 Draws the local frame using red for the sphere at the base.
   drawEndCap(GL, LocalFrame)   - 
Method in class org.proteinshader.graphics. Ribbon 
 Creates a cap for the end of a segment by drawing the waist
    polygon.
   drawEndCap(GL, LocalFrame)   - 
Method in class org.proteinshader.graphics. Tube 
 Creates a cap for the end of a segment by drawing the waist
    polygon.
   drawEndCap(GL)   - 
Method in class org.proteinshader.graphics.utils. SegmentRenderer 
 Draws the end cap for the last tube or ribbon segment that was
    drawn.
   drawRibbon(GL, Segment, SegmentListInfo, boolean)   - 
Method in class org.proteinshader.graphics. Silhouette 
 Draws a translucent black silhoutte of a ribbon segment using jitter.
   drawRibbon(GL, Segment)   - 
Method in class org.proteinshader.graphics.utils. SegmentRenderer 
 Draws the broad surfaces of a ribbon segment and remembers info
    needed by any subsequent call to drawThinSidesOfRibbon(), 
    drawStartCap(), or drawEndCap().
   drawStartCap(GL, LocalFrame)   - 
Method in class org.proteinshader.graphics. ExtrudedShape 
 Creates a cap for the beginning of a segment by drawing the
    waist polygon.
   drawStartCap(GL, LocalFrame)   - 
Method in class org.proteinshader.graphics. FrenetFrames 
 Draws the local frame using light blue for the sphere at the base.
   drawStartCap(GL, LocalFrame)   - 
Method in class org.proteinshader.graphics. Ribbon 
 Creates a cap for the beginning of a segment by drawing the
    waist polygon.
   drawStartCap(GL, LocalFrame)   - 
Method in class org.proteinshader.graphics. Tube 
 Creates a cap for the beginning of a segment by drawing the
    waist polygon.
   drawStartCap(GL)   - 
Method in class org.proteinshader.graphics.utils. SegmentRenderer 
 Draws the start cap for the last tube or ribbon segment that was
    drawn.
   drawThinSidesOfRibbon(GL)   - 
Method in class org.proteinshader.graphics.utils. SegmentRenderer 
 Draws the thin sides for the last ribbon segment that was drawn.
   drawThinSideSurfaces(GL, LocalFrame[])   - 
Method in class org.proteinshader.graphics. Ribbon 
 Draws the thin side surfaces for a segment of a ribbon.
   drawTube(GL, Segment, SegmentListInfo, boolean)   - 
Method in class org.proteinshader.graphics. Silhouette 
 Draws a translucent black silhoutte of a tube segment using jitter.
   drawTube(GL, Segment)   - 
Method in class org.proteinshader.graphics.utils. SegmentRenderer 
 Draws the curved body of a tube segment and remembers info needed
    by any subsequent call to drawStartCap() or drawEndCap().
 
 
    
 E  
 
   enableAllCheckBoxes(boolean)   - 
Method in class org.proteinshader.gui.components.menubar. VisibilityMenu 
 Enables or disables all check box menu items.
   enableAminoAcidsCheckBox(boolean)   - 
Method in class org.proteinshader.gui.components.menubar. VisibilityMenu 
 Enables or disables the 'Amino Acids' check box menu item.
   enableFPSShaders(GL, int)   - 
Method in class org.proteinshader.graphics.shader. ShaderManager 
 Calls on glUseProgram() to make the fps (frames per second label)
    shader pair the active shader program.
   enableGrayscaleShaders(GL)   - 
Method in class org.proteinshader.graphics.shader. ShaderManager 
 Calls on glUseProgram() to make the grayscale shader pair the
    active shader program.
   enableHeterogensCheckBox(boolean)   - 
Method in class org.proteinshader.gui.components.menubar. VisibilityMenu 
 Enables or disables the 'Heterogens' check box menu item.
   enableLightOne(GL, float, float, float)   - 
Method in class org.proteinshader.gui.viewing. Lighting 
 Light one is set to the xyz-coordinates given as arguments

      
    Light one is a positional light and is intended to be used for
    specular lighting.
   enableLightZero(GL)   - 
Method in class org.proteinshader.gui.viewing. Lighting 
 Light zero is set to the position given in LIGHT0_POSITIONS.
   enablePatternsShaders(GL, int)   - 
Method in class org.proteinshader.graphics.shader. ShaderManager 
 Calls on glUseProgram() to make the patterns shader pair the
    active shader program.
   enablePhongShaders(GL)   - 
Method in class org.proteinshader.graphics.shader. ShaderManager 
 Calls on glUseProgram() to make the Phong shader pair the active
    shader program.
   enableRibbonHalftoningShaders(GL, int, int, float, boolean, boolean, float, float)   - 
Method in class org.proteinshader.graphics.shader. ShaderManager 
 Calls on glUseProgram() to make the ribbon halftoning shader pair
    the active shader program.
   enableSilhouetteShaders(GL, boolean, boolean)   - 
Method in class org.proteinshader.graphics.shader. ShaderManager 
 Calls on glUseProgram() to make the silhouette shader pair the
    active shader program.
   enableTextLabelShaders(GL)   - 
Method in class org.proteinshader.graphics.shader. ShaderManager 
 Calls on glUseProgram() to make the text-label shader pair the
    active shader program.
   enableTexture(GL, String, int, RegionEnum)   - 
Method in class org.proteinshader.graphics.typography. TextLabelManager 
 Enables OpenGL texture mapping and binds a texture with text
    cooresponding to the segmentID given as an argument.
   enableTubeCapHalftoningShaders(GL, int)   - 
Method in class org.proteinshader.graphics.shader. ShaderManager 
 Calls on glUseProgram() to make the tube cap halftoning shader
    pair the active shader program.
   enableTubeCapWireframeShaders(GL)   - 
Method in class org.proteinshader.graphics.shader. ShaderManager 
 Calls on glUseProgram() to make the tube cap wireframe shader pair
    the active shader program.
   enableTubeHalftoningShaders(GL, int, int, float, boolean, boolean)   - 
Method in class org.proteinshader.graphics.shader. ShaderManager 
 Calls on glUseProgram() to make the tube halftoning shader pair
    the active shader program.
   enableWatersCheckBox(boolean)   - 
Method in class org.proteinshader.gui.components.menubar. VisibilityMenu 
 Enables or disables the 'Waters' check box menu item.
   enableWireframeShaders(GL, float, float, float)   - 
Method in class org.proteinshader.graphics.shader. ShaderManager 
 Calls on glUseProgram() to make the wireframe shader pair the
    active shader program.
   END_CAP_COLOR   - 
Static variable in class org.proteinshader.graphics. ExtrudedShape 
 Segment end (carboxyl end) caps are colored red because
        that is the CPK color for oxygen.
   END_INDEX   - 
Static variable in class org.proteinshader.graphics.typography. GLF2Header 
 The end char integer starts at index 16 of the header.
   EqualSignConfigReader   - Class in  org.proteinshader.graphics.textures  Reads a configuration file to obtain a list of menu names
and matching filenames for textures.   EqualSignConfigReader()   - 
Constructor for class org.proteinshader.graphics.textures. EqualSignConfigReader 
 Constructs an EqualSignConfigReader object.
   executeDisplayList(GL, double, int)   - 
Method in class org.proteinshader.graphics. Shape 
 Executes an OpenGL display list and uniformly scales the geometric
    object.
   executeDisplayList(GL, int)   - 
Method in class org.proteinshader.graphics. Shape 
 Executes an OpenGL display list.
   EXPONENT   - 
Static variable in class org.proteinshader.graphics.displaylists. CylinderReferences 
 The empirically-determined exponent for the power
        equation is -0.5267.
   EXPONENT   - 
Static variable in class org.proteinshader.graphics.displaylists. SphereReferences 
 The empirically-determined exponent for the power
        equation is -0.7367.
   EXTRA_HEIGHT   - 
Static variable in class org.proteinshader.gui.components.menubar. AboutBox 
  
   EXTRA_WIDTH   - 
Static variable in class org.proteinshader.gui.components.menubar. AboutBox 
  
   extractAltLocation(String)   - 
Method in class org.proteinshader.structure.io. PDBAtomFieldExtractor 
 Obtains the alternate location from (altLoc field: 17), or returns
    an empty String if it is not found (this field is usually blank).
   extractAtomID(String)   - 
Method in class org.proteinshader.structure.io. PDBAtomFieldExtractor 
 Obtains the atomID from (name field: 13-16).
   extractAtomSerialNo(String)   - 
Method in class org.proteinshader.structure.io. PDBAtomFieldExtractor 
 Obtains the atom serial number from (serial field: 7-11).
   extractAtomType(String)   - 
Method in class org.proteinshader.structure.io. PDBAtomFieldExtractor 
 Obtains the atom type (chemical element) from an ATOM or HETATM
    record.
   extractBetaStrandNumber(String)   - 
Method in class org.proteinshader.structure.io. PDBBetaStrandFieldExtractor 
 Obtains the betaStrandNumber from (strandNo field: 8-10).
   extractChainID(String)   - 
Method in class org.proteinshader.structure.io. PDBAtomFieldExtractor 
 Obtains the chainID from (chainID field: 22 ) if it is present.
   extractChainID(String)   - 
Method in class org.proteinshader.structure.io. PDBBetaStrandFieldExtractor 
 Obtains the chainID from (initChainID field: 22).
   extractChainID(String)   - 
Method in class org.proteinshader.structure.io. PDBHelixFieldExtractor 
 Obtains the chainID from (initChainID field: 20).
   extractCharge(String)   - 
Method in class org.proteinshader.structure.io. PDBAtomFieldExtractor 
 Obtains the charge from (charge field: 79-80), or sets it to 0 if
    the field is only whitespace.
   extractDstAtomSerialNo(String)   - 
Method in class org.proteinshader.structure.io. PDBConnectFieldExtractor 
 Obtains destination Atom serial number(s) from
     

     field 1 = (serial field: 12-16)
     field 2 = (serial field: 17-21)
     field 3 = (serial field: 22-26)
     field 4 = (serial field: 27-31)

      
    It is legal for any (or even all 4) of these fields to be blank,
    as the CONECT record may be used to specify salt bridges or
    hydrogen bonds that are found in other fields.
   extractEndResidueID(String)   - 
Method in class org.proteinshader.structure.io. PDBBetaStrandFieldExtractor 
 Obtains the endResidueID by combining (endResName field: 29-31),
    (endSeqNum field: 34-37), and the optional (endICode field: 38).
   extractEndResidueID(String)   - 
Method in class org.proteinshader.structure.io. PDBHelixFieldExtractor 
 Obtains the endResidueID by combining (endResName field: 28-30),
    (endSeqNum field: 34-37), and the optional (endICode field: 38).
   extractGlyphImage(byte[][])   - 
Method in class org.proteinshader.graphics.typography. Glyph 
 Extracts the glyph from the texture image given as an argument.
   extractHelixID(String)   - 
Method in class org.proteinshader.structure.io. PDBHelixFieldExtractor 
 Obtains the helixID from (helixID field: 12-14).
   extractOccupancy(String)   - 
Method in class org.proteinshader.structure.io. PDBAtomFieldExtractor 
 Obtains the occupancy from (occupancy field: 55-60).
   extractResidueID(String)   - 
Method in class org.proteinshader.structure.io. PDBAtomFieldExtractor 
 Obtains the residueID by combining (resName field: 18-20),
    (resSeq field: 23-26), and the optional (icode field: 27).
   extractResidueName(String)   - 
Method in class org.proteinshader.structure.io. PDBAtomFieldExtractor 
 Obtains the Residue name from (resName field: 18-20).
   extractSense(String)   - 
Method in class org.proteinshader.structure.io. PDBBetaStrandFieldExtractor 
 Obtains the sense (orientation) from (sense field: 39-40).
   extractSerialNo(String)   - 
Method in class org.proteinshader.structure.io. PDBHelixFieldExtractor 
 Obtains the helix serial number from (serial field: 8-10).
   extractSheetID(String)   - 
Method in class org.proteinshader.structure.io. PDBBetaStrandFieldExtractor 
 Obtains the sheetID from (sheetID field: 12-14).
   extractSrcAtomSerialNo(String)   - 
Method in class org.proteinshader.structure.io. PDBConnectFieldExtractor 
 Obtains the source Atom serial number from (serial field: 7-11)
   extractStartResidueID(String)   - 
Method in class org.proteinshader.structure.io. PDBBetaStrandFieldExtractor 
 Obtains the startResidueID by combining (initResName field:
    18-20), (initSeqNum field: 23-26), and the optional (initICode
    field: 27).
   extractStartResidueID(String)   - 
Method in class org.proteinshader.structure.io. PDBHelixFieldExtractor 
 Obtains the startResidueID by combining (initResName field:
    16-18), (initSeqNum field: 22-25), and the optional (initICode
    field: 26).
   extractStrandsInSheet(String)   - 
Method in class org.proteinshader.structure.io. PDBBetaStrandFieldExtractor 
 Obtains the number of strands in the sheet from (numStrands field:
    15-16).
   extractTemperature(String)   - 
Method in class org.proteinshader.structure.io. PDBAtomFieldExtractor 
 Obtains the temperature from (tempFactor field: 61-66).
   extractType(String)   - 
Method in class org.proteinshader.structure.io. PDBHelixFieldExtractor 
 Extracts the helix type from (helixClass field: 39-40).
   extractX(String)   - 
Method in class org.proteinshader.structure.io. PDBAtomFieldExtractor 
 Obtains the x-coordinate from (x field: 31-38 ).
   extractY(String)   - 
Method in class org.proteinshader.structure.io. PDBAtomFieldExtractor 
 extractY() - obtains the y-coordinate from (y field: 39-46 ).
   extractZ(String)   - 
Method in class org.proteinshader.structure.io. PDBAtomFieldExtractor 
 extractZ() - obtains the z-coordinate from (z field: 47-54 ).
   ExtrudedShape   - Class in  org.proteinshader.graphics  Contains common methods needed by classes that draw segments of a
three-dimensional tube by sweeping a waist polygon along a spline.   ExtrudedShape()   - 
Constructor for class org.proteinshader.graphics. ExtrudedShape 
 Constructor for use by subclasses.
 
 
    
 F  
 
   FileExtensionFilter   - Class in  org.proteinshader.gui.utils  Checks if a filename has an extension.   FileExtensionFilter()   - 
Constructor for class org.proteinshader.gui.utils. FileExtensionFilter 
 Constructs a FileExtensionFilter using the default file extension.
   FileExtensionFilter(String)   - 
Constructor for class org.proteinshader.gui.utils. FileExtensionFilter 
 Constructs a FileExtensionFilter.
   FileMenu   - Class in  org.proteinshader.gui.components.menubar  Provides the File menu for the MainMenuBar.   FileMenu(Mediator)   - 
Constructor for class org.proteinshader.gui.components.menubar. FileMenu 
 Constructs a FileMenu with menu items "File" and "Quit", and an "Export
    Image" submenu for saving PNG and JPEG files.
   FileMenuListenerFactory   - Class in  org.proteinshader.gui.listeners.menubar  Creates all event listeners needed by the FileMenu of the MainMenuBar.   FileMenuListenerFactory(Mediator)   - 
Constructor for class org.proteinshader.gui.listeners.menubar. FileMenuListenerFactory 
 Constructs a FileMenuListenerFactory.
   flush()   - 
Method in class org.proteinshader.structure.visitor. SFWriterVisitor 
 This method forces the writer to immediately write all data in its
    buffer.
   FONT_DIRECTORY   - 
Static variable in class org.proteinshader.graphics.typography. GlyphSetFactoryFactory 
 Font files (glyph bitmaps) and their associated configuration
        file ('.conf' files) must be in the fonts directory, which
        has the relative pathname '/./../fonts/'.
   FONT_DIRECTORY   - 
Static variable in class org.proteinshader.gui.utils. FpsLabel 
 Stores the location of the fonts director.
   FONT_FILE   - 
Static variable in class org.proteinshader.gui.utils. FpsLabel 
 Stores the name of the fps font file.
   FORMAT   - 
Static variable in class org.proteinshader.graphics.typography. GLF2GlyphSetFactory 
 The '.glf' format is GL.GL_LUMINANCE_ALPHA.
   FOVY   - 
Static variable in class org.proteinshader.gui.viewing. Camera 
 The fovy (field of view on y-axis) for the camera is 60 degrees.
   FpsLabel   - Class in  org.proteinshader.gui.utils  Knows how to print a frames per second label (such as 'fps = 120') on
the canvas.   FpsLabel(StructureToGraphics)   - 
Constructor for class org.proteinshader.gui.utils. FpsLabel 
 Constructs an FpsLabel object.
   FRAMES_PER_SEGMENT   - 
Static variable in class org.proteinshader.graphics. FrenetFrames 
 The default number of frames per segment in a
        FRENET_FRAME display is 21.
   FrenetFrameGeneratorVisitor   - Class in  org.proteinshader.structure.visitor  Calculates a discrete Frenet frame for each AminoAcid visited.   FrenetFrameGeneratorVisitor()   - 
Constructor for class org.proteinshader.structure.visitor. FrenetFrameGeneratorVisitor 
 Constructs a FrenetFrameGeneratorVisitor.
   FrenetFrames   - Class in  org.proteinshader.graphics  Provides a visual representation of the LocalFrame objects of a
Segment by drawing an x-axis, a y-axis, and z-axis.   FrenetFrames()   - 
Constructor for class org.proteinshader.graphics. FrenetFrames 
 Constructs a FrenetFrames object.
 
 
    
 G  
 
   generateAxisAndAngle(Vec3d)   - 
Method in class org.proteinshader.math. Quaternion 
 Converts the Quaternion to an axis of rotation and an angle.
   generateLoopRegions()   - 
Method in class org.proteinshader.structure. Structure 
 Generates Loop objects from any AminoAcids that do not already
    belong to a Helix or BetaStrand object.
   generateMatrix(Vec3d, Vec3d, Vec3d)   - 
Method in class org.proteinshader.math. LocalFrame 
 Generates a 3 x 3 rotation matrix (actually 3 column vectors) that
    is equivalent to the rotation quaternion.
   generateMatrix()   - 
Method in class org.proteinshader.math. Quaternion 
 Generates a 4 x 4 rotation matrix that is equivalent to this quaternion,
    but returns it as a linear array of 16 elements so that it can be used
    with Java Bindings for OpenGL.
   generateMatrix(Vec3d, Vec3d, Vec3d)   - 
Method in class org.proteinshader.math. Quaternion 
 Generates a 3 x 3 rotation matrix (actually 3 column vectors) that
    is equivalent to this quaternion, assuming that the quaternion has
    already been normalized (such that x^2 + y^2 + z^2 + w^2 = 1).
   generateStandardBonds()   - 
Method in class org.proteinshader.structure. Structure 
 Generates standard Bonds (AminoAcids and Waters).
   GeometricListInfo   - Class in  org.proteinshader.graphics.displaylists  The concrete subclasses of this abstract class are used to store
information on an OpenGL display list that hold the commands to draw
a geometric object.   GeometricListInfo()   - 
Constructor for class org.proteinshader.graphics.displaylists. GeometricListInfo 
 Constructs a GeometricListInfo.
   GeometricListInfo(int, StyleEnum)   - 
Constructor for class org.proteinshader.graphics.displaylists. GeometricListInfo 
 Constructs a GeometricListInfo.
   getActiveRadioButton()   - 
Method in class org.proteinshader.gui.components.controlpanel. RadioPanel 
 Returns the RadioButtonEnum corresponding to the currently active
    radio button: SELECTED, HELICES, STRANDS, LOOPS, or GLOBAL.
   getAlpha()   - 
Method in class org.proteinshader.structure. Drawable 
 Returns the alpha component of the RGBA color.
   getAlpha()   - 
Method in enum org.proteinshader.structure.enums. AminoAcidEnum 
 Returns the alpha component of the RGBA color on a scale from 0.0
    to 1.0, inclusive.
   getAlpha()   - 
Method in enum org.proteinshader.structure.enums. AtomEnum 
 Returns the alpha component of the RGBA color on a scale from 0.0
    to 1.0, inclusive.
   getAlpha()   - 
Method in enum org.proteinshader.structure.enums. BondEnum 
 Returns the alpha component of the RGBA color on a scale from 0.0
    to 1.0, inclusive.
   getAlpha()   - 
Method in enum org.proteinshader.structure.enums. RegionEnum 
 Returns the alpha component of the RGBA color on a scale from 0.0
    to 1.0, inclusive.
   getAltAtom()   - 
Method in class org.proteinshader.structure. Atom 
 Returns an alternate Atom with a lower occupancy (if it exists).
   getAltLocation()   - 
Method in class org.proteinshader.structure. Atom 
 Returns the alternate location if the occupancy is less than 1.0.
   getAminoAcid(String)   - 
Method in class org.proteinshader.structure. Chain 
 Returns the AminoAcid with the residueID given as an argument.
   getAminoAcid()   - 
Method in class org.proteinshader.structure. Segment 
 Returns the a reference to the AminoAcid that this Segment
    corresponds to.
   getAminoAcidAfterRegion()   - 
Method in class org.proteinshader.structure. Region 
 Returns the AminoAcid after this Region (or null if it does not
    exist).
   getAminoAcidBeforeRegion()   - 
Method in class org.proteinshader.structure. Region 
 Returns the AminoAcid before this Region (or null if it does not
    exist).
   getAminoAcidLabels()   - 
Method in class org.proteinshader.structure. Structure 
 Returns a list of all AminoAcid residueIDs.
   getAminoAcidLabels()   - 
Method in class org.proteinshader.structure.visitor. AminoAcidLabelVisitor 
 Returns the list of non-redundant labels (AminoAcid residueIDs)
    that
   getAminoAcidType()   - 
Method in class org.proteinshader.structure. Atom 
 If the Atom belongs to an AminoAcid, this method will return the
    AminoAcid type.
   getAminoAcidType()   - 
Method in class org.proteinshader.structure. Segment 
 Returns the type of AminoAcid that this Sement represents.
   getAngstromsPerPixel()   - 
Method in class org.proteinshader.structure. Drawable 
 Returns the angstrom per pixel conversion factor, which is expected to be
    set by the Camera class after the object to camera distance has been
    calculated.
   getAtom(String)   - 
Method in class org.proteinshader.structure. Residue 
 Returns the Atom with the atomID given as an argument, or returns
    null if the Atom is not found.
   getAtom(String, String, String, String)   - 
Method in class org.proteinshader.structure. Structure 
 Returns the Atom with the IDs given as arguments.
   getAtom(int)   - 
Method in class org.proteinshader.structure. Structure 
 Returns the Atom with Atom serial number given as an argument (or
    null if not found).
   getAtomicNumber()   - 
Method in enum org.proteinshader.structure.enums. AtomEnum 
 Returns the atomic number.
   getAtomID()   - 
Method in class org.proteinshader.structure. Atom 
 Returns the ID of this Atom.
   getAtomSerialNoCache()   - 
Method in class org.proteinshader.structure. Structure 
 Returns the Atom serial number cache as a list.
   getBadLines()   - 
Method in class org.proteinshader.structure.io. PDBLineParser 
 Returns a list with all bad lines that were found by parseLine().
   getBadLines()   - 
Method in class org.proteinshader.structure.io. PDBStructureReader 
 Returns a list with any bad lines that were found the last time
    readStructure() was called.
   getBadLines()   - 
Method in interface org.proteinshader.structure.io. StructureReader 
 Returns a list with any bad lines that were found the last time
    readStructure() was called.
   getBallRadius()   - 
Method in class org.proteinshader.structure. Atom 
 Returns the ball radius (for use in stick-and-ball type models).
   getBallRadius()   - 
Method in enum org.proteinshader.structure.enums. AtomEnum 
 Returns a ball radius for use in stick-and-ball type models.
   getBallsAndSticksCylinderInfo()   - 
Method in class org.proteinshader.graphics.displaylists. CylinderReferences 
 Returns the CylinderListInfo object that holds the information
    on an OpenGL display list for a cylinder to be used for a
    BALLS_AND_STICKS style display.
   getBallsAndSticksRef()   - 
Method in class org.proteinshader.graphics.displaylists. CylinderReferences 
 Returns the name (an integer) of an OpenGL display list for a
    cylinder intended to be used for Balls-and-Sticks style displays.
   getBallsAndSticksRef(double)   - 
Method in class org.proteinshader.graphics.displaylists. CylinderReferences 
 Returns the name (an integer) of an OpenGL display list for a
    cylinder intended to be used for Balls-and-Sticks displays.
   getBallsAndSticksRef()   - 
Method in class org.proteinshader.graphics.displaylists. SphereReferences 
 Returns the name (an integer) of an OpenGL display list for a
    sphere intended to be used for a Balls-and-Sticks style display.
   getBallsAndSticksRef(double)   - 
Method in class org.proteinshader.graphics.displaylists. SphereReferences 
 Returns the name (an integer) of an OpenGL display list for a
    sphere intended to be used for a Balls-and-Sticks style display.
   getBallsAndSticksSphereInfo()   - 
Method in class org.proteinshader.graphics.displaylists. SphereReferences 
 Returns the SphereListInfo object that holds the information on an
    OpenGL display list for a sphere to be used in a BALLS_AND_STICKS style
    display.
   getBaseRadius()   - 
Method in class org.proteinshader.graphics.displaylists. CylinderListInfo 
 Returns the base radius of the cylinder in the display list.
   getBendFactor()   - 
Method in class org.proteinshader.structure. Segment 
 Returns the bend factor, which is calculated using the tangent
    at the very beginning of the segment and the tangent at the very
    end of the segment.
   getBendTexture()   - 
Method in class org.proteinshader.structure. Segment 
 Returns the name (an integer) of an OpenGL texture object (stored
    on the graphics card) that can be used as a second texture for
    adding extra lines in the middle of segments that have a sharp
    bend to them.
   getBendTextures()   - 
Method in class org.proteinshader.graphics.adapter. StructureToGraphics 
 Returns the bend Texture objects that were created with the
    loadTextures() method and are intended for use with halftoning.
   getBendTextures()   - 
Method in class org.proteinshader.graphics.textures. TextureManager 
 Returns a list of Java Texture objects with information on OpenGL
    texture objects that are currently stored on the graphics card.
   getBendTextures()   - 
Method in interface org.proteinshader.gui. Mediator 
 Returns the list of Textures intended for highlighting segment
    bend regions when halftoning is being used.
   getBendTextures()   - 
Method in class org.proteinshader.gui. MediatorImpl 
 Returns the list of Textures intended for highlighting segment
    bend regions when halftoning is being used.
   getBetaStrand(String)   - 
Method in class org.proteinshader.structure. Chain 
 Returns the BetaStrand with the betaStrandID given as an argument.
   getBetaStrand(String)   - 
Method in class org.proteinshader.structure. Model 
 Returns the BetaStrand with the betaStrandID given as an argument.
   getBetaStrand(String, String)   - 
Method in class org.proteinshader.structure. Structure 
 Returns the BetaStrand with the IDs given as arguments.
   getBetaStrandID()   - 
Method in class org.proteinshader.structure. BetaStrand 
 Returns the BetaStrand ID.
   getBetaStrandNumber()   - 
Method in class org.proteinshader.structure. BetaStrand 
 Returns the BetaStrand number.
   getBetaStrandNumber()   - 
Method in class org.proteinshader.structure.io. BetaStrandRecord 
 Returns the BetaStrand number.
   getBinormal()   - 
Method in class org.proteinshader.math. LocalFrame 
 Converts the rotation (a Quaternion) into a 3 x 3 rotation matrix
    and the returns the second column vector (the Binormal).
   getBinormal()   - 
Method in class org.proteinshader.math. Quaternion 
 Converts the Quaternion into a 3 x 3 rotation matrix and the
    returns the second column vector (the Binormal).
   getBitmapHeight()   - 
Method in class org.proteinshader.graphics.typography. GLF2Header 
 Returns the height of the bitmap.
   getBitmapWidth()   - 
Method in class org.proteinshader.graphics.typography. GLF2Header 
 Returns the width of the bitmap.
   getBlue()   - 
Method in class org.proteinshader.structure. Drawable 
 Returns the blue component of the RGBA color.
   getBlue()   - 
Method in enum org.proteinshader.structure.enums. AAColorEnum 
 Returns the blue component of the RGBA color as a float in the
    range from 0.0 to 1.0, inclusive.
   getBlue()   - 
Method in enum org.proteinshader.structure.enums. AminoAcidEnum 
 Returns the blue component of the RGBA color on a scale from 0.0
    to 1.0, inclusive.
   getBlue()   - 
Method in enum org.proteinshader.structure.enums. AtomEnum 
 Returns the blue component of the RGBA color on a scale from 0.0
    to 1.0, inclusive.
   getBlue()   - 
Method in enum org.proteinshader.structure.enums. BondEnum 
 Returns the blue component of the RGBA color on a scale from 0.0
    to 1.0, inclusive.
   getBlue()   - 
Method in enum org.proteinshader.structure.enums. CPKColorEnum 
 Returns the blue component of the RGBA color as a float in the
    range from 0.0 to 1.0, inclusive.
   getBlue()   - 
Method in enum org.proteinshader.structure.enums. RegionColorEnum 
 Returns the blue component of the RGBA color as a float in the
    range from 0.0 to 1.0, inclusive.
   getBlue()   - 
Method in enum org.proteinshader.structure.enums. RegionEnum 
 Returns the blue component of the RGBA color on a scale from 0.0
    to 1.0, inclusive.
   getBond(Atom)   - 
Method in class org.proteinshader.structure. Atom 
 Returns the Bond from this Atom (the source) to the destination
    Atom.
   getBytesPerPixel()   - 
Method in exception org.proteinshader.graphics.exceptions. GlyphImageException 
 Returns the number of bytes per pixel for the glyph.
   getBytesPerPixel()   - 
Method in class org.proteinshader.graphics.typography. Glyph 
 Returns the number of bytes per pixel, which should be in the
    range of 1 to 4.
   getBytesPerPixel()   - 
Method in class org.proteinshader.graphics.typography. GlyphSet 
 Returns the number of bytes per pixel.
   getBytesPerPixel()   - 
Method in class org.proteinshader.graphics.typography. TextLabel 
 Returns the number of bytes per pixel.
   getC()   - 
Method in class org.proteinshader.structure. AminoAcid 
 Returns the carbon Atom of the carbonyl group if it exists.
   getCA()   - 
Method in class org.proteinshader.structure. AminoAcid 
 Returns the alpha carbon Atom if it exists.
   getCalculatedLength()   - 
Method in exception org.proteinshader.structure.exceptions. BondLengthException 
 Returns the calculated Bond length in angstroms.
   getCameraDepth()   - 
Method in class org.proteinshader.structure. Drawable 
 Returns the camera depth (z-coordinate in camera space).
   getCameraDistance()   - 
Method in class org.proteinshader.structure. Drawable 
 Returns the camera distance (based on xyz-coordinates in camera space).
   getCapSlices()   - 
Method in class org.proteinshader.graphics.displaylists. CylinderListInfo 
 Returns the number of slices that were used to draw the capping
    sphere at one or both ends of the cylinder (if a capping sphere
    was used).
   getCapStacks()   - 
Method in class org.proteinshader.graphics.displaylists. CylinderListInfo 
 Returns the number of stacks that were used to draw the capping
    sphere at one or both ends of the cylinder (if a capping sphere
    was used).
   getCategory(String)   - 
Method in class org.proteinshader.structure. Description 
 Retrieves a Category by its name.
   getCategory()   - 
Method in enum org.proteinshader.structure.io.enums. RecordEnum 
 Returns a category for a PDB record.
   getCategoryEnum()   - 
Method in enum org.proteinshader.structure.io.enums. RecordEnum 
 Returns a category enum for a PDB record.
   getCategoryName()   - 
Method in class org.proteinshader.structure. Record 
 Returns the name of the Category the Record belongs to.
   getCenter()   - 
Method in class org.proteinshader.structure. AminoAcid 
 Returns a new Point3d object based on the xyz-coordinates of the
    alpha-carbon.
   getChain(String)   - 
Method in class org.proteinshader.structure. Model 
 Returns the Chain with the chainID given as an argument.
   getChain(String, String)   - 
Method in class org.proteinshader.structure. Structure 
 Returns the Chain with the IDs given as arguments.
   getChainID()   - 
Method in class org.proteinshader.structure. Atom 
 Returns the ID of the Chain that this Atom belongs to.
   getChainID()   - 
Method in class org.proteinshader.structure. Chain 
 Returns the chainID, which is usually a single letter.
   getChainID()   - 
Method in class org.proteinshader.structure.io. BetaStrandRecord 
 Returns the Chain ID.
   getChainID()   - 
Method in class org.proteinshader.structure.io. HelixRecord 
 Returns the Chain ID.
   getChainID()   - 
Method in class org.proteinshader.structure. Region 
 Returns the ID of the Chain that this Region belongs to.
   getChainID()   - 
Method in class org.proteinshader.structure. Residue 
 Returns the ID of the Chain that this Residue belongs to.
   getCharge()   - 
Method in class org.proteinshader.structure. Atom 
 Returns the electrical charge on the Atom.
   getColor()   - 
Method in class org.proteinshader.structure. Drawable 
 Returns a copy of the RGBA color as an array.
   getConfigFilename()   - 
Method in exception org.proteinshader.graphics.exceptions. GlyphConfigException 
 Returns the name of the configuation file that had a problem.
   getCovalentRadius()   - 
Method in enum org.proteinshader.structure.enums. AtomEnum 
 Returns the covalent radius for the Atom, which should be useful
    for predicting Bonds with other Atoms.
   getCurrentAtoms()   - 
Method in interface org.proteinshader.gui. Mediator 
 Returns the current Atoms.
   getCurrentAtoms()   - 
Method in class org.proteinshader.gui. MediatorImpl 
 Returns the current Atoms.
   getCurrentChain()   - 
Method in interface org.proteinshader.gui. Mediator 
 Returns the current Chain.
   getCurrentChain()   - 
Method in class org.proteinshader.gui. MediatorImpl 
 Returns the current Chain.
   getCurrentModel()   - 
Method in interface org.proteinshader.gui. Mediator 
 Returns the current Model.
   getCurrentModel()   - 
Method in class org.proteinshader.gui. MediatorImpl 
 Returns the current Model.
   getCurrentResidues()   - 
Method in interface org.proteinshader.gui. Mediator 
 Returns the current Residues.
   getCurrentResidues()   - 
Method in class org.proteinshader.gui. MediatorImpl 
 Returns the current Residues.
   getCylinderInfo(StyleEnum)   - 
Method in class org.proteinshader.graphics.adapter. StructureToGraphics 
 Returns the CylinderListInfo object that holds the information on
    an OpenGL display list for a cylinder that is currently used for
    drawing Bonds.
   getCylinderInfo(StyleEnum)   - 
Method in interface org.proteinshader.gui. Mediator 
 Returns the CylinderListInfo object that holds the information on
    an OpenGL display list for a cylinder that is currently used for
    drawing Bonds.
   getCylinderInfo(StyleEnum)   - 
Method in class org.proteinshader.gui. MediatorImpl 
 Returns the CylinderListInfo object that holds the information on
    an OpenGL display list for a cylinder that is currently used for
    drawing Bonds.
   getDataType()   - 
Method in class org.proteinshader.graphics.typography. GlyphSet 
 Returns the OpenGL data type (such as GL.GL_UNSIGNED_BYTE).
   getDataType()   - 
Method in class org.proteinshader.graphics.typography. TextLabel 
 Returns the OpenGL data type (such as GL_UNSIGNED_BYTE).
   getDecoration()   - 
Method in class org.proteinshader.structure. Segment 
 Returns the decoration type for this Segment (PLAIN, WIREFRAME,
    TEXT_LABEL, PATTERNS, or HALFTONING).
   getDefaultSideChainType()   - 
Static method in enum org.proteinshader.structure.enums. SideChainEnum 
 Returns the default side chain type, which is NONE.
   getDefaultStyle()   - 
Static method in enum org.proteinshader.gui.enums. StyleEnum 
 Returns the default style, which is BALLS_AND_STICKS.
   getDepth()   - 
Method in class org.proteinshader.structure. Model 
 Returns the depth of the Model (maxZ - minZ).
   getDescription()   - 
Method in class org.proteinshader.gui.utils. FileExtensionFilter 
 Returns a description of the supported file type.
   getDescription()   - 
Method in class org.proteinshader.structure.io.filters. PDBFileFilter 
 Constructs a PDBFileFilter.
   getDescription()   - 
Method in class org.proteinshader.structure. Structure 
 Returns a reference to the Description object owned by this
    Structure.
   getDirX()   - 
Method in class org.proteinshader.structure. Bond 
 Returns the x-coordinate of the source to destination Atom
    direction vector.
   getDirY()   - 
Method in class org.proteinshader.structure. Bond 
 Returns the y-coordinate of the source to destination Atom
    direction vector.
   getDirZ()   - 
Method in class org.proteinshader.structure. Bond 
 Returns the z-coordinate of the source to destination Atom
    direction vector.
   getDisplayListName()   - 
Method in class org.proteinshader.graphics.displaylists. GeometricListInfo 
 Returns the name of the OpenGL display list that this GeometricListInfo 
    object stores information on.
   getDrawableType()   - 
Method in class org.proteinshader.structure. Drawable 
 Returns the Drawable type (ATOM, BOND, HELIX, or BETA_STRAND).
   getDstAtom()   - 
Method in class org.proteinshader.structure. Bond 
 Returns the destination Atom for this Bond.
   getDstAtomSerialNo()   - 
Method in exception org.proteinshader.structure.exceptions. BondLengthException 
 Returns the destination Atom PDB serial number.
   getDstAtomType()   - 
Method in exception org.proteinshader.structure.exceptions. BondLengthException 
 Returns the destination Atom type as an AtomEnum.
   getDstDirX()   - 
Method in class org.proteinshader.structure. Bond 
 Returns the x-coordinate of the center to destination Atom
    direction vector.
   getDstDirY()   - 
Method in class org.proteinshader.structure. Bond 
 Returns the y-coordinate of the center to destination Atom
    direction vector.
   getDstDirZ()   - 
Method in class org.proteinshader.structure. Bond 
 Returns the z-coordinate of the center to destination Atom
    direction vector.
   getEndCapName()   - 
Method in class org.proteinshader.graphics.displaylists. SegmentListInfo 
 Returns a reference to the OpenGL display list for the end cap.
   getEndChar()   - 
Method in class org.proteinshader.graphics.typography. GLF2Header 
 Returns the end char (the last character in the glyph set).
   getEndResidueID()   - 
Method in class org.proteinshader.structure.io. BetaStrandRecord 
 Returns the end Residue ID for the BetaStrand.
   getEndResidueID()   - 
Method in class org.proteinshader.structure.io. HelixRecord 
 Returns the end Residue ID for the Helix.
   getEndResidueID()   - 
Method in class org.proteinshader.structure. Region 
 Returns the ID of the last Residue in the sequence.
   getEndRotation()   - 
Method in class org.proteinshader.structure. Segment 
 Returns a clone of the Quaternion for the end rotation of this
    Segment.
   getErrors()   - 
Method in class org.proteinshader.graphics.shader. ShaderProgramFactory 
 Returns a list with all ShaderExceptions that occurred during the last call
    to compileAllShaders().
   getErrors()   - 
Method in class org.proteinshader.graphics.textures. TextureFactory 
 Returns a list with any exceptions that occurred the last time
    that createTextures() was called.
   getErrors()   - 
Method in class org.proteinshader.structure.factory. SegmentFactory 
 Returns a list with any SegmentExceptions that were created
    because an error prevented a Segment from being created.
   getExpectedLength()   - 
Method in exception org.proteinshader.structure.exceptions. BondLengthException 
 Returns the expected Bond length in angstroms.
   getExtension()   - 
Method in enum org.proteinshader.gui.enums. ImageFormatEnum 
 Returns the file extension for this image format.
   getFile()   - 
Method in class org.proteinshader.graphics.textures. Texture 
 Returns a File object with the complete pathname for the file the
    texture was read from.
   getFile()   - 
Method in exception org.proteinshader.gui.exceptions. ScreenShotException 
 Returns the file object that holds the name and path of the file
    to write the screen shot to.
   getFilename()   - 
Method in class org.proteinshader.graphics.textures. NamePair 
 Returns the name of the file the texture is to be read from.
   getFilename()   - 
Method in class org.proteinshader.graphics.textures. Texture 
 Returns the name of the file the texture was read from.
   getFilename()   - 
Method in class org.proteinshader.graphics.typography. GLF2Header 
 Returns the name of the '.glf' file the header is taken from.
   getFilename()   - 
Method in class org.proteinshader.graphics.typography. GlyphSet 
 Returns the name of the file the glyphs were read from.
   getFilenameExtension(File)   - 
Method in class org.proteinshader.gui.utils. FileExtensionFilter 
 Obtains the filename extension (the dot plus the letters after the dot) if
    it exists and returns it as the dot followed by lowercase letters.
   getFilenameExtension(File)   - 
Method in class org.proteinshader.structure.io.filters. PDBFileFilter 
 Obtains the filename extension (the letters after last '.') if it
    exists and return it as lowercase letters.
   getFilenameOrDie(String[], int)   - 
Method in class org.proteinshader.structure.io.demos. PDBStructureReaderDemo 
 Returns the requested command line argument if it is present.
   getFilenameOrDie(String[], int)   - 
Method in class org.proteinshader.structure.io.demos. SegmentDemo 
 Returns the requested command line argument if it is present.
   getFilenameOrDie(String[], int)   - 
Method in class org.proteinshader.structure.visitor.demos. ModifierVisitorDemo 
 Returns the requested command line argument if it is present.
   getFontname()   - 
Method in class org.proteinshader.graphics.typography. GlyphSet 
 Returns the name of the font this set of glyphs belongs to.
   getFontSize()   - 
Method in class org.proteinshader.graphics.typography. GlyphSet 
 Returns the font size.
   getFormat()   - 
Method in class org.proteinshader.graphics.typography. GlyphSet 
 Returns the OpenGL format (such as GL.GL_LUMINANCE_ALPHA).
   getFormat()   - 
Method in class org.proteinshader.graphics.typography. TextLabel 
 Returns the number of bytes per pixel.
   getFormat()   - 
Method in exception org.proteinshader.gui.exceptions. ScreenShotException 
 Returns the format type for the image file that could not be written.
   getFragFile()   - 
Method in enum org.proteinshader.graphics.enums. ShaderEnum 
 Returns the name of the file the fragment shader is stored in.
   getFragFile()   - 
Method in class org.proteinshader.graphics.shader. ShaderProgram 
 Returns the File the fragment shader source code was obtained
    from.
   getFragShaderName()   - 
Method in class org.proteinshader.graphics.shader. ShaderProgram 
 Returns the name (an integer) of the fragment shader object.
   getFrame()   - 
Method in interface org.proteinshader.gui. Mediator 
 Returns the frame that should be used as a parent frame for
    Dialogs.
   getFrame()   - 
Method in class org.proteinshader.gui. MediatorImpl 
 Returns the frame that should be used as a parent frame for
    Dialogs.
   getFullName()   - 
Method in enum org.proteinshader.structure.enums. AminoAcidEnum 
 Returns the full name of the amino acid.
   getFullName()   - 
Method in enum org.proteinshader.structure.io.enums. RecordEnum 
 Returns the full name of the record type as a String.
   getFullName()   - 
Method in class org.proteinshader.structure. Segment 
 Returns the full name of the Segment, which is a concatenation of
    the structureID, modelID, regionID, and residueID (with a single
    blank space between IDs).
   getGlyph(String)   - 
Method in class org.proteinshader.graphics.typography. GlyphSet 
 Returns the Glyph object for the name given as an argument (or
    null if it cannot be found).
   getGlyphHeight()   - 
Method in exception org.proteinshader.graphics.exceptions. GlyphImageException 
 Returns the height of the Glyph in pixels.
   getGlyphs(String)   - 
Method in class org.proteinshader.graphics.typography. GlyphSet 
 Returns a list of Glyph objects corresponding to the characters
    of the String given as an argument.
   getGlyphSetImageHeight()   - 
Method in exception org.proteinshader.graphics.exceptions. GlyphImageException 
 Returns the height in pixels of the glyph set image.
   getGlyphSetImageWidth()   - 
Method in exception org.proteinshader.graphics.exceptions. GlyphImageException 
 Returns the width in pixels of the glyph set image.
   getGlyphWidth()   - 
Method in exception org.proteinshader.graphics.exceptions. GlyphImageException 
 Returns the width of the Glyph in pixels.
   getGraphics()   - 
Method in class org.proteinshader.gui. Renderer 
 Returns a reference to the StructureToGraphics object used by the
    Renderer.
   getGraphicsCardInfo()   - 
Method in interface org.proteinshader.gui. Mediator 
 Returns information on the graphics card that was gathered when the init()
    method of the Renderer was called.
   getGraphicsCardInfo()   - 
Method in class org.proteinshader.gui. MediatorImpl 
 Returns information on the graphics card that was gathered when the init()
    method of the Renderer was called.
   getGraphicsCardInfo()   - 
Method in class org.proteinshader.gui. Renderer 
 Returns information on the graphics card that was gathered when the init()
    method was called.
   getGreen()   - 
Method in class org.proteinshader.structure. Drawable 
 Returns the green component of the RGBA color.
   getGreen()   - 
Method in enum org.proteinshader.structure.enums. AAColorEnum 
 Returns the green component of the RGBA color as a float in the
    range from 0.0 to 1.0, inclusive.
   getGreen()   - 
Method in enum org.proteinshader.structure.enums. AminoAcidEnum 
 Returns the green component of the RGBA color on a scale from 0.0
    to 1.0, inclusive.
   getGreen()   - 
Method in enum org.proteinshader.structure.enums. AtomEnum 
 Returns the green component of the RGBA color on a scale from 0.0
    to 1.0, inclusive.
   getGreen()   - 
Method in enum org.proteinshader.structure.enums. BondEnum 
 Returns the green component of the RGBA color on a scale from 0.0
    to 1.0, inclusive.
   getGreen()   - 
Method in enum org.proteinshader.structure.enums. CPKColorEnum 
 Returns the green component of the RGBA color as a float in the
    range from 0.0 to 1.0, inclusive.
   getGreen()   - 
Method in enum org.proteinshader.structure.enums. RegionColorEnum 
 Returns the green component of the RGBA color as a float in the
    range from 0.0 to 1.0, inclusive.
   getGreen()   - 
Method in enum org.proteinshader.structure.enums. RegionEnum 
 Returns the green component of the RGBA color on a scale from 0.0
    to 1.0, inclusive.
   getHalfLength()   - 
Method in class org.proteinshader.structure. Bond 
 Returns the half-length of the Bond in angstroms.
   getHalftoningTexture()   - 
Method in class org.proteinshader.structure. Segment 
 Returns the name (an integer) of an OpenGL texure object that can
    be used for real-time halftoning (or returns zero if no such
    texture object is associated with this Segment).
   getHalftoningTextures()   - 
Method in class org.proteinshader.graphics.adapter. StructureToGraphics 
 Returns the Texture objects that were created with the
    loadTextures() method and are intended for use with halftoning.
   getHalftoningTextures()   - 
Method in class org.proteinshader.graphics.textures. TextureManager 
 Returns a list of Java Texture objects with information on OpenGL
    texture objects that are currently stored on the graphics card.
   getHalftoningTextures()   - 
Method in interface org.proteinshader.gui. Mediator 
 Returns the list of Texture objects intended for halftoning.
   getHalftoningTextures()   - 
Method in class org.proteinshader.gui. MediatorImpl 
 Returns the list of Texture objects intended for halftoning.
   getHAor1HA()   - 
Method in class org.proteinshader.structure. AminoAcid 
 Returns the hydrogen Atom on the alpha carbon if it exists.
   getHeight()   - 
Method in class org.proteinshader.graphics.displaylists. CylinderListInfo 
 Returns the height of the cylinder stored in the display list.
   getHeight()   - 
Method in class org.proteinshader.graphics.typography. Glyph 
 Returns the height of the glyph in pixels.
   getHeight()   - 
Method in class org.proteinshader.graphics.typography. TextLabel 
 Returns the height of the glyph in pixels.
   getHeight()   - 
Method in class org.proteinshader.structure. Model 
 Returns the height of the Model (maxY - minY).
   getHelix(String)   - 
Method in class org.proteinshader.structure. Chain 
 Returns the Helix with the helixID given as an argument.
   getHelix(String)   - 
Method in class org.proteinshader.structure. Model 
 Returns the Helix with the helixID given as an argument.
   getHelix(String, String)   - 
Method in class org.proteinshader.structure. Structure 
 Returns the Helix with the IDs given as arguments.
   getHelixID()   - 
Method in class org.proteinshader.structure. Helix 
 Returns the Helix ID.
   getHelixID()   - 
Method in class org.proteinshader.structure.io. HelixRecord 
 Returns the Helix ID.
   getHelpMenu()   - 
Method in class org.proteinshader.gui.components.menubar. MainMenuBar 
 Returns the HelpMenu.
   getHermite1()   - 
Method in class org.proteinshader.structure. Segment 
 Creates a clone of the hermite1 object and returns it.
   getHermite2()   - 
Method in class org.proteinshader.structure. Segment 
 Creates a clone of the hermite2 object and returns it.
   getHeterogen(String)   - 
Method in class org.proteinshader.structure. Chain 
 Returns the Heterogen with the residueID given as an argument.
   getHorHN()   - 
Method in class org.proteinshader.structure. AminoAcid 
 Returns the hydrogen Atom on the nitrogen of the amino group.
   getImage()   - 
Method in class org.proteinshader.graphics.typography. Glyph 
 Returns the 2D byte array with the glyph image in it.
   getImage()   - 
Method in class org.proteinshader.graphics.typography. TextLabel 
 Returns the buffer with the text image in it.
   getLength()   - 
Method in class org.proteinshader.structure. Bond 
 Returns the length of the Bond in angstroms.
   getListCellRendererComponent(JList, Object, int, boolean, boolean)   - 
Method in class org.proteinshader.gui.utils. PaddedListCellRenderer 
 Adds a single blank space (" ") in front of value.toString().
   getListCellRendererComponent(JList, Object, int, boolean, boolean)   - 
Method in class org.proteinshader.gui.utils. SeparatorListCellRenderer 
 Adds a separator (a dividing line) to the top of any menu item
    with a String that matches one of the Strings given the
    constuctor.
   getLocalFrame(double)   - 
Method in class org.proteinshader.structure. Segment 
 Creates a LocalFrame along the Segment spline by using Hermite
    interpolation and SLERP (Spherical Linear intERPolation).
   getLocalFrames(int)   - 
Method in class org.proteinshader.structure. Segment 
 Uses SLERP (Spherical Linear intERPolation) and Hermite
    interpolation to generate the requested number of LocalFrames
    from the start frame to the end frame, inclusive.
   getLoop(String)   - 
Method in class org.proteinshader.structure. Model 
 Returns the Loop with the loopID given as an argument.
   getLoop(String, String)   - 
Method in class org.proteinshader.structure. Structure 
 Returns the Loop with the IDs given as arguments.
   getLoopID()   - 
Method in class org.proteinshader.structure. Loop 
 Returns the Loop ID.
   getLowerLevelMessage()   - 
Method in exception org.proteinshader.gui.exceptions. ScreenShotException 
 Returns the message (if any) that was obtained from a lower-level
    exception (for example, from an IOException or an
    IllegalArgumentException).
   getMatrix()   - 
Method in enum org.proteinshader.gui.enums. JitterEnum 
 Returns a two-dimensional matrix where each row has an xy-coordinate pair
    that can be used to jitter a scene.
   getMaxDimension()   - 
Method in class org.proteinshader.structure. Model 
 Returns the maximum dimension (the greatest of width, height, or
    depth).
   getMaxX()   - 
Method in class org.proteinshader.structure. Model 
 Returns the maximum x-coordinate value.
   getMaxY()   - 
Method in class org.proteinshader.structure. Model 
 Returns the maximum y-coordinate value.
   getMaxZ()   - 
Method in class org.proteinshader.structure. Model 
 Returns the maximum z-coordinate value.
   getMenuName()   - 
Method in class org.proteinshader.graphics.textures. NamePair 
 Returns a name suitable for use in a menu.
   getMenuName()   - 
Method in class org.proteinshader.graphics.textures. Texture 
 Returns a name suitable for use in a menu.
   getMenuName()   - 
Method in enum org.proteinshader.gui.enums. ImageFormatEnum 
 Returns the name of the ImageFormatEnum in a form suitable for use in
    a menu.
   getMenuName()   - 
Method in enum org.proteinshader.gui.enums. OrientationEnum 
 Returns the name of the OrientationEnum in a form suitable for use in
    a menu.
   getMenuName()   - 
Method in enum org.proteinshader.gui.enums. StyleEnum 
 Returns the name of the StyleEnum in a form suitable for use in
    a menu.
   getMenuName()   - 
Method in enum org.proteinshader.structure.enums. RegionEnum 
 Returns the menu name for the Region.
   getMenuName()   - 
Method in enum org.proteinshader.structure.enums. SideChainEnum 
 Returns the name of the SideChainEnum in a form suitable for use
    in a menu.
   getMenuName()   - 
Method in enum org.proteinshader.structure.visitor.enums. AAPortionMode 
 Returns the name of the AAPortionMode in a form suitable for use
    in a menu.
   getMiddleBinormal()   - 
Method in class org.proteinshader.structure. Segment 
 Converts the middle rotation (a Quaternion) into a 3 x 3 rotation
    matrix and then returns the third column vector, the Binormal.
   getMiddleNormal()   - 
Method in class org.proteinshader.structure. Segment 
 Converts the middle rotation (a Quaternion) into a 3 x 3 rotation
    matrix and then returns the first column vector, the Normal.
   getMiddleRotation()   - 
Method in class org.proteinshader.structure. Segment 
 Returns a clone of the Quaternion for the middle rotation of this
    Segment.
   getMiddleTangent()   - 
Method in class org.proteinshader.structure. Segment 
 Converts the middle rotation (a Quaternion) into a 3 x 3 rotation
    matrix and then returns the third column vector, the Tangent.
   getMiddleXYZ()   - 
Method in class org.proteinshader.structure. Segment 
 Copies the xyz-coordinates for the center of this Segment into a
    new Vec3d and returns it.
   getMinBondLength()   - 
Method in class org.proteinshader.structure.visitor. BondPredictor 
 Returns the minimum acceptable Bond length that is currently in
    use.
   getMinX()   - 
Method in class org.proteinshader.structure. Model 
 Returns the minimum x-coordinate value.
   getMinY()   - 
Method in class org.proteinshader.structure. Model 
 Returns the minimum y-coordinate value.
   getMinZ()   - 
Method in class org.proteinshader.structure. Model 
 Returns the minimum z-coordinate value.
   getModel(String)   - 
Method in class org.proteinshader.structure. Structure 
 Returns the Model with the modelID given as an argument.
   getModelID()   - 
Method in class org.proteinshader.structure. Atom 
 Returns the ID of the Model that this Atom belongs to.
   getModelID()   - 
Method in class org.proteinshader.structure. Chain 
 Returns the modelID of the Model this Chain belongs to.
   getModelID()   - 
Method in class org.proteinshader.structure. Model 
 Returns the modelID, which is the Model's number.
   getModelID()   - 
Method in class org.proteinshader.structure. Region 
 Returns the ID of the Model that this Region belongs to.
   getModelID()   - 
Method in class org.proteinshader.structure. Residue 
 Returns the ID of the Model that this Residue belongs to.
   getN()   - 
Method in class org.proteinshader.structure. AminoAcid 
 Returns the nitrogen Atom of the amino group if it exists.
   getName()   - 
Method in exception org.proteinshader.graphics.exceptions. GlyphImageException 
 Returns the name of the glyph.
   getName()   - 
Method in class org.proteinshader.graphics.textures. Texture 
 Gets the OpenGL reference (an integer) for a texture object stored
    on the graphics card.
   getName()   - 
Method in class org.proteinshader.graphics.typography. Glyph 
 Returns the name of the glyph.
   getName()   - 
Method in class org.proteinshader.graphics.typography. TextLabel 
 Returns the name of the glyph.
   getName()   - 
Method in class org.proteinshader.structure. Category 
 Returns the name of the Category.
   getName()   - 
Method in enum org.proteinshader.structure.enums. AtomEnum 
 Returns the name of the atom.
   getName()   - 
Method in class org.proteinshader.structure. Heterogen 
 Returns the Heterogen's name.
   getName()   - 
Method in enum org.proteinshader.structure.io.enums. CategoryEnum 
 Returns the name of the category as a String suitable for use in a
    menu.
   getName()   - 
Method in class org.proteinshader.structure. Record 
 Returns the name of the Record.
   getNextSegment()   - 
Method in class org.proteinshader.structure. Segment 
 Returns a reference to the next Segment with the same region so that
    the visiblity status of the next  Segment can be checked before deciding
    to render a start cap for the current tube or ribbon segment.
   getNormal()   - 
Method in class org.proteinshader.math. LocalFrame 
 Converts the rotation (a Quaternion) into a 3 x 3 rotation matrix
    and the returns the first column vector (the Normal).
   getNormal()   - 
Method in class org.proteinshader.math. Quaternion 
 Converts the Quaternion into a 3 x 3 rotation matrix and the
    returns the first column vector (the Normal).
   getO()   - 
Method in class org.proteinshader.structure. AminoAcid 
 Returns the oxygen Atom of the carbonyl group if it exists.
   getO()   - 
Method in class org.proteinshader.structure. Water 
 Returns the oxygen Atom (or null if not found).
   getOccupancy()   - 
Method in class org.proteinshader.structure. Atom 
 Returns the occupancy, which will be less than 1.0 only if the
    atom occupies more than one position in a crystal.
   getOneLetterCode()   - 
Method in enum org.proteinshader.structure.enums. AminoAcidEnum 
 Returns the one-letter code for the amino acid.
   getOpaqueAtomsAndBonds()   - 
Method in class org.proteinshader.structure. Model 
 Copies the list of opaque Atoms and Bonds into an array and returns the
    array.
   getOpaqueSegments()   - 
Method in class org.proteinshader.structure. Model 
 Copies the list of opaque Segments into an array and returns the array.
   getOXT()   - 
Method in class org.proteinshader.structure. AminoAcid 
 Returns the Chain terminating oxygen if it exists.
   getPair()   - 
Method in class org.proteinshader.graphics.shader. ShaderProgram 
 Returns the type of shader pair (vertex and fragment pair).
   getPatternsTexture()   - 
Method in class org.proteinshader.structure. Segment 
 Returns the name (an integer) of an OpenGL texure object that can
    be used for applying a pattern onto the surface of this Segment
    (or returns zero if there is no texture object for applying a
    pattern).
   getPatternsTextures()   - 
Method in class org.proteinshader.graphics.adapter. StructureToGraphics 
 Returns the Texture objects that were created with the
    loadTextures() method and are intended for use as patterns to
    apply to tubes and ribbons drawn in color (rather than
    halftoning).
   getPatternsTextures()   - 
Method in class org.proteinshader.graphics.textures. TextureManager 
 Returns a list of Java Texture objects with information on OpenGL
    texture objects that are currently stored on the graphics card.
   getPatternsTextures()   - 
Method in interface org.proteinshader.gui. Mediator 
 Returns the list of Texture objects intended for placing patterns
    on colored surfaces (rather than textures for halftoning).
   getPatternsTextures()   - 
Method in class org.proteinshader.gui. MediatorImpl 
 Returns the list of Texture objects intended for placing patterns
    on colored surfaces (rather than textures for halftoning).
   getPDBClassNo()   - 
Method in enum org.proteinshader.structure.enums. HelixEnum 
 Returns the PDB classification number.
   getPercentField()   - 
Method in class org.proteinshader.gui.components.controlpanel. VisibilityPanel 
 Returns the number entered in the "% Translucent" text field.
   getPoint()   - 
Method in class org.proteinshader.structure. Drawable 
 Returns the xyz-center of the Drawable as a point.
   getPrevSegment()   - 
Method in class org.proteinshader.structure. Segment 
 Returns a reference to the previous Segment with the same region so that
    the visiblity status of the previous Segment can be checked before 
    deciding to render a start cap for the current tube or ribbon segment.
   getProgramName()   - 
Method in class org.proteinshader.graphics.shader. ShaderProgram 
 Returns the name (an integer) of the OpenGL Shading Language
    "program" object.
   getRadius()   - 
Method in class org.proteinshader.graphics.displaylists. SphereListInfo 
 Returns the radius used to draw the sphere in the display list.
   getRadius()   - 
Method in class org.proteinshader.structure. Drawable 
 Returns the radius.
   getRadius()   - 
Method in enum org.proteinshader.structure.enums. BondEnum 
 Returns a radius that can be used for representing the Bond as a
    cylinder.
   getRecord(String)   - 
Method in class org.proteinshader.structure. Category 
 Retrieves a Record by its name.
   getRecord(String, String)   - 
Method in class org.proteinshader.structure. Description 
 Retrieves a Record by its Category name and Record name.
   getRed()   - 
Method in class org.proteinshader.structure. Drawable 
 Returns the red component of the RGBA color.
   getRed()   - 
Method in enum org.proteinshader.structure.enums. AAColorEnum 
 Returns the red component of the RGBA color as a float in the
    range from 0.0 to 1.0, inclusive.
   getRed()   - 
Method in enum org.proteinshader.structure.enums. AminoAcidEnum 
 Returns the red component of the RGBA color on a scale from 0.0 to
    1.0, inclusive.
   getRed()   - 
Method in enum org.proteinshader.structure.enums. AtomEnum 
 Returns the red component of the RGBA color on a scale from 0.0 to
    1.0, inclusive.
   getRed()   - 
Method in enum org.proteinshader.structure.enums. BondEnum 
 Returns the red component of the RGBA color on a scale from 0.0
    to 1.0, inclusive.
   getRed()   - 
Method in enum org.proteinshader.structure.enums. CPKColorEnum 
 Returns the red component of the RGBA color as a float in the
    range from 0.0 to 1.0, inclusive.
   getRed()   - 
Method in enum org.proteinshader.structure.enums. RegionColorEnum 
 Returns the red component of the RGBA color as a float in the
    range from 0.0 to 1.0, inclusive.
   getRed()   - 
Method in enum org.proteinshader.structure.enums. RegionEnum 
 Returns the red component of the RGBA color on a scale from 0.0 to
    1.0, inclusive.
   getRegionID()   - 
Method in class org.proteinshader.structure. AminoAcid 
 Returns the regionID if the AminoAcid has been assigned to a Region.
   getRegionType()   - 
Method in class org.proteinshader.structure. AminoAcid 
 Returns the region type (LOOP, HELIX, or BETA_STRAND) if the
    AminoAcid has been assigned to a Region.
   getRegionType()   - 
Method in class org.proteinshader.structure. Segment 
 Returns the type of Region that this Segment belongs to.
   getResidue(String)   - 
Method in class org.proteinshader.structure. Chain 
 Returns the Residue with the residueID given as an argument.
   getResidue(String, String, String)   - 
Method in class org.proteinshader.structure. Structure 
 Returns the Residue with the IDs given as arguments.
   getResidueID()   - 
Method in class org.proteinshader.structure. Atom 
 Returns the ID of the Residue that this Atom belongs to.
   getResidueID()   - 
Method in class org.proteinshader.structure. Residue 
 Returns the ID of this Residue.
   getReverseTangent()   - 
Method in class org.proteinshader.math. LocalFrame 
 Converts the rotation (a Quaternion) into a 3 x 3 rotation matrix
    and the returns the third column vector (the Tangent) after
    reversing its direction.
   getRotation()   - 
Method in class org.proteinshader.structure. AminoAcid 
 Returns the rotation (the equivalent of a Frenet Frame) that will
    be needed for creating a Segment object based on this AminoAcid.
   getSaveTitle()   - 
Method in enum org.proteinshader.gui.enums. ImageFormatEnum 
 Returns the a title suitable for use with a save-type file chooser.
   getScale()   - 
Method in class org.proteinshader.gui.components.controlpanel. AtomScalePanel 
 Returns the scale currently showing in the 'Scale:' spinner.
   getScale()   - 
Method in class org.proteinshader.gui. Renderer 
 Returns the scale factor for the image.
   getScale()   - 
Method in class org.proteinshader.gui.viewing. Rotation 
 Returns the scale factor for the image.
   getSegment()   - 
Method in class org.proteinshader.structure. Atom 
 Returns the Segment the Atom is associated with (or null if
    the Atom is not associated with any Segment).
   getSegment()   - 
Method in class org.proteinshader.structure. Bond 
 Returns the Segment the Bond is associated with (or null if
    the Bond is not associated with any Segment).
   getSegment(String, String, RegionEnum)   - 
Method in class org.proteinshader.structure. Model 
 Returns the requested Segment if it exists.
   getSegment(String)   - 
Method in class org.proteinshader.structure. Region 
 Returns the Segment with the requested segmentID (if it exists).
   getSegmentID()   - 
Method in class org.proteinshader.structure. Segment 
 Returns the Segment ID, which is the same as the Residue ID of the
    AminoAcid this Segment represents.
   getSegmentListInfo(Segment)   - 
Method in class org.proteinshader.graphics.displaylists. SegmentReferences 
 Returns the SegmentListInfo object that stores information on
    the OpenGL display lists that have been cached for the Segment
    given as an argument.
   getSelectedAtom()   - 
Method in class org.proteinshader.gui.components.controlpanel. SelectorPanel 
 Returns the currently selected Atom from the Atom list.
   getSelectedAtoms()   - 
Method in class org.proteinshader.gui.components.controlpanel. SelectorPanel 
 Returns an array of the currently selected Atoms from the Atom
    list.
   getSelectedChain()   - 
Method in class org.proteinshader.gui.components.controlpanel. SelectorPanel 
 Returns the currently selected Chain from the Chain menu.
   getSelectedFileWithFormatExt()   - 
Method in class org.proteinshader.gui.utils. ImageFileChooser 
 After calling on getSelectedFile() of the JFileChooser parent class, this
    method will check that the filename has the correct extension based on the
    image format type (unless the file is null, in which case null will still
    be returned).
   getSelectedModel()   - 
Method in class org.proteinshader.gui.components.controlpanel. SelectorPanel 
 Returns the currently selected Model from the Model menu.
   getSelectedResidue()   - 
Method in class org.proteinshader.gui.components.controlpanel. SelectorPanel 
 Returns the currently selected Residue from the Residue list.
   getSelectedResidues()   - 
Method in class org.proteinshader.gui.components.controlpanel. SelectorPanel 
 Returns an array of the currently selected Residues from the
    Residue list.
   getSelectedResidueType()   - 
Method in class org.proteinshader.gui.components.controlpanel. SelectorPanel 
 Returns the currently selected Residue type from the Residue menu.
   getSense()   - 
Method in class org.proteinshader.structure. BetaStrand 
 Returns the sense (orientation) of the BetaStrand.
   getSense()   - 
Method in class org.proteinshader.structure.io. BetaStrandRecord 
 Returns the sense (orientation) of the BetaStrand.
   getSerialNo()   - 
Method in class org.proteinshader.structure. Atom 
 Returns the Atom serial number.
   getSerialNo()   - 
Method in class org.proteinshader.structure. Helix 
 Returns the serial number of the Helix.
   getSerialNo()   - 
Method in class org.proteinshader.structure.io. HelixRecord 
 Returns the serial number of the Helix.
   getShape()   - 
Method in class org.proteinshader.structure. Helix 
 Returns the shape that should be used to represent the Helix.
   getSheetID()   - 
Method in class org.proteinshader.structure. BetaStrand 
 Returns the ID of the sheet that the BetaStrand belongs to.
   getSheetID()   - 
Method in class org.proteinshader.structure.io. BetaStrandRecord 
 Returns the ID of the sheet the BetaStrand belongs to.
   getShortName()   - 
Method in enum org.proteinshader.gui.enums. ImageFormatEnum 
 Returns the short standard name of the ImageFormatEnum (usually 3 letters).
   getSideChainStyle()   - 
Method in class org.proteinshader.structure. Segment 
 Gets the side chain style (NONE, SPACE_FILLING, STICK_AND_BALLS,
    or STICKS).
   getSixCharName()   - 
Method in enum org.proteinshader.structure.io.enums. RecordEnum 
 Returns the 6 character name for a PDB record type.
   getSlices()   - 
Method in class org.proteinshader.graphics.displaylists. CylinderListInfo 
 Returns the number of slices that were used to draw the cylinder
    stored in the display list.
   getSlices()   - 
Method in class org.proteinshader.graphics.displaylists. SphereListInfo 
 Returns the number of slices that were used to draw the sphere
    stored in the display list.
   getSliderValue()   - 
Method in class org.proteinshader.gui.components.controlpanel. VisibilityPanel 
 Gets the current value for the slider.
   getSpaceFillingRef()   - 
Method in class org.proteinshader.graphics.displaylists. SphereReferences 
 Returns the name (an integer) of an OpenGL display list for a
    sphere intended to be used for Space-Filling style displays.
   getSpaceFillingRef(double)   - 
Method in class org.proteinshader.graphics.displaylists. SphereReferences 
 Returns the name (an integer) of an OpenGL display list for a
    sphere intended to be used for a Space-Filling style display.
   getSpaceFillingSlices()   - 
Method in class org.proteinshader.graphics.displaylists. SphereReferences 
 Returns the number of slices that should be used for a sphere if automatic
    calculation of tiling number is turned off.
   getSpaceFillingSphereInfo()   - 
Method in class org.proteinshader.graphics.displaylists. SphereReferences 
 Returns the SphereListInfo object that holds the information on an
    OpenGL display list for a sphere to be used for SPACE_FILLING style.
   getSpaceFillingStacks()   - 
Method in class org.proteinshader.graphics.displaylists. SphereReferences 
 Returns the number of stacks that should be used for a sphere if automatic
    calculation of tiling number is turned off.
   getSpaceFillingTilingNumber(double)   - 
Method in class org.proteinshader.graphics.displaylists. SphereReferences 
 Returns the recommended level of detail (tiling number = slices = stacks)
    for rendering a sphere at a given camera distance in angstroms.
   getSpecularExp()   - 
Method in class org.proteinshader.structure. Drawable 
 Returns the specular exponent for use in lighting calculations.
   getSphereInfo(StyleEnum)   - 
Method in class org.proteinshader.graphics.adapter. StructureToGraphics 
 Returns the SphereListInfo object that holds the information on
    an OpenGL display list for a sphere that is currently used for
    drawing Atoms.
   getSphereInfo(StyleEnum)   - 
Method in interface org.proteinshader.gui. Mediator 
 Returns the SphereListInfo object that holds the information on an
    OpenGL display list for a sphere that is currently used for
    drawing Atoms.
   getSphereInfo(StyleEnum)   - 
Method in class org.proteinshader.gui. MediatorImpl 
 Returns the SphereListInfo object that holds the information on an
    OpenGL display list for a sphere that is currently used for
    drawing Atoms.
   getSrcAtom()   - 
Method in class org.proteinshader.structure. Bond 
 Returns the source Atom for this Bond.
   getSrcAtomSerialNo()   - 
Method in exception org.proteinshader.structure.exceptions. BondLengthException 
 Returns the source Atom PDB serial number.
   getSrcAtomType()   - 
Method in exception org.proteinshader.structure.exceptions. BondLengthException 
 Returns the source Atom type as an AtomEnum.
   getSrcDirX()   - 
Method in class org.proteinshader.structure. Bond 
 Returns the x-coordinate of the center to source Atom direction
    vector.
   getSrcDirY()   - 
Method in class org.proteinshader.structure. Bond 
 Returns the y-coordinate of the center to source Atom direction
    vector.
   getSrcDirZ()   - 
Method in class org.proteinshader.structure. Bond 
 Returns the z-coordinate of the center to source Atom direction
    vector.
   getStacks()   - 
Method in class org.proteinshader.graphics.displaylists. CylinderListInfo 
 Returns the number of stacks that were used to draw the cylinder
    stored in the display list.
   getStacks()   - 
Method in class org.proteinshader.graphics.displaylists. SphereListInfo 
 Returns the number of stacks that were used to draw the sphere
    stored in the display list.
   getStartCapName()   - 
Method in class org.proteinshader.graphics.displaylists. SegmentListInfo 
 Returns a reference to the OpenGL display list for the start cap.
   getStartChar()   - 
Method in class org.proteinshader.graphics.typography. GLF2Header 
 Returns the start char (the first character in the glyph set).
   getStartResidueID()   - 
Method in class org.proteinshader.structure.io. BetaStrandRecord 
 Returns the start Residue ID for the BetaStrand.
   getStartResidueID()   - 
Method in class org.proteinshader.structure.io. HelixRecord 
 Returns the start Residue ID for the Helix.
   getStartResidueID()   - 
Method in class org.proteinshader.structure. Region 
 Returns the ID of the first Residue in the sequence.
   getStartRotation()   - 
Method in class org.proteinshader.structure. Segment 
 Returns a clone of the Quaternion for the start rotation of this
    Segment.
   getSticksCylinderInfo()   - 
Method in class org.proteinshader.graphics.displaylists. CylinderReferences 
 Returns the CylinderListInfo object that holds the information on
    an OpenGL display list for a cylinder to be used for a STICKS
    style display.
   getSticksRef()   - 
Method in class org.proteinshader.graphics.displaylists. CylinderReferences 
 Returns the name (an integer) of an OpenGL display list for a
    cylinder intended to be used for Sticks style displays.
   getSticksRef(double)   - 
Method in class org.proteinshader.graphics.displaylists. CylinderReferences 
 Returns the name (an integer) of an OpenGL display list for a
    cylinder intended to be used for Sticks style displays.
   getStrandsInSheet()   - 
Method in class org.proteinshader.structure. BetaStrand 
 Returns the total number of BetaStrands in the same sheet as this
    BetaStrand.
   getStrandsInSheet()   - 
Method in class org.proteinshader.structure.io. BetaStrandRecord 
 Returns the total number of strands in the same sheet.
   getStructure()   - 
Method in interface org.proteinshader.gui. Mediator 
 Returns the current Structure to display.
   getStructure()   - 
Method in class org.proteinshader.gui. MediatorImpl 
 Returns the current Structure to display.
   getStructureID()   - 
Method in class org.proteinshader.structure. Atom 
 Returns the ID of the Structure that this Atom belongs to.
   getStructureID()   - 
Method in class org.proteinshader.structure. Chain 
 Returns the structureID of the Structure this Chain belongs to.
   getStructureID()   - 
Method in class org.proteinshader.structure. Description 
 Returns the ID of the Structure that owns this Description.
   getStructureID()   - 
Method in class org.proteinshader.structure. Model 
 Returns the structureID of the Structure this Model belongs to.
   getStructureID()   - 
Method in class org.proteinshader.structure. Region 
 Returns the ID of the Structure that this Region belongs to.
   getStructureID()   - 
Method in class org.proteinshader.structure. Residue 
 Returns the ID of the Structure that this Residue belongs to.
   getStructureID()   - 
Method in class org.proteinshader.structure. Structure 
 Returns the structureID, which is the sames as the PDB unique ID
    code.
   getStyle()   - 
Method in class org.proteinshader.graphics.adapter. StructureToGraphics 
 Returns the current style (TUBES, RIBBONS, RIBBONS_AND_TUBES, FRENET_FRAMES,
    SPACE_FILLING, BALLS_AND_STICKS, or STICKS).
   getStyle()   - 
Method in class org.proteinshader.graphics.displaylists. GeometricListInfo 
 Returns the style that the geometric object is intended for: SPACE_FILLING,
    BALLS_AND_STICKS, or STICKS.
   getStyle()   - 
Method in interface org.proteinshader.gui. Mediator 
 Returns the current style (TUBES, RIBBONS, RIBBONS_AND_TUBES, 
    FRENET_FRAMES, SPACE_FILLING, BALLS_AND_STICKS, or STICKS).
   getStyle()   - 
Method in class org.proteinshader.gui. MediatorImpl 
 Returns the current style (TUBES, RIBBONS, RIBBONS_AND_TUBES, FRENET_FRAMES,
    SPACE_FILLING, BALLS_AND_STICKS, or STICKS).
   getStyleMenu()   - 
Method in class org.proteinshader.gui.components.menubar. MainMenuBar 
 Returns the StyleMenu, which has a method for setting the style back to 
    its default.
   getTangent()   - 
Method in class org.proteinshader.math. LocalFrame 
 Converts the rotation (a Quaternion) into a 3 x 3 rotation matrix
    and the returns the third column vector (the Tangent).
   getTangent()   - 
Method in class org.proteinshader.math. Quaternion 
 Converts the Quaternion into a 3 x 3 rotation matrix and the
    returns the third column vector (the Tangent).
   getTemperature()   - 
Method in class org.proteinshader.structure. Atom 
 Returns the temperature factor from x-ray crystallography.
   getThinSidesName()   - 
Method in class org.proteinshader.graphics.displaylists. SegmentListInfo 
 Returns the reference to the OpenGL display list for the thin
    sides of a Ribbon.
   getThreeLetterCode()   - 
Method in enum org.proteinshader.structure.enums. AminoAcidEnum 
 Returns the three-letter code for the amino acid.
   getTolerance()   - 
Method in class org.proteinshader.structure.visitor. BondPredictor 
 Returns the Bond length tolerance that is currently in use.
   getToleranceFactor()   - 
Method in exception org.proteinshader.structure.exceptions. BondLengthException 
 Returns the tolerance factor used to determine that the calulated
    length was too far from the expected length.
   getToolsMenu()   - 
Method in class org.proteinshader.gui.components.menubar. MainMenuBar 
 Returns the ToolsMenu, which has methods for detecting and
    changing the currently selected Tools menu item ("Selector"
    or "Controls").
   getTopRadius()   - 
Method in class org.proteinshader.graphics.displaylists. CylinderListInfo 
 Returns the top radius of the cylinder in the display list.
   getTranslation()   - 
Method in class org.proteinshader.math. LocalFrame 
 Returns a copy of the translation vector held by this LocalFrame.
   getTranslation()   - 
Method in class org.proteinshader.structure. AminoAcid 
 Returns a new Ved3d object based on the xyz-coordinates of the
    alpha-carbon.
   getTranslation()   - 
Method in class org.proteinshader.structure. Drawable 
 Returns the xyz-center of the Drawable as a vector.
   getTranslucentDrawables()   - 
Method in class org.proteinshader.structure. Model 
 Copies the list of translucent Drawables into an array and returns
    the array.
   getType()   - 
Method in class org.proteinshader.structure. AminoAcid 
 Returns the type of amino aicd: ALA, ARG, ASN,  etc. 

    The type cannot be null because it is checked by the constructor.
   getType()   - 
Method in class org.proteinshader.structure. Atom 
 Returns the Atom's type as an enum based on the periodic table of
    the elements.
   getType()   - 
Method in class org.proteinshader.structure. Bond 
 Returns the Bond's type as an enum (SINGLE, DOUBLE, PEPTIDE,
     etc. ).
   getType()   - 
Method in class org.proteinshader.structure. Helix 
 Returns the type of Helix.
   getType()   - 
Method in class org.proteinshader.structure.io. HelixRecord 
 Returns the type of Helix.
   getTypeface()   - 
Method in class org.proteinshader.graphics.typography. GlyphSet 
 Returns the typeface for the glyphs (bold, italic, regular,
     etc .).
   getUnknownChars()   - 
Method in class org.proteinshader.graphics.typography. GlyphSet 
 Returns a String composed of any characters that were missing (no
    corresponding Glyph) when the last call to getGlyphs(text) was
    made.
   getVanDerWaalsRadius()   - 
Method in enum org.proteinshader.structure.enums. AtomEnum 
 Returns the van der Waals radius for the Atom, which should be
    useful for representing the Atom as a sphere.
   getVertFile()   - 
Method in enum org.proteinshader.graphics.enums. ShaderEnum 
 Returns the name of the file the vertex shader is stored in.
   getVertFile()   - 
Method in class org.proteinshader.graphics.shader. ShaderProgram 
 Returns the File the vertex shader source code was obtained from.
   getVertShaderName()   - 
Method in class org.proteinshader.graphics.shader. ShaderProgram 
 Returns the name (an integer) of the vertex shader object.
   getVisibility()   - 
Method in class org.proteinshader.structure. Drawable 
 Returns the visibility status of the Drawable object.
   getVisibilityMenu()   - 
Method in class org.proteinshader.gui.components.menubar. MainMenuBar 
 Returns the VisibilityMenu, which has methods for enabling/disabling
    or selecting/deselecting a menu item ('Amino Acids Allowed', 
    'Heterogens Allowed', or 'Water Allowed').
   getWater(String)   - 
Method in class org.proteinshader.structure. Chain 
 Returns the Water with the residueID given as an argument.
   getWidth()   - 
Method in class org.proteinshader.graphics.typography. Glyph 
 Returns the width of the glyph in pixels.
   getWidth()   - 
Method in class org.proteinshader.graphics.typography. TextLabel 
 Returns the width of the glyph in pixels.
   getWidth()   - 
Method in class org.proteinshader.structure. Model 
 Returns the width of the Model (maxX - minX).
   getX()   - 
Method in class org.proteinshader.structure. Drawable 
 Returns the x-coordinate of the Drawable's center.
   getX()   - 
Method in class org.proteinshader.structure. Model 
 Returns the x-coordinate for the center of gravity of the Model.
   getX1()   - 
Method in exception org.proteinshader.graphics.exceptions. GlyphImageException 
 Returns the x-coordinate of the top left corner of the glyph.
   getX1()   - 
Method in class org.proteinshader.graphics.typography. Glyph 
 Returns the x-coordinate of the top left corner of the glyph
    (this coordinate is used for extracting the glyph from the
    larger image file with several glyphs).
   getX2()   - 
Method in exception org.proteinshader.graphics.exceptions. GlyphImageException 
 Returns the x-coordinate of the bottom right corner of the glyph.
   getX2()   - 
Method in class org.proteinshader.graphics.typography. Glyph 
 Returns the x-coordinate of the bottom right corner of the glyph
    (this coordinate is used for extracting the glyph from the
    larger image file with several glyphs).
   getY()   - 
Method in class org.proteinshader.structure. Drawable 
 Returns the y-coordinate of the Drawable's center.
   getY()   - 
Method in class org.proteinshader.structure. Model 
 Returns the y-coordinate for the center of gravity of the Model.
   getY1()   - 
Method in exception org.proteinshader.graphics.exceptions. GlyphImageException 
 Returns the y-coordinate of the top left corner of the glyph.
   getY1()   - 
Method in class org.proteinshader.graphics.typography. Glyph 
 Returns the y-coordinate of the top left corner of the glyph
    (this coordinate is used for extracting the glyph from the
    larger image file with several glyphs).
   getY2()   - 
Method in exception org.proteinshader.graphics.exceptions. GlyphImageException 
 Returns the y-coordinate of the bottom right corner of the glyph.
   getY2()   - 
Method in class org.proteinshader.graphics.typography. Glyph 
 Returns the y-coordinate of the bottom right corner of the glyph
    (this coordinate is used for extracting the glyph from the
    larger image file with several glyphs).
   getZ()   - 
Method in class org.proteinshader.structure. Drawable 
 Returns the z-coordinate of the Drawable's center.
   getZ()   - 
Method in class org.proteinshader.structure. Model 
 Returns the z-coordinate for the center of gravity of the Model.
   GLF2GlyphSetFactory   - Class in  org.proteinshader.graphics.typography  Reads a set of glyphs from a ".glf" file that was created with the
glFont program (Version 2.0) written by Brad Fish
(brad.fish@gmail.com).   GLF2GlyphSetFactory()   - 
Constructor for class org.proteinshader.graphics.typography. GLF2GlyphSetFactory 
 Constructs a GLF2GlyphSetFactory.
   GLF2Header   - Class in  org.proteinshader.graphics.typography  Stores information from the header of a '.glf' file.   GLF2Header(String, int, int, int, int)   - 
Constructor for class org.proteinshader.graphics.typography. GLF2Header 
 Constructs a GLF2Header.
   Glyph   - Class in  org.proteinshader.graphics.typography  Stores a 2D array of bytes that form a graphical representation of a
character.   Glyph(String, int, int, int, int, int)   - 
Constructor for class org.proteinshader.graphics.typography. Glyph 
 Constructs a Glyph.
   GlyphConfigException   - Exception in  org.proteinshader.graphics.exceptions  To report errors that occur while creating a Glyph.   GlyphConfigException(String, String)   - 
Constructor for exception org.proteinshader.graphics.exceptions. GlyphConfigException 
 The message given this constructor can be retrieved using
    getMessage().
   GlyphDemo   - Class in  org.proteinshader.graphics.typography  Provides a driver to perform some basic tests on classes in the
typography package.   GlyphDemo()   - 
Constructor for class org.proteinshader.graphics.typography. GlyphDemo 
  
   GlyphException   - Exception in  org.proteinshader.graphics.exceptions  To report errors that occur while creating a Glyph.   GlyphException()   - 
Constructor for exception org.proteinshader.graphics.exceptions. GlyphException 
 Sets the default message to 'An error occurred while creating a
    Glyph'.
   GlyphException(String)   - 
Constructor for exception org.proteinshader.graphics.exceptions. GlyphException 
 The message given this constructor can be retrieved using
    getMessage().
   GlyphImageException   - Exception in  org.proteinshader.graphics.exceptions  To report errors that occur while creating a Glyph.   GlyphImageException(String, int, int, int, int, int, int, int, int, int)   - 
Constructor for exception org.proteinshader.graphics.exceptions. GlyphImageException 
 The message given this constructor can be retrieved using
    getMessage().
   GlyphSet   - Class in  org.proteinshader.graphics.typography  Stores a set of Glyph objects.   GlyphSet(String, String, String, int, int, int, int)   - 
Constructor for class org.proteinshader.graphics.typography. GlyphSet 
 Constructs a GlyphSet.
   GlyphSetFactory   - Interface in  org.proteinshader.graphics.typography  Defines the interface for a factory that can read glyphs from a source
and produce a GlyphSet object.   GlyphSetFactoryFactory   - Class in  org.proteinshader.graphics.typography  Use Java reflection to load a concrete GlyphSetFactory based on
information held in a Properties object.   GlyphSetFactoryFactory()   - 
Constructor for class org.proteinshader.graphics.typography. GlyphSetFactoryFactory 
 Constructs a TextLabelFactory.
 
 
    
 H  
 
   hadUnknownChars()   - 
Method in class org.proteinshader.graphics.typography. GlyphSet 
 Returns true if the last call to getGlyphs(text) had any unknown
    characters in the String (unknown in the sense that the GlyphSet
    did not have a Glyph for a character).
   HALF_FOVY   - 
Static variable in class org.proteinshader.gui.viewing. Camera 
 Half the fovy is useful for some calculations.
   HALF_FOVY_RADIANS   - 
Static variable in class org.proteinshader.gui.viewing. Camera 
 Half the fovy needs to be in radians for Java trig functions.
   HALF_PI   - 
Static variable in class org.proteinshader.graphics. Shape 
 One-half Pi is defined as 1.57079632679489.
   HALF_PI   - 
Static variable in class org.proteinshader.gui.viewing. Rotation 
 One-half Pi is defined as 1.57079632679489, 
        and is equal to 90 degrees.
   HALF_PI   - 
Static variable in class org.proteinshader.structure.factory. BetaStrandSegmentFactory 
 90 degrees is half the value of PI radians.
   HALF_PI   - 
Static variable in class org.proteinshader.structure.io. TubeVertices 
 One-half Pi is defined as 1.57079632679489.
   HALFTONING_CONFIG   - 
Static variable in class org.proteinshader.graphics.textures. TextureFactory 
 Stores the name of the halftoning configuration file,
        which is currently set to 'halftoning_textures.conf'.
   HALFTONING_DIRECTORY   - 
Static variable in class org.proteinshader.graphics.textures. TextureFactory 
 Stores the relative pathname of the halftoning textures
        directory.
   hasAminoAcids()   - 
Method in class org.proteinshader.structure. Model 
 Returns true if the model holds any AminoAcids.
   hasAtomModifier()   - 
Method in class org.proteinshader.structure.visitor. ModifierVisitor 
 Returns true if this ModifierVisitor holds an AtomModifier object.
   hasBondModifier()   - 
Method in class org.proteinshader.structure.visitor. ModifierVisitor 
 Returns true if this ModifierVisitor holds a BondModifier object.
   hasHeterogens()   - 
Method in class org.proteinshader.structure. Model 
 Returns true if the model holds any Heterogens.
   hasSegmentModifier()   - 
Method in class org.proteinshader.structure.visitor. ModifierVisitor 
 Returns true if this ModifierVisitor holds a SegmentModifier
    object.
   hasWaters()   - 
Method in class org.proteinshader.structure. Model 
 Returns true if the model holds any Waters.
   HEADER_BYTES   - 
Static variable in class org.proteinshader.graphics.typography. GLF2Header 
 The header in a '.glf' file has 24 bytes.
   HEIGHT   - 
Static variable in class org.proteinshader.graphics.typography. TextLabel 
 The height is 32 pixels.
   HEIGHT_INDEX   - 
Static variable in class org.proteinshader.graphics.typography. GLF2Header 
 The bitmap height integer starts at index 8 of the header.
   Helix   - Class in  org.proteinshader.structure  This concrete subclass of Region holds information specific to a
protein Helix.   Helix(String, String, String, String, Chain, HelixEnum, HelixShapeEnum)   - 
Constructor for class org.proteinshader.structure. Helix 
 Constructs a Helix.
   HelixEnum   - Enum in  org.proteinshader.structure.enums  Provides an enumeration of the helix types specified in the
  Protein Data Bank  .   HelixRecord   - Class in  org.proteinshader.structure.io  This class is used by the PDBLineParser to temporarily store HELIX
records.   HelixRecord(String, String, String, String, String, HelixEnum)   - 
Constructor for class org.proteinshader.structure.io. HelixRecord 
 Constructs a HelixRecord.
   HelixShapeEnum   - Enum in  org.proteinshader.structure.enums  Lists the possible shapes that can be used to represent a Helix.   HELP_FILE   - 
Static variable in class org.proteinshader.gui.components.menubar. HelpMenu 
 Holds the path to the index.html file in the help directory.
   HelpMenu   - Class in  org.proteinshader.gui.components.menubar  Provides an "About" box and help for using the ProteinShader program.   HelpMenu(Mediator, Frame)   - 
Constructor for class org.proteinshader.gui.components.menubar. HelpMenu 
 Constructs a HelpMenu.
   HelpMenuListenerFactory   - Class in  org.proteinshader.gui.listeners.menubar  Creates all event listeners needed by the HelpMenu of the MainMenuBar.   HelpMenuListenerFactory(Mediator, HelpMenu)   - 
Constructor for class org.proteinshader.gui.listeners.menubar. HelpMenuListenerFactory 
 Constructs a HelpMenuListenerFactory.
   Hermite   - Class in  org.proteinshader.math  Calculates a cubic equation between two control points so that points
on the curve in between can be interpolated.   Hermite(Point3d, Point3d, Vec3d, Vec3d)   - 
Constructor for class org.proteinshader.math. Hermite 
 Constructs a Hermite object by using the input points and vectors
    to calculate and store the coefficients needed for the cubic
    equations for x(t), y(t), and z(t).
   Hermite(Vec3d, Vec3d, Vec3d, Vec3d)   - 
Constructor for class org.proteinshader.math. Hermite 
 Constructs a Hermite object by using the input vectors to
    calculate and store the coefficients needed for the cubic
    equations for x(t), y(t), and z(t).
   HermiteDemo   - Class in  org.proteinshader.math  Performs some simple tests on the Hermite class.   HermiteDemo()   - 
Constructor for class org.proteinshader.math. HermiteDemo 
  
   Heterogen   - Class in  org.proteinshader.structure  This concrete subclass of the abstract class Residue stores
information on a heterogen in a PDB structure entry.   Heterogen(String, String, String, String, String)   - 
Constructor for class org.proteinshader.structure. Heterogen 
 Creates a Heterogen with the name and IDs given as arguments
    (after adding the HETEROGEN_PREFIX ("HET_") to the beginning of
    residueID).
   HETEROGEN_PREFIX   - 
Static variable in class org.proteinshader.structure. Heterogen 
 'HET_' will be added to the beginning the residueID
        for a Heterogen (if it is not already present).
 
 
    
 I  
 
   IDTest   - Interface in  org.proteinshader.structure  This interface declares a processID() method that is intended to be
used as a helper method for constructors that need to perform tests
and/or any modifications on an ID.   IMAGE_FILE   - 
Static variable in class org.proteinshader.gui.components.menubar. HelpMenu 
 Holds path to the image file for the "About ProteinShader" box.
   IMAGE_FILE   - 
Static variable in class org.proteinshader.gui. ProteinShaderSplasher 
  
   ImageFileChooser   - Class in  org.proteinshader.gui.utils  Extends JFileChooser by requiring that an ImageFormatEnum be given to the 
constructor.   ImageFileChooser(ImageFormatEnum, String)   - 
Constructor for class org.proteinshader.gui.utils. ImageFileChooser 
 Constructs a ImageFileChooser.
   ImageFileChooser(ImageFormatEnum, File)   - 
Constructor for class org.proteinshader.gui.utils. ImageFileChooser 
 Constructs a ImageFileChooser.
   ImageFormatEnum   - Enum in  org.proteinshader.gui.enums  Provides an enumeration of the image file formats.   IMAGES_DIRECTORY   - 
Static variable in class org.proteinshader.gui.components.menubar. FileMenu 
 Specifies the directory for saving JPEG, PNG, and GIF images.
   includeAAHetAndWater(boolean, boolean, boolean)   - 
Method in class org.proteinshader.structure.visitor. Visitor 
 Sets the mode to include or exclude AminoAcids, Heterogens,
    and Waters.
   includeAtoms(boolean)   - 
Method in class org.proteinshader.structure.visitor. VisibilityVisitor 
 Determines whether Atoms should be included when the lists of
    opaque and translucent Drawables are filled.
   includeBonds(boolean)   - 
Method in class org.proteinshader.structure.visitor. VisibilityVisitor 
 Determines whether Bonds should be included when the lists of
    opaque and translucent Drawables are filled.
   includeSegments(boolean)   - 
Method in class org.proteinshader.structure.visitor. VisibilityVisitor 
 Determines whether Segments should be included when the lists of
    opaque and translucent Drawables are filled.
   INCREMENT   - 
Static variable in class org.proteinshader.gui.components. SpringLoadedJFrame 
 Sets the increment for the timer circuit
        used to open the spring-loaded frame.
   INDENTATION_FACTOR   - 
Static variable in class org.proteinshader.structure.visitor. SFWriterVisitor 
  
   init(GLAutoDrawable)   - 
Method in class org.proteinshader.gui. Renderer 
 This method is called by the canvas immediately after the OpenGL
    state is initialized.
   INITIAL_STYLE   - 
Static variable in class org.proteinshader.gui.components.menubar. StyleMenu 
 The style to set as selected in the 'Style' menu.
   initializeViewport(GLAutoDrawable, GL)   - 
Method in class org.proteinshader.gui.viewing. Camera 
 Sets the viewport so that the origin (0, 0, 0) is in its center.
   interpolateAndPrint(Quaternion, Quaternion, double)   - 
Method in class org.proteinshader.math. SlerpDemo 
 Interpolates between the two quaternions given as arguments and
    then prints the result quaterion along with the [N B T] equivalent
    to the quaternion.
   interpolateQ1AndQ1()   - 
Method in class org.proteinshader.math. SlerpDemo 
 Interpolates between Q1 (for CYS 273) and itself and prints the
    results.
   interpolateQ1AndQ2()   - 
Method in class org.proteinshader.math. SlerpDemo 
 Interpolates between Q1 (for CYS 273) and Q2 (for GLY 274) and
    prints the results.
   interpolateQ2AndQ3()   - 
Method in class org.proteinshader.math. SlerpDemo 
 Interpolates between Q2 (for GLY 274) and Q3 (for GLY 275) and
    prints the results.
   InvalidIDException   - Exception in  org.proteinshader.structure.exceptions  Used to report that an ID is invalid or missing.   InvalidIDException()   - 
Constructor for exception org.proteinshader.structure.exceptions. InvalidIDException 
 Sets the default message to "ERROR: A missing or invalid ID was
    detected".
   InvalidIDException(String)   - 
Constructor for exception org.proteinshader.structure.exceptions. InvalidIDException 
 The message given this constructor can be retrieved using
    getMessage().
   InvalidRegionException   - Exception in  org.proteinshader.structure.exceptions  Used to report that a Region (Helix or BetaStrand) is invalid because
its start and end Residue IDs could not be matched to an existing
sequence of Residues in a Chain.   InvalidRegionException()   - 
Constructor for exception org.proteinshader.structure.exceptions. InvalidRegionException 
 Sets the default message to

    "ERROR: The start and end residues of a region are invalid".
   InvalidRegionException(String)   - 
Constructor for exception org.proteinshader.structure.exceptions. InvalidRegionException 
 The message given this constructor can be retrieved using
    getMessage().
   invert()   - 
Method in class org.proteinshader.math. Quaternion 
 Creates a clone of the calling Quaternion and inverts it.
   invertMe()   - 
Method in class org.proteinshader.math. Quaternion 
 Inverts the calling Quaternion.
   isAnimating()   - 
Method in class org.proteinshader.gui. Renderer 
 Returns true if an animation (model rotation) is in progress.
   isBaseCapped()   - 
Method in class org.proteinshader.graphics.displaylists. CylinderListInfo 
 Returns true if a capping sphere was used at the base of the
    cylinder.
   isDistanceAcceptable(Atom, Atom)   - 
Method in class org.proteinshader.structure.visitor. BondPredictor 
 Determines if two Atoms are within an acceptable distance for a
    covalent Bond.
   isDoubleBond(Atom, Atom, AminoAcidEnum)   - 
Method in class org.proteinshader.structure.visitor. BondPredictor 
 Uses the Atom IDs (from Atom name field in a PDB record) and the
    AminoAcid type to determine if the Atoms should have a double Bond
    (assuming that they both belong to the same AminoAcid).
   isEndCapped()   - 
Method in class org.proteinshader.structure. Segment 
 Returns a boolean value to indicate if the end of the Segment should be 
    capped when it is drawn.
   isStartCapped()   - 
Method in class org.proteinshader.structure. Segment 
 Returns a boolean value to indicate if the start of the Segment should be 
    capped when it is drawn.
   isTopCapped()   - 
Method in class org.proteinshader.graphics.displaylists. CylinderListInfo 
 Returns true if a capping sphere was used at the top of the
    cylinder.
   iteratorAminoAcids()   - 
Method in class org.proteinshader.structure. Chain 
 Returns an Iterator for the AminoAcids held by this Chain.
   iteratorAminoAcids()   - 
Method in class org.proteinshader.structure. Region 
 Returns an Iterator for the AminoAcids referred to by this Region.
   iteratorAtoms()   - 
Method in class org.proteinshader.structure. Residue 
 Returns an Iterator for the Atoms held by this Residue.
   iteratorBackboneAtoms()   - 
Method in class org.proteinshader.structure. AminoAcid 
 Returns an Iterator for the backbone Atoms held by this Residue.
   iteratorBetaStrands()   - 
Method in class org.proteinshader.structure. Chain 
 Returns an Iterator for the BetaStrands held by this Chain.
   iteratorBetaStrands()   - 
Method in class org.proteinshader.structure. Model 
 Returns an Iterator for the BetaStrands held by this Model.
   iteratorBonds()   - 
Method in class org.proteinshader.structure. Atom 
 Returns an Iterator for the Bonds held by this Atom.
   iteratorCategories()   - 
Method in class org.proteinshader.structure. Description 
 Returns an Iterator for the Category objects held by this
    Description.
   iteratorChains()   - 
Method in class org.proteinshader.structure. Model 
 Returns an Iterator for the Chains held by this Model.
   iteratorGlyphs()   - 
Method in class org.proteinshader.graphics.typography. GlyphSet 
 Returns an Iterator for the Glyphs.
   iteratorHelices()   - 
Method in class org.proteinshader.structure. Chain 
 Returns an Iterator for the Helices held by this Chain.
   iteratorHelices()   - 
Method in class org.proteinshader.structure. Model 
 Returns an Iterator for the Helices held by this Model.
   iteratorHeterogens()   - 
Method in class org.proteinshader.structure. Chain 
 Returns an Iterator for the Heterogens held by this Chain.
   iteratorLines()   - 
Method in class org.proteinshader.structure. Record 
 Returns a Iterator for the lines of the Record.
   iteratorLoops()   - 
Method in class org.proteinshader.structure. Chain 
 Returns an Iterator for the Loops held by this Chain.
   iteratorLoops()   - 
Method in class org.proteinshader.structure. Model 
 Returns an Iterator for the Loops held by this Model.
   iteratorModels()   - 
Method in class org.proteinshader.structure. Structure 
 Returns an Iterator for the Models held by this Structure.
   iteratorRecords()   - 
Method in class org.proteinshader.structure. Category 
 Returns an Iterator for the Records held by this Category object.
   iteratorSegments()   - 
Method in class org.proteinshader.structure. Region 
 Returns an Iterator for the Segments owned by this Region.
   iteratorSideChainAtoms()   - 
Method in class org.proteinshader.structure. AminoAcid 
 Returns an Iterator for the side chain Atoms held by this Residue.
   iteratorWaters()   - 
Method in class org.proteinshader.structure. Chain 
 Returns an Iterator for the Water molecules held by this Chain.
 
 
    
 J  
 
   JITTER_4   - 
Static variable in class org.proteinshader.graphics. Silhouette 
 Holds (xPixels, yPixels) values for translating 4 times with jitter.
   JitterEnum   - Enum in  org.proteinshader.gui.enums  Holds the xy-coordinates needed for jittering a scene to accomplish 
antialiasing (the smoothing of jagged edge lines).   jitterScene(JitterEnum)   - 
Method in interface org.proteinshader.gui. Mediator 
 Draws the scene multiple times to an accumulation buffer while jittering
    the xy-coordinates a fraction of a pixel.
   jitterScene(JitterEnum)   - 
Method in class org.proteinshader.gui. MediatorImpl 
 Draws the scene multiple times to an accumulation buffer while jittering
    the xy-coordinates a fraction of a pixel.
   jitterScene(JitterEnum)   - 
Method in class org.proteinshader.gui. Renderer 
 Draws the scene multiple times to an accumulation buffer while jittering
    the xy-coordinates a fraction of a pixel.
 
 
    
 K  
 
   keyPressed(KeyEvent)   - 
Method in class org.proteinshader.gui.listeners. CanvasKeyListener 
 This KeyListener interface method is not needed.
   keyReleased(KeyEvent)   - 
Method in class org.proteinshader.gui.listeners. CanvasKeyListener 
 This KeyListener interface method is not needed.
   keyTyped(KeyEvent)   - 
Method in class org.proteinshader.gui.listeners. CanvasKeyListener 
 Moves the z coordinate for the camera and then redraws the canvas.
 
 
    
 L  
 
   LIGHT0_POSITION   - 
Static variable in class org.proteinshader.gui.viewing. Lighting 
 The position of light zero is {200.0f, 200.0f, 200.0f, 1.0f}.
   Lighting   - Class in  org.proteinshader.gui.viewing  Sets up OpenGL lighting for the Renderer.   Lighting()   - 
Constructor for class org.proteinshader.gui.viewing. Lighting 
 Constructs a Lighting object.
   LIST_FONT   - 
Static variable in class org.proteinshader.gui.components.controlpanel. SelectorPanel 
 The font for lists is 14 point plain Courier.
   loadBendMenu()   - 
Method in class org.proteinshader.gui.components.controlpanel. DecorationsPanel 
 Loads the bend menu with the current set of textures from the Mediator.
   loadFpsFontFile(GL)   - 
Method in class org.proteinshader.gui.viewing. Rotation 
 Tells the FpsLabel object to read in the font file that it needs for 
    displaying a frames per second label on the canvas.
   loadHalftoningMenu()   - 
Method in class org.proteinshader.gui.components.controlpanel. DecorationsPanel 
 Loads the halftoning menu with the current set of textures from the
    Mediator.
   loadPatternsMenu()   - 
Method in class org.proteinshader.gui.components.controlpanel. DecorationsPanel 
 Loads the Patterns menu with the current set of textures from the Mediator,
    and sets the selected item to the second item in the menu (the first
    item is always 'None').
   loadTablaRasa(GL)   - 
Method in class org.proteinshader.graphics.typography. TextLabelManager 
 Loads a blank texture map, the reusable tabla rasa.
   loadTextures(GL)   - 
Method in class org.proteinshader.graphics.adapter. StructureToGraphics 
 Loads the default texture objects needed for text labels and
    real-time halftoning.
   LocalFrame   - Class in  org.proteinshader.math  Stores a local coordinate frame as a rotation (a Quaternion) and a
translation (a Point3d).   LocalFrame()   - 
Constructor for class org.proteinshader.math. LocalFrame 
 Constructs a LocalFrame with the identity quaternion, (0, 0, 0, 1)
    and a translation of (0, 0, 0).
   LocalFrame(Vec3d, Vec3d, Vec3d, double, double, double)   - 
Constructor for class org.proteinshader.math. LocalFrame 
 Constructs a LocalFrame equivalent to the rotation matrix [N B T]
    and the translation (x, y, z).
   LocalFrame(Quaternion, Vec3d)   - 
Constructor for class org.proteinshader.math. LocalFrame 
 Constructs a LocalFrame that holds on to the Quaternion and Vec3d
    given as arguments.
   LocalFrame(double, double, double, double, double, double, double)   - 
Constructor for class org.proteinshader.math. LocalFrame 
 Constructs a LocalFrame using the rotation and translation given
    as arguments.
   LocalFrameDemo   - Class in  org.proteinshader.math  Performs some simple tests on the LocalFrame class.   LocalFrameDemo()   - 
Constructor for class org.proteinshader.math. LocalFrameDemo 
 Creates a LocalFrameDemo object with a few Quaternion objects that
    can be used for testing purposes.
   Loop   - Class in  org.proteinshader.structure  This concrete subclass of Region holds information on
the general "loop" regions found between well-defined
regions of secondary structure (helices and beta-strands).   Loop(String, String, String, Chain)   - 
Constructor for class org.proteinshader.structure. Loop 
 Constructs a Loop.
   LOOP_HEIGHT   - 
Static variable in class org.proteinshader.graphics. Ribbon 
 The default ribbon height (y-axis dimension) for a Loop region
        is 1.0 angstroms.
   LOOP_RADIUS   - 
Static variable in class org.proteinshader.graphics. Tube 
 The default radius for a Loop region waist polygon
        is 0.75 angstroms.
   LOOP_RADIUS   - 
Static variable in class org.proteinshader.structure.io. TubeVertices 
 The default radius for a Loop region waist polygon
        is 0.75 angstroms.
   LOOP_S_COORD_END   - 
Static variable in class org.proteinshader.graphics. Ribbon 
 The texture s-coordinate for loops ends at 0.75 rather than
        at 1.0.
   LOOP_S_COORD_START   - 
Static variable in class org.proteinshader.graphics. Ribbon 
 The texture s-coordinate for loops starts at 0.25 rather than
        at 0.0.
   LOOP_SLICES   - 
Static variable in class org.proteinshader.graphics. Tube 
 The default number of slices for a Loop region waist polygon
        is 10.
   LOOP_SLICES   - 
Static variable in class org.proteinshader.structure.io. TubeVertices 
 The default number of slices for a Loop region waist polygon
        is 8.
   LOOP_STACKS   - 
Static variable in class org.proteinshader.graphics. Ribbon 
 The default number of stacks for a loop segment is 10.
   LOOP_STACKS   - 
Static variable in class org.proteinshader.graphics. Tube 
 The default number of stacks for a loop segment is 10.
   LOOP_STACKS   - 
Static variable in class org.proteinshader.structure.io. TubeVertices 
 The default number of stacks for a loop segment is 10.
   LOOP_THIN_SIDE_S_MAX   - 
Static variable in class org.proteinshader.graphics. Ribbon 
 The texture s-coordinate for the thin side of loops ends at
        0.125.
   LOOP_THIN_SIDE_S_MIN   - 
Static variable in class org.proteinshader.graphics. Ribbon 
 The texture s-coordinate for the thin side of loops starts at
        0.0.
   LOOP_WIDTH   - 
Static variable in class org.proteinshader.graphics. Ribbon 
 The default ribbon width (x-axis dimension) for a Loop region
        is 0.25 angstroms.
   LoopGeneratorVisitor   - Class in  org.proteinshader.structure.visitor  Generates Loop objects from any AminoAcids of a Chain
that do not already belong to a Helix or BetaStrand.   LoopGeneratorVisitor()   - 
Constructor for class org.proteinshader.structure.visitor. LoopGeneratorVisitor 
 Constructs a LoopGeneratorVisitor.
 
 
    
 M  
 
   magnitude()   - 
Method in class org.proteinshader.math. Quaternion 
 Returns the magnitude (length) of this quaternion.
   magnitude()   - 
Method in class org.proteinshader.math. Vec3d 
 Calculates the magnitude of the calling vector.
   main(String[])   - 
Static method in class org.proteinshader.graphics.typography. GlyphDemo 
 Creates an instance of TextLabelFactory and has it read in the
    default set of glyphs.
   main(String[])   - 
Static method in class org.proteinshader.gui. ProteinShader 
 Creates an instance of the ProteinShaderGUI and sets it visible.
   main(String[])   - 
Static method in class org.proteinshader.gui. ProteinShaderSplasher 
 Opens the splash screen before calling main() of ProteinShader.
   main(String[])   - 
Static method in class org.proteinshader.math. HermiteDemo 
 Creates a start and end point (each with a tangent vector)
    to plug into a Hermite object.
   main(String[])   - 
Static method in class org.proteinshader.math. LocalFrameDemo 
 Tests the constructors, setter methods, and multiply(Point3d)
    methods of the LocalFrame class.
   main(String[])   - 
Static method in class org.proteinshader.math. QuaternionDemo 
 Creates a QuaternionDemo object and uses it to test several
    operations on class Quaternion.
   main(String[])   - 
Static method in class org.proteinshader.math. SlerpDemo 
 Creates a SlerpDemo object and uses it to the SLERP algorithm.
   main(String[])   - 
Static method in class org.proteinshader.math. VectorAndPointDemo 
 Performs some simple tests (constructors, setXYZ, cross product,
    dot product, and normalization) with Vec3d objects, and a few
    tests with Point3d objects.
   main(String[])   - 
Static method in class org.proteinshader.structure.io.demos. PDBStructureReaderDemo 
 Uses a PDBStructureReader to read a PDB file and create a
    Structure, and then uses a WriterVisitor to write the contents
    of the Structure to an output file.
   main(String[])   - 
Static method in class org.proteinshader.structure.io.demos. SegmentDemo 
 Uses a PDBStructureReader to read a PDB file and create a
    Structure, and then uses a WriterVisitor to write the contents of
    the Structure to an output file.
   main(String[])   - 
Static method in class org.proteinshader.structure.io.enums. CategoryEnumDemo 
 Testing utility that prints all CategoryEnums in the order they
    are declared.
   main(String[])   - 
Static method in class org.proteinshader.structure.io.enums. RecordEnumDemo 
 Testing utility that prints all RecordEnums in the order they are
    declared.
   main(String[])   - 
Static method in class org.proteinshader.structure.visitor.demos. ModifierVisitorDemo 
 Uses a PDBStructureReader to read a PDB file and create a
    Structure, and then uses a WriterVisitor to write the contents
    of the Structure to an output file.
   main(String[])   - 
Static method in class org.proteinshader.structure.visitor.demos. VisitorDemo 
 Uses a StructureReader to read a PDB file and create a Structure,
    and then tests the ability of a Visitor to traverse the Structure.
   MainMenuBar   - Class in  org.proteinshader.gui.components.menubar  Provides the menu bar to place at the top of the ProteinShader
programs's main GUI window.   MainMenuBar(Mediator)   - 
Constructor for class org.proteinshader.gui.components.menubar. MainMenuBar 
 Constructs a ProteinShaderMenuBar.
   MAJOR_AXIS_SCALE   - 
Static variable in class org.proteinshader.graphics. FrenetFrames 
 The scale factor for major axes is 1.5.
   MAJOR_SPHERE_RADIUS   - 
Static variable in class org.proteinshader.graphics. FrenetFrames 
 The radius for spheres at major points is 0.3.
   MAJOR_SPHERE_TILING   - 
Static variable in class org.proteinshader.graphics. FrenetFrames 
 The number of slices and stacks for a major sphere is 30.
   MAP   - 
Static variable in class org.proteinshader.graphics.typography. TextLabelManager 
 The 'textlabel.frag' fragment shader uniform variable for a 2D
        texture map is named 'textLabelMap'.
   markAminoAcids(String, RegionEnum)   - 
Method in class org.proteinshader.structure. Region 
 Used to mark each AminoAcid with the ID and type of Region it
    belongs to.
   MAX_BEND   - 
Static variable in class org.proteinshader.structure. Segment 
 The maximum bend is the cosine of the smallest expected
        bend angle for a Segment.
   MAX_CA_DISTANCE   - 
Static variable in class org.proteinshader.structure.factory. SegmentFactory 
 The maximum distance from one alpha-carbon to the next
        alpha-carbon in a chain of amino acids should not be
        greater than 4.5 angstroms.
   MAX_CA_DISTANCE   - 
Static variable in class org.proteinshader.structure.visitor. FrenetFrameGeneratorVisitor 
 The maximum distance from one alpha-carbon to the next
        alpha-carbon in a chain of amino acids should not be
        greater than 4.5 angstroms.
   MAX_HEIGHT_FACTOR   - 
Static variable in class org.proteinshader.gui.components. SpringLoadedJFrame 
 The height factor will not be allowed to be more than 0.90.
   MAX_SLICES   - 
Static variable in class org.proteinshader.graphics.displaylists. CylinderReferences 
 The maximum number of slices for cylinders cached by this
        class is 14, while the minimum will be the MIN_SLICES
        declared in class Cylinder, which is 3.
   MAX_SPEED   - 
Static variable in class org.proteinshader.gui.components.controlpanel. MotionPanel 
 The maximum rotation speed in degrees per second is 90.
   MAX_TILING   - 
Static variable in class org.proteinshader.graphics.displaylists. SphereReferences 
 The maximum tiling number (slices and stacks) for spheres
        cached by this class is 57, while the minimum will be the
        MIN_TILING declared in class Sphere, which is 3.
   MAX_WIDTH_FACTOR   - 
Static variable in class org.proteinshader.gui.components. SpringLoadedJFrame 
 The width factor will not be allowed to be more than 0.90.
   Mediator   - Interface in  org.proteinshader.gui  This interface specifies the methods for the single central mediator
object that allows communication between objects of the gui, the data
structure, and the renderer.   MediatorImpl   - Class in  org.proteinshader.gui  Serves as the central mediator central object that allows
communication between objects of the gui, the data structure, and
the renderer.   MediatorImpl(ProteinShaderGUI, GLCanvas)   - 
Constructor for class org.proteinshader.gui. MediatorImpl 
 Constructs a ProteinShaderGUI.
   MENU_NAME   - 
Static variable in class org.proteinshader.gui.components.controlpanel. AntialiasingPanel 
 The menu name "Tiling" will be returned by toString().
   MENU_NAME   - 
Static variable in class org.proteinshader.gui.components.controlpanel. AtomColorPanel 
 The menu name "Color" will be returned by toString().
   MENU_NAME   - 
Static variable in class org.proteinshader.gui.components.controlpanel. AtomScalePanel 
 The menu name "Scale" will be returned by toString().
   MENU_NAME   - 
Static variable in class org.proteinshader.gui.components.controlpanel. AtomVisibilityPanel 
 The menu name "Atom Visibility" will be returned by
        the toString() method.
   MENU_NAME   - 
Static variable in class org.proteinshader.gui.components.controlpanel. CartoonColorPanel 
 The menu name "Color" will be returned by toString().
   MENU_NAME   - 
Static variable in class org.proteinshader.gui.components.controlpanel. CartoonSideChainsPanel 
 The menu name "Cartoon Side Chains" will be returned by
        the toString() method.
   MENU_NAME   - 
Static variable in class org.proteinshader.gui.components.controlpanel. CartoonVisibilityPanel 
 The menu name "Cartoon Visibility" will be returned by
        the toString() method.
   MENU_NAME   - 
Static variable in class org.proteinshader.gui.components.controlpanel. DecorationsPanel 
 The menu name "Decorations" will be returned by toString().
   MENU_NAME   - 
Static variable in class org.proteinshader.gui.components.controlpanel. MotionPanel 
 The menu name "Motion" will be returned by toString().
   MENU_NAME   - 
Static variable in class org.proteinshader.gui.components.controlpanel. OptimizePanel 
 The menu name "Tiling" will be returned by toString().
   MIN_CAP_TILING   - 
Static variable in class org.proteinshader.graphics. Cylinder 
 The minumum tiling (number of slices and stacks) for
        a capping sphere is 3.
   MIN_CHAR_IN_HEADER   - 
Static variable in class org.proteinshader.structure.io. PDBStructureReader 
 The minimum number of columns in a HEADER record.
   MIN_CHAR_PER_LINE   - 
Static variable in class org.proteinshader.structure.io. PDBLineParser 
 The minimum number of columns in a line from a PDB file.
   MIN_HEIGHT_FACTOR   - 
Static variable in class org.proteinshader.gui.components. SpringLoadedJFrame 
 The height factor will not be allowed to be less than 0.10.
   MIN_INITIAL_CAMERA_DISTANCE   - 
Static variable in class org.proteinshader.gui.viewing. Camera 
 The minimum initial camera distance is 20.
   MIN_SLICES   - 
Static variable in class org.proteinshader.graphics. Cylinder 
 The minimum number of slices for a cylinder is 3.
   MIN_SPEED   - 
Static variable in class org.proteinshader.gui.components.controlpanel. MotionPanel 
 The minimum rotation speed in degrees per second is -90.
   MIN_SPLASH_TIME   - 
Static variable in class org.proteinshader.gui. ProteinShaderSplasher 
  
   MIN_STACKS   - 
Static variable in class org.proteinshader.graphics. Cylinder 
 The minimum number of stacks for a cylinder is 1.
   MIN_THETA   - 
Static variable in class org.proteinshader.math. Quaternion 
 If theta is too close to zero for a SLERP calculation,
        then a clone of the start Quaternion will be returned.
   MIN_TILING   - 
Static variable in class org.proteinshader.graphics. Sphere 
 The minimum tiling is 3, and it is used to set the
        minimum number of slices and stacks for a sphere.
   MIN_WIDTH_FACTOR   - 
Static variable in class org.proteinshader.gui.components. SpringLoadedJFrame 
 The width factor will not be allowed to be less than 0.10.
   MINOR_AXIS_SCALE   - 
Static variable in class org.proteinshader.graphics. FrenetFrames 
 The scale factor for minor axes is 0.5.
   MINOR_SPHERE_RADIUS   - 
Static variable in class org.proteinshader.graphics. FrenetFrames 
 The radius for spheres at minor points is 0.0375.
   MINOR_SPHERE_TILING   - 
Static variable in class org.proteinshader.graphics. FrenetFrames 
 The number of slices and stacks for a minor sphere is 10.
   minus(Point3d)   - 
Method in class org.proteinshader.math. Point3d 
 Returns the direction vector obtained by subtracting the point
    given as an argument from the calling point.
   minus(Vec3d)   - 
Method in class org.proteinshader.math. Vec3d 
 Creates a new vector by subtracting the vector given as an
    argument from the calling vector (the existing vectors are
    NOT modified).
   minus(Drawable)   - 
Method in class org.proteinshader.structure. Drawable 
 Returns a vector created by subtracting the xyz-center of the
    Drawable given as an argument from the xyz-center of the calling
    Drawable object.
   minusFromMe(Vec3d)   - 
Method in class org.proteinshader.math. Vec3d 
 The x, y, and z values of the vector given as an argument are
    subtracted from the x, y, and z values of the calling vector.
   MissingAATypeException   - Exception in  org.proteinshader.structure.exceptions  Used to report that an AminoAcid did not have a type specified.   MissingAATypeException()   - 
Constructor for exception org.proteinshader.structure.exceptions. MissingAATypeException 
 Sets the default message to "ERROR: An amino acid must have a type
    specifed".
   MissingAATypeException(String)   - 
Constructor for exception org.proteinshader.structure.exceptions. MissingAATypeException 
 The message given this constructor can be retrieved using
    getMessage().
   MissingAtomTypeException   - Exception in  org.proteinshader.structure.exceptions  Used to report that an Atom did not have a type specified.   MissingAtomTypeException()   - 
Constructor for exception org.proteinshader.structure.exceptions. MissingAtomTypeException 
 Sets the default message to "ERROR: An atom type cannot be null
    or unknown".
   MissingAtomTypeException(String)   - 
Constructor for exception org.proteinshader.structure.exceptions. MissingAtomTypeException 
 The message given this constructor can be retrieved using
    getMessage().
   MissingHetNameException   - Exception in  org.proteinshader.structure.exceptions  Used to report that a heterogen name is missing.   MissingHetNameException()   - 
Constructor for exception org.proteinshader.structure.exceptions. MissingHetNameException 
 Sets the default message to "ERROR: A heterogen name is missing".
   MissingHetNameException(String)   - 
Constructor for exception org.proteinshader.structure.exceptions. MissingHetNameException 
 The message given this constructor can be retrieved using
    getMessage().
   Model   - Class in  org.proteinshader.structure  A Model serves as a container for one or more Chains.   Model(String, String)   - 
Constructor for class org.proteinshader.structure. Model 
 Constructs a Model with the requested modelID and structureID.
   ModifierPanel   - Class in  org.proteinshader.gui.components.controlpanel  Presents the user with a choice of several control panels for changing
color, visiblity, scale, motion, and other features of the display.   ModifierPanel(Mediator, Frame)   - 
Constructor for class org.proteinshader.gui.components.controlpanel. ModifierPanel 
 Constructs a ModifierPanel.
   ModifierPanelListenerFactory   - Class in  org.proteinshader.gui.listeners.controlpanel  Creates the listener for the control panel menu of the ModifierPanel.   ModifierPanelListenerFactory(ModifierPanel)   - 
Constructor for class org.proteinshader.gui.listeners.controlpanel. ModifierPanelListenerFactory 
 Constructs a ModifierPanelListenerFactory.
   ModifierVisitor   - Class in  org.proteinshader.structure.visitor  Knows how to modify multiple Drawable objects while traversing
the hierarchy of objects contained in a Structure.   ModifierVisitor()   - 
Constructor for class org.proteinshader.structure.visitor. ModifierVisitor 
 Constructs a ModifierVisitor.
   ModifierVisitorDemo   - Class in  org.proteinshader.structure.visitor.demos  Temporary class for testing the ModifierVisitor class.   ModifierVisitorDemo()   - 
Constructor for class org.proteinshader.structure.visitor.demos. ModifierVisitorDemo 
 Constructs a PDBStructureReaderDemo.
   modify(Structure)   - 
Method in class org.proteinshader.structure.visitor.demos. ModifierVisitorDemo 
 The Helices of the Structure are modified by changing their shape
    to CYLINDER and setting all of their Atoms and Bonds to INVISIBLE.
   modify(Atom)   - 
Method in class org.proteinshader.structure.visitor.modifiers. AtomModifier 
 Modifies the ball radius if requested, and then calls on modify()
    of the superclass, DrawableModifier, to apply any other requested
    modifications such as visibility, color, or radius for
    space-filling model.
   modify(Drawable)   - 
Method in class org.proteinshader.structure.visitor.modifiers. DrawableModifier 
 Modifies the visibility, RGBA color, and/or radius of the Drawable
    object given as an argument.
   modify(Segment)   - 
Method in class org.proteinshader.structure.visitor.modifiers. SegmentModifier 
 Sets the patterns texture, halftoning texture, Segment color, or
    decoration type as requested by SegmentModifier methods, and then
    calls on modify() of the DrawableModifier superclass.
   modifyGlobal(float, float, float)   - 
Method in class org.proteinshader.gui.components.controlpanel. AtomColorPanel 
 Prepares the AtomModifier to modify the RGB color of Atoms, and
    then calls on the modifyGlobal() method of the RadioPanel.
   modifyGlobal(VisibilityEnum, float)   - 
Method in class org.proteinshader.gui.components.controlpanel. AtomVisibilityPanel 
 This helper method for applyVisibility() sets up the the
    AtomModifier and then calls on modifyGlobal() method of the radio
    panel.
   modifyGlobal(float, float, float)   - 
Method in class org.proteinshader.gui.components.controlpanel. CartoonColorPanel 
 Prepares the SegmentModifier to modify the RGB color of Segments,
    and then calls on the modifyGlobal() method of the RadioPanel.
   modifyGlobal(VisibilityEnum, float)   - 
Method in class org.proteinshader.gui.components.controlpanel. CartoonVisibilityPanel 
 This helper method for applyVisibility() sets up the the
    SegmentModifier and then calls on modifyGlobal() method of the
    radio panel.
   modifyGlobal(float, float, float)   - 
Method in class org.proteinshader.gui.components.controlpanel. ColorPanel 
 Prepares a DrawableModifier to modify the RGB color of Drawable
    objects, and then calls on the modifyGlobal() method of the
    RadioPanel.
   modifyGlobal(AtomModifier, BondModifier, SegmentModifier)   - 
Method in class org.proteinshader.gui.components.controlpanel. ColorPanel 
 Passes the DrawableModifiers to the modifyGlobal() method of the
    RadioPanel.
   modifyGlobal(AtomModifier, BondModifier, SegmentModifier)   - 
Method in class org.proteinshader.gui.components.controlpanel. RadioPanel 
 Applies the DrawableModifiers to whatever item or items are
    currently selected in the "Global:" menu.
   modifyGlobal(VisibilityEnum, float)   - 
Method in class org.proteinshader.gui.components.controlpanel. VisibilityPanel 
 This helper method for applyVisibility() sets up whatever
    DrawableModifiers are needed and then calls on the modifyGlobal()
    method of the radio panel.
   modifyGlobal(AtomModifier, BondModifier, SegmentModifier)   - 
Method in class org.proteinshader.gui.components.controlpanel. VisibilityPanel 
 Passes the DrawableModifiers to the modifyGlobal() method of the
    RadioPanel.
   modifyGlobalToDefault1()   - 
Method in class org.proteinshader.gui.components.controlpanel. AtomColorPanel 
 Default 1 sets the RGB color of Atoms based on Atom type.
   modifyGlobalToDefault1()   - 
Method in class org.proteinshader.gui.components.controlpanel. CartoonColorPanel 
 Default 1 sets the RGB color of Segments based on Region type.
   modifyGlobalToDefault1()   - 
Method in class org.proteinshader.gui.components.controlpanel. ColorPanel 
 Prepares the DrawableModifier to set RGB colors to a default
    value, and then calls on the modifyGlobal() method of the
    RadioPanel.
   modifyGlobalToDefault2()   - 
Method in class org.proteinshader.gui.components.controlpanel. AtomColorPanel 
 Default 2 sets the RGB color of Atoms based on AminoAcid type.
   modifyGlobalToDefault2()   - 
Method in class org.proteinshader.gui.components.controlpanel. CartoonColorPanel 
 Default 2 sets the RGB color of Segments based on AminoAcid type.
   modifyGlobalToDefault2()   - 
Method in class org.proteinshader.gui.components.controlpanel. ColorPanel 
  
   modifyHelices(float, float, float)   - 
Method in class org.proteinshader.gui.components.controlpanel. AtomColorPanel 
 Prepares the the AtomModifier to modify the RGB color of Atoms,
    and then calls on the modifyHelices() method of the RadioPanel.
   modifyHelices(VisibilityEnum, float)   - 
Method in class org.proteinshader.gui.components.controlpanel. AtomVisibilityPanel 
 This helper method for applyVisibility() sets up an AtomModifier
    and a BondModifier, and then calls on modifyHelices() method
    of the radio panel.
   modifyHelices(float, float, float)   - 
Method in class org.proteinshader.gui.components.controlpanel. CartoonColorPanel 
 Prepares the the SegmentModifier to modify the RGB color of
    Segments, and then calls on the modifyHelices() method of the
    RadioPanel.
   modifyHelices(VisibilityEnum, float)   - 
Method in class org.proteinshader.gui.components.controlpanel. CartoonVisibilityPanel 
 This helper method for applyVisibility() sets up a SegmentModifier
    and then calls on modifyHelices() method of the radio panel.
   modifyHelices(float, float, float)   - 
Method in class org.proteinshader.gui.components.controlpanel. ColorPanel 
 Prepares a DrawableModifier to modify the RGB color of Drawable
    objects, and then calls on the modifyHelices() method of the
    RadioPanel.
   modifyHelices(AtomModifier, BondModifier, SegmentModifier)   - 
Method in class org.proteinshader.gui.components.controlpanel. ColorPanel 
 Passes the DrawableModifiers to the modifyHelices() method of the
    RadioPanel.
   modifyHelices(AtomModifier, BondModifier, SegmentModifier)   - 
Method in class org.proteinshader.gui.components.controlpanel. RadioPanel 
 Applies the DrawableModifiers to the Helix or Helices currently
    selected in the "Helices:" menu.
   modifyHelices(VisibilityEnum, float)   - 
Method in class org.proteinshader.gui.components.controlpanel. VisibilityPanel 
 This helper method for applyVisibility() sets up whatever
    DrawableModifiers are needed and then calls on the modifyHelices()
    method of the radio panel.
   modifyHelices(AtomModifier, BondModifier, SegmentModifier)   - 
Method in class org.proteinshader.gui.components.controlpanel. VisibilityPanel 
 Passes the DrawableModifiers to the modifyHelices() method of the
    RadioPanel.
   modifyHelicesToDefault1()   - 
Method in class org.proteinshader.gui.components.controlpanel. AtomColorPanel 
 Default 1 sets the RGB color of Atoms based on Atom type.
   modifyHelicesToDefault1()   - 
Method in class org.proteinshader.gui.components.controlpanel. CartoonColorPanel 
 Default 1 sets the RGB color of Segments based on Region type.
   modifyHelicesToDefault1()   - 
Method in class org.proteinshader.gui.components.controlpanel. ColorPanel 
 Prepares the DrawableModifier to set RGB colors to a default
    value, and then calls on the modifyHelices() method of the
    RadioPanel.
   modifyHelicesToDefault2()   - 
Method in class org.proteinshader.gui.components.controlpanel. AtomColorPanel 
 Default 2 sets the RGB color of Atoms based on AminoAcid type.
   modifyHelicesToDefault2()   - 
Method in class org.proteinshader.gui.components.controlpanel. CartoonColorPanel 
 Default 2 sets the RGB color of Segments based on AminoAcid type.
   modifyHelicesToDefault2()   - 
Method in class org.proteinshader.gui.components.controlpanel. ColorPanel 
  
   modifyLoops(float, float, float)   - 
Method in class org.proteinshader.gui.components.controlpanel. AtomColorPanel 
 Prepares the AtomModifier to modify the RGB color of Atoms,
    and then calls on the modifyLoops() method of the RadioPanel.
   modifyLoops(VisibilityEnum, float)   - 
Method in class org.proteinshader.gui.components.controlpanel. AtomVisibilityPanel 
 This helper method for applyVisibility() sets up an AtomModifier
    and then calls on modifyLoops() method of the radio panel.
   modifyLoops(float, float, float)   - 
Method in class org.proteinshader.gui.components.controlpanel. CartoonColorPanel 
 Prepares the SegmentModifier to modify the RGB color of Segments,
    and then calls on the modifyLoops() method of the RadioPanel.
   modifyLoops(VisibilityEnum, float)   - 
Method in class org.proteinshader.gui.components.controlpanel. CartoonVisibilityPanel 
 This helper method for applyVisibility() sets up a SegmentModifier
    and then calls on the modifyLoops() method of the radio panel.
   modifyLoops(float, float, float)   - 
Method in class org.proteinshader.gui.components.controlpanel. ColorPanel 
 Prepares a DrawableModifier to modify the RGB color of Drawable
    objects, and then calls on the modifyLoops() method of the
    RadioPanel.
   modifyLoops(AtomModifier, BondModifier, SegmentModifier)   - 
Method in class org.proteinshader.gui.components.controlpanel. ColorPanel 
 Passes the DrawableModifiers to the modifyLoops() method of the
    RadioPanel.
   modifyLoops(AtomModifier, BondModifier, SegmentModifier)   - 
Method in class org.proteinshader.gui.components.controlpanel. RadioPanel 
 Applies the DrawableModifiers to the Loop or Loops currently
    selected in the "Loops:" menu.
   modifyLoops(VisibilityEnum, float)   - 
Method in class org.proteinshader.gui.components.controlpanel. VisibilityPanel 
 This helper method for applyVisibility() sets up whatever
    DrawableModifiers are needed and then calls on the modifyLoops()
    method of the radio panel.
   modifyLoops(AtomModifier, BondModifier, SegmentModifier)   - 
Method in class org.proteinshader.gui.components.controlpanel. VisibilityPanel 
 Passes the DrawableModifiers to the modifyLoops() method of the
    RadioPanel.
   modifyLoopsToDefault1()   - 
Method in class org.proteinshader.gui.components.controlpanel. AtomColorPanel 
 Default 1 sets the RGB color of Atoms based on Atom type.
   modifyLoopsToDefault1()   - 
Method in class org.proteinshader.gui.components.controlpanel. CartoonColorPanel 
 Default 1 sets the RGB color of Segments based on Region type.
   modifyLoopsToDefault1()   - 
Method in class org.proteinshader.gui.components.controlpanel. ColorPanel 
 Prepares the DrawableModifier to set RGB colors to a default
    value, and then calls on the modifyLoops() method of the
    RadioPanel.
   modifyLoopsToDefault2()   - 
Method in class org.proteinshader.gui.components.controlpanel. AtomColorPanel 
 Default 2 sets the RGB color of Atoms based on AminoAcid type.
   modifyLoopsToDefault2()   - 
Method in class org.proteinshader.gui.components.controlpanel. CartoonColorPanel 
 Default 2 sets the RGB color of Segments based on AminoAcid type.
   modifyLoopsToDefault2()   - 
Method in class org.proteinshader.gui.components.controlpanel. ColorPanel 
  
   modifySelected(float, float, float)   - 
Method in class org.proteinshader.gui.components.controlpanel. AtomColorPanel 
 Prepares the AtomModifier to modify the RGB color of Atoms, and
    then calls on the modifySelected() method of the RadioPanel.
   modifySelected(VisibilityEnum, float)   - 
Method in class org.proteinshader.gui.components.controlpanel. AtomVisibilityPanel 
 This helper method for applyVisibility() sets up the AtomModifier
    and the BondModifier and then calls on the modifySelected() method
    of the radio panel.
   modifySelected(float, float, float)   - 
Method in class org.proteinshader.gui.components.controlpanel. CartoonColorPanel 
 Prepares the SegmentModifier to modify the RGB color of Segments,
    and then calls on the modifySelected() method of the RadioPanel.
   modifySelected(VisibilityEnum, float)   - 
Method in class org.proteinshader.gui.components.controlpanel. CartoonVisibilityPanel 
 This helper method for applyVisibility() sets up the
    SegmentModifier and then calls on the modifySelected() method
    of the radio panel.
   modifySelected(float, float, float)   - 
Method in class org.proteinshader.gui.components.controlpanel. ColorPanel 
 Prepares a DrawableModifier to modify the RGB color of Drawable
    objects, and then calls on the modifySelected() method of the
    RadioPanel.
   modifySelected(AtomModifier, BondModifier, SegmentModifier)   - 
Method in class org.proteinshader.gui.components.controlpanel. ColorPanel 
 Passes the DrawableModifiers to the modifySelected() method of the
    RadioPanel.
   modifySelected(AtomModifier, BondModifier, SegmentModifier)   - 
Method in class org.proteinshader.gui.components.controlpanel. RadioPanel 
 Applies the DrawableModifiers to whatever item or items are
    currently selected in the "Selected:" menu.
   modifySelected(VisibilityEnum, float)   - 
Method in class org.proteinshader.gui.components.controlpanel. VisibilityPanel 
 This helper method for applyVisibility() sets up whatever
    DrawableModifiers are needed and then calls on the
    modifySelected() method of the radio panel.
   modifySelected(AtomModifier, BondModifier, SegmentModifier)   - 
Method in class org.proteinshader.gui.components.controlpanel. VisibilityPanel 
 Passes the DrawableModifiers to the modifySelected() method of the
    RadioPanel.
   modifySelectedToDefault1()   - 
Method in class org.proteinshader.gui.components.controlpanel. AtomColorPanel 
 Default 1 sets the RGB color of Atoms based on Atom type.
   modifySelectedToDefault1()   - 
Method in class org.proteinshader.gui.components.controlpanel. CartoonColorPanel 
 Default 1 sets the RGB color of Segments based on Region type.
   modifySelectedToDefault1()   - 
Method in class org.proteinshader.gui.components.controlpanel. ColorPanel 
 Prepares the DrawableModifier to set RGB colors to a default
    value, and then calls on the modifySelected() method of the
    RadioPanel.
   modifySelectedToDefault2()   - 
Method in class org.proteinshader.gui.components.controlpanel. AtomColorPanel 
 Default 2 sets the RGB color of Atoms based on AminoAcid type.
   modifySelectedToDefault2()   - 
Method in class org.proteinshader.gui.components.controlpanel. CartoonColorPanel 
 Default 2 sets the RGB color of Segments based on AminoAcid type.
   modifySelectedToDefault2()   - 
Method in class org.proteinshader.gui.components.controlpanel. ColorPanel 
  
   modifyStrands(float, float, float)   - 
Method in class org.proteinshader.gui.components.controlpanel. AtomColorPanel 
 Prepares the AtomModifier to modify the RGB color of Atoms,
    and then calls on the modifyStrands() method of the RadioPanel.
   modifyStrands(VisibilityEnum, float)   - 
Method in class org.proteinshader.gui.components.controlpanel. AtomVisibilityPanel 
 This helper method for applyVisibility() sets up an AtomModifier
    and then calls on modifyStrands() method of the radio panel.
   modifyStrands(float, float, float)   - 
Method in class org.proteinshader.gui.components.controlpanel. CartoonColorPanel 
 Prepares the SegmentModifier to modify the RGB color of Segments,
    and then calls on the modifyStrands() method of the RadioPanel.
   modifyStrands(VisibilityEnum, float)   - 
Method in class org.proteinshader.gui.components.controlpanel. CartoonVisibilityPanel 
 This helper method for applyVisibility() sets up a SegmentModifier
    and then calls on the modifyStrands() method of the radio panel.
   modifyStrands(float, float, float)   - 
Method in class org.proteinshader.gui.components.controlpanel. ColorPanel 
 Prepares a DrawableModifier to modify the RGB color of Drawable
    objects, and then calls on the modifyStrands() method of the
    RadioPanel.
   modifyStrands(AtomModifier, BondModifier, SegmentModifier)   - 
Method in class org.proteinshader.gui.components.controlpanel. ColorPanel 
 Passes the DrawableModifiers to the modifyStrands() method of the
    RadioPanel.
   modifyStrands(AtomModifier, BondModifier, SegmentModifier)   - 
Method in class org.proteinshader.gui.components.controlpanel. RadioPanel 
 Applies the DrawableModifiers to the BetaStrand or BetaStrands
    currently selected in the "Strands:" menu.
   modifyStrands(VisibilityEnum, float)   - 
Method in class org.proteinshader.gui.components.controlpanel. VisibilityPanel 
 This helper method for applyVisibility() sets up whatever
    DrawableModifiers are needed and then calls on the modifyStrands()
    method of the radio panel.
   modifyStrands(AtomModifier, BondModifier, SegmentModifier)   - 
Method in class org.proteinshader.gui.components.controlpanel. VisibilityPanel 
 Passes the DrawableModifiers to the modifyStrands() method of the
    RadioPanel.
   modifyStrandsToDefault1()   - 
Method in class org.proteinshader.gui.components.controlpanel. AtomColorPanel 
 Default 1 sets the RGB color of Atoms based on Atom type.
   modifyStrandsToDefault1()   - 
Method in class org.proteinshader.gui.components.controlpanel. CartoonColorPanel 
 Default 1 sets the RGB color of Segments based on Region type.
   modifyStrandsToDefault1()   - 
Method in class org.proteinshader.gui.components.controlpanel. ColorPanel 
 Prepares the DrawableModifier to set RGB colors to a default
    value, and then calls on the modifyStrands() method of the
    RadioPanel.
   modifyStrandsToDefault2()   - 
Method in class org.proteinshader.gui.components.controlpanel. AtomColorPanel 
 Default 2 sets the RGB color of Atoms based on AminoAcid type.
   modifyStrandsToDefault2()   - 
Method in class org.proteinshader.gui.components.controlpanel. CartoonColorPanel 
 Default 2 sets the RGB color of Segments based on AminoAcid type.
   modifyStrandsToDefault2()   - 
Method in class org.proteinshader.gui.components.controlpanel. ColorPanel 
  
   MotionPanel   - Class in  org.proteinshader.gui.components.controlpanel  This control panel allows the user to rotate the image or move the
camera.   MotionPanel(Mediator, Frame)   - 
Constructor for class org.proteinshader.gui.components.controlpanel. MotionPanel 
 Constructs a MotionPanel.
   MotionPanelListenerFactory   - Class in  org.proteinshader.gui.listeners.controlpanel  Creates the listeners for the MotionPanel.   MotionPanelListenerFactory(Mediator, MotionPanel)   - 
Constructor for class org.proteinshader.gui.listeners.controlpanel. MotionPanelListenerFactory 
 Constructs a MotionPanelListenerFactory.
   mouseDragged(MouseEvent)   - 
Method in class org.proteinshader.gui.listeners. CanvasMouseListener 
 Rotates the model and redraws the canvas in response to the mouse
    being dragged across the canvas.
   mousePressed(MouseEvent)   - 
Method in class org.proteinshader.gui.listeners. CanvasMouseListener 
 Remembers xy-coordinates where mouse was clicked.
   mouseWheelMoved(MouseWheelEvent)   - 
Method in class org.proteinshader.gui.listeners. CanvasMouseListener 
 Zooms the camera in or out based on the number of wheel clicks.
   moveCameraXY(int, int)   - 
Method in class org.proteinshader.gui. Renderer 
 Moves the camera in the x and y directions.
   moveCameraZ(int)   - 
Method in class org.proteinshader.gui.listeners. CanvasMouseListener 
 Uses the mouse movement in the vertical direction to zoom the
    camera in or out.
   moveCameraZ(int)   - 
Method in class org.proteinshader.gui. Renderer 
 Moves the camera in the z-direction.
   moveX(int)   - 
Method in class org.proteinshader.gui.viewing. Camera 
 Add to the camera x position.
   moveY(int)   - 
Method in class org.proteinshader.gui.viewing. Camera 
 Add to the camera y position.
   moveZ(int)   - 
Method in class org.proteinshader.gui.viewing. Camera 
 Add to the camera z position.
   multiply(Point3d)   - 
Method in class org.proteinshader.math. LocalFrame 
 Multiplies the vertex by the local frame.
   multiply(Point3d)   - 
Method in class org.proteinshader.math. Quaternion 
 Multiplies a point by this quaternion and returns the resulting
    point.
   multiply(Vec3d)   - 
Method in class org.proteinshader.math. Quaternion 
 Multiplies a vector by this quaternion and returns the resulting
    vector.
   multiply(Quaternion)   - 
Method in class org.proteinshader.math. Quaternion 
 Creates a clone of the calling Quaternion and multiplies it by the
    Quaternion given as an argument.
   multiplyMe(Quaternion)   - 
Method in class org.proteinshader.math. Quaternion 
 Multiplies the calling Quaternion by the argument Quaternion and
    stores the result in the calling Quaternion.
 
 
    
 N  
 
   NamePair   - Class in  org.proteinshader.graphics.textures  Stores a menu name and a file name for a texture.   NamePair()   - 
Constructor for class org.proteinshader.graphics.textures. NamePair 
 Constructs a NamePair object.
   NamePair(String, String)   - 
Constructor for class org.proteinshader.graphics.textures. NamePair 
 Constructs a NamePair object.
   negate()   - 
Method in class org.proteinshader.math. Vec3d 
 Creates a new vector equal in magnitude but opposite in direction
    to the calling vector (the calling vector is NOT modified).
   negateMe()   - 
Method in class org.proteinshader.math. Vec3d 
 The direction of the calling vector is reversed by setting x to -x,
    y to -y, and z to -z.
   normalize()   - 
Method in class org.proteinshader.math. Quaternion 
 Creates and returns a new quaternion that is equivalent to the
    calling quaternion, but is guaranteed to be of unit length.
   normalize()   - 
Method in class org.proteinshader.math. Vec3d 
 Creates a new vector that has the same direction as the calling
    vector, but is of unit length (the calling vector is NOT
    modified).
   normalizeMe()   - 
Method in class org.proteinshader.math. Quaternion 
 Normalizes the calling quaternion object by dividing each
    component of the quaternion by the magnitude of the quaternion.
   normalizeMe()   - 
Method in class org.proteinshader.math. Vec3d 
 Normalizes the calling vector by determining its length
    (magnitude) and then dividing x, y, and z by the length.
   numberOfAminoAcids()   - 
Method in class org.proteinshader.structure. Chain 
 Returns the number of AminoAcids held by this Chain.
   numberOfAminoAcids()   - 
Method in class org.proteinshader.structure. Region 
 Returns the number of AminoAcids referred to by this Region.
   numberOfAtoms()   - 
Method in class org.proteinshader.structure. Residue 
 Returns the number of Atoms held by this Residue.
   numberOfBackboneAtoms()   - 
Method in class org.proteinshader.structure. AminoAcid 
 Returns the number of Atoms in the backbone (three if no atoms are
    missing in the structure).
   numberOfBetaStrands()   - 
Method in class org.proteinshader.structure. Chain 
 Returns the number of BetaStrands held by this Chain.
   numberOfBetaStrands()   - 
Method in class org.proteinshader.structure. Model 
 Returns the number of BetaStrands held by this Model.
   numberOfBonds()   - 
Method in class org.proteinshader.structure. Atom 
 Returns the number of Bonds held by this Atom.
   numberOfCategories()   - 
Method in class org.proteinshader.structure. Description 
 Returns the number of Category objects held by this Description.
   numberOfChains()   - 
Method in class org.proteinshader.structure. Model 
 Returns the number of Chains held by this Model.
   numberOfGlyphs()   - 
Method in class org.proteinshader.graphics.typography. GlyphSet 
 Returns the number of Glyphs in the set.
   numberOfHelices()   - 
Method in class org.proteinshader.structure. Chain 
 Returns the number of Helices held by this Chain.
   numberOfHelices()   - 
Method in class org.proteinshader.structure. Model 
 Returns the number of Helices held by this Model.
   numberOfHeterogens()   - 
Method in class org.proteinshader.structure. Chain 
 Returns the number of Heterogens held by this Chain.
   numberOfLines()   - 
Method in class org.proteinshader.structure. Record 
 Returns the number of lines in the Record.
   numberOfLoops()   - 
Method in class org.proteinshader.structure. Chain 
 Returns the number of Loops held by this Chain.
   numberOfLoops()   - 
Method in class org.proteinshader.structure. Model 
 Returns the number of Loops held by this Model.
   numberOfModels()   - 
Method in class org.proteinshader.structure. Structure 
 Returns the number of Models held by this Structure.
   numberOfRecords()   - 
Method in class org.proteinshader.structure. Category 
 Returns the number of Records held by this Category.
   numberOfSegments()   - 
Method in class org.proteinshader.structure. Region 
 Returns the number of Segments owned by this Region.
   numberOfSideChainAtoms()   - 
Method in class org.proteinshader.structure. AminoAcid 
 Returns the number of Atoms in the side chain.
   numberOfWaters()   - 
Method in class org.proteinshader.structure. Chain 
 Returns the number of Waters held by this Chain.
 
 
    
 O  
 
   ONE_DEGREE   - 
Static variable in class org.proteinshader.math. Quaternion 
 1 degree given in radians is PI / 180.
   OPEN   - 
Static variable in class org.proteinshader.gui.components.menubar. FileMenu 
 The OPEN keyboard command is
        KeyEvent.VK_O and ActionEvent.CTRL_MASK.
   openAboutBox()   - 
Method in class org.proteinshader.gui.components.menubar. HelpMenu 
 Opens an AboutBox with a message about this program.
   openAtomListDialog()   - 
Method in class org.proteinshader.gui.components.controlpanel. SelectorPanel 
 Sets the Atom list dialog to visible and disables the "Atoms"
    button.
   openColorChooser()   - 
Method in class org.proteinshader.gui.components.controlpanel. ColorPanel 
 Opens a JColorChooser in a dialog box.
   openColorChooser()   - 
Method in class org.proteinshader.gui.components.menubar. BackgroundMenu 
 Opens a JColorChooser in a dialog box.
   openControlFrame()   - 
Method in interface org.proteinshader.gui. Mediator 
 Opens the spring-loaded frame holding the control panel.
   openControlFrame()   - 
Method in class org.proteinshader.gui. MediatorImpl 
 Opens the spring-loaded frame holding the ControlPanel.
   openControlFrame()   - 
Method in class org.proteinshader.gui. ProteinShaderGUI 
 Opens the spring-loaded frame holding the control panel.
   openControlPanel()   - 
Static method in class org.proteinshader.gui. ProteinShader 
 Opens the retractable control panel on the right side of the ProteinShader
    GUI.
   openGraphicsCardInfoDialogBox()   - 
Method in class org.proteinshader.gui.components.menubar. HelpMenu 
 Opens a JOptionPane with a short summary of info on the graphics card.
   openOutputFile(String)   - 
Method in class org.proteinshader.structure.visitor. SFWriterVisitor 
 Opens a buffered writer using the filename given as an argument.
   openWebBrowserToLocalHelp()   - 
Method in class org.proteinshader.gui.components.menubar. HelpMenu 
 Opens the default web browser and loads the HELP_FILE html page in the help
    directory.
   openWebBrowserToSourceForgeSite()   - 
Method in class org.proteinshader.gui.components.menubar. AboutBox 
 Opens the default web browser to the HelpMenu.SOURCEFORGE_WEB_SITE.
   openWebBrowserToSourceForgeSite()   - 
Method in class org.proteinshader.gui.components.menubar. HelpMenu 
 Opens the default web browser to the HelpMenu.SOURCEFORGE_WEB_SITE.
   OptimizePanel   - Class in  org.proteinshader.gui.components.controlpanel  This control panel allows the user to optimize the display for image
quality or rendering speed.   OptimizePanel(Mediator, Frame)   - 
Constructor for class org.proteinshader.gui.components.controlpanel. OptimizePanel 
 Constructs an OptimizePanel.
   OptimizePanelListenerFactory   - Class in  org.proteinshader.gui.listeners.controlpanel  Creates the listener for the OptimizePanel.   OptimizePanelListenerFactory(Mediator, OptimizePanel)   - 
Constructor for class org.proteinshader.gui.listeners.controlpanel. OptimizePanelListenerFactory 
 Constructs a OptimizePanelListenerFactory.
   org.proteinshader.graphics   - package org.proteinshader.graphics Holds the drawing classes: Ribbon, Tube, FrenetFrames, Sphere, and Cylinder.   org.proteinshader.graphics.adapter   - package org.proteinshader.graphics.adapter Holds the StructureToGraphics class, which is used to manage the use of the
drawing classes and OpenGL display lists.   org.proteinshader.graphics.displaylists   - package org.proteinshader.graphics.displaylists Holds the classes needed to manage OpenGL display lists, which are used
to cache reusable geometry for spheres, cylinders, ribbon segments, and
tube segments.   org.proteinshader.graphics.enums   - package org.proteinshader.graphics.enums Holds the enums needed by org.proteinshader.graphics and its subpackages.   org.proteinshader.graphics.exceptions   - package org.proteinshader.graphics.exceptions Holds the exception classes needed by org.proteinshader.graphics and its 
subpackages.   org.proteinshader.graphics.shader   - package org.proteinshader.graphics.shader Holds the classes needed for compiling and managing the vertex and fragment 
shaders used for texture mapping and custom lighting calculations.   org.proteinshader.graphics.textures   - package org.proteinshader.graphics.textures Holds the classes needed for creating and managing OpenGL texture objects.   org.proteinshader.graphics.typography   - package org.proteinshader.graphics.typography Holds the classes needed for applying text labels to the curved surfaces of
tubes and ribbons.   org.proteinshader.graphics.utils   - package org.proteinshader.graphics.utils Holds a testing utility, class SegmentRenderer, which can be used for 
drawing ribbon and tube segments on-the-fly, rather than using cached 
geometry in the form of OpenGL display lists.   org.proteinshader.gui   - package org.proteinshader.gui Holds all of the Swing GUI components and their associated listeners, 
including class Renderer, which is registered as a listener for the GLCanvas
object that is used a drawing surface.   org.proteinshader.gui.components   - package org.proteinshader.gui.components Holds the gui components (JPanels and JMenus) needed by class ProteinShaderGUI.   org.proteinshader.gui.components.controlpanel   - package org.proteinshader.gui.components.controlpanel Holds the JPanels used in the right-side retractable control panel of the GUI.   org.proteinshader.gui.components.menubar   - package org.proteinshader.gui.components.menubar Holds the JMenus used in menu bar above the canvas.   org.proteinshader.gui.enums   - package org.proteinshader.gui.enums Holds the enums needed by org.proteinshader.gui and its subpackages.   org.proteinshader.gui.exceptions   - package org.proteinshader.gui.exceptions Holds the exception classes needed by org.proteinshader.gui and its subpackages.   org.proteinshader.gui.listeners   - package org.proteinshader.gui.listeners Holds the listener classes for the gui.   org.proteinshader.gui.listeners.controlpanel   - package org.proteinshader.gui.listeners.controlpanel Holds the listeners for components used in the JPanels of the right-side
retractable control panel of the GUI.   org.proteinshader.gui.listeners.menubar   - package org.proteinshader.gui.listeners.menubar Holds the listeners for the items used in the JMenus of the menu bar above 
the canvas.   org.proteinshader.gui.utils   - package org.proteinshader.gui.utils Holds minor utility classes needed by the Renderer.   org.proteinshader.gui.viewing   - package org.proteinshader.gui.viewing Holds important helper classes that the Renderer uses to control the view:
Camera, Rotation, Lighting,  etc .   org.proteinshader.math   - package org.proteinshader.math The key classes in this package are Hermite and Quaternion, which are needed
for generating the ribbons and tubes that are used to represent the backbone
of a protein in a cartoon-type display.   org.proteinshader.structure   - package org.proteinshader.structure Holds the classes that store information from a Protein Data Bank file:
Structure, Model, Chain, AminoAcid, Heterogen, Water, Atom, Bond, Helix, 
BetaStrand, Loop,  etc .   org.proteinshader.structure.enums   - package org.proteinshader.structure.enums Holds the enums needed by org.proteinshader.structure and its subpackages.   org.proteinshader.structure.exceptions   - package org.proteinshader.structure.exceptions Holds the exception classes needed by org.proteinshader.structure and its 
subpackages.   org.proteinshader.structure.factory   - package org.proteinshader.structure.factory Holds the factory classes needed for generating the Segment objects
that hold the information needed for rendering ribbon and tube segments.   org.proteinshader.structure.io   - package org.proteinshader.structure.io Holds the classes needed for reading Protein Data Bank structure files.   org.proteinshader.structure.io.demos   - package org.proteinshader.structure.io.demos Holds demo classes that were used for early testing of the io classes.   org.proteinshader.structure.io.enums   - package org.proteinshader.structure.io.enums Holds the enums needed for parsing Protein Data Bank structure files.   org.proteinshader.structure.io.exceptions   - package org.proteinshader.structure.io.exceptions Holds the exception classes that may be needed when parsing Protein Data
Bank structure files.   org.proteinshader.structure.io.filters   - package org.proteinshader.structure.io.filters Holds filters for checking file extensions.   org.proteinshader.structure.sort   - package org.proteinshader.structure.sort Holds the DrawableSorter class, which is needed for sorting Drawable objects
based on their distance from the camera.   org.proteinshader.structure.visitor   - package org.proteinshader.structure.visitor Holds Visitor classes that know how to traverse the hierarchy of objects
held by class Structure.   org.proteinshader.structure.visitor.demos   - package org.proteinshader.structure.visitor.demos Holds demo classes that were used for early testing of the Visitor classes.   org.proteinshader.structure.visitor.enums   - package org.proteinshader.structure.visitor.enums Holds enums needed by the Visitor classes.   org.proteinshader.structure.visitor.exceptions   - package org.proteinshader.structure.visitor.exceptions Holds exception classes needed by the Visitor classes.   org.proteinshader.structure.visitor.modifiers   - package org.proteinshader.structure.visitor.modifiers Holds helper classes for the ModifierVisitor class, which is used to modify
Drawable objects (Atoms, Bonds, and Segments).   OrientationEnum   - Enum in  org.proteinshader.gui.enums  Provides a general orientation enumeration.   OrientationMenu   - Class in  org.proteinshader.gui.components.menubar  Provides the Orientation menu for the MainMenuBar.   OrientationMenu(Mediator)   - 
Constructor for class org.proteinshader.gui.components.menubar. OrientationMenu 
 Constructs a OrientationMenu.
   OrientationMenuListenerFactory   - Class in  org.proteinshader.gui.listeners.menubar  Creates all event listeners needed by the OrientationMenu of the
MainMenuBar.   OrientationMenuListenerFactory(Mediator)   - 
Constructor for class org.proteinshader.gui.listeners.menubar. OrientationMenuListenerFactory 
 Constructs a OrientationMenuListenerFactory.
 
 
    
 P  
 
   PaddedListCellRenderer   - Class in  org.proteinshader.gui.utils  Adds a single blank space of padding in front of the text written in a
cell of a JList or a JComboBox.   PaddedListCellRenderer()   - 
Constructor for class org.proteinshader.gui.utils. PaddedListCellRenderer 
 Creates a PaddedListCellRenderer.
   parseLine(String, int)   - 
Method in class org.proteinshader.structure.io. PDBLineParser 
 Verifies that the line is a valid PDB record before adding it's
    data to the Structure.
   PATH   - 
Static variable in class org.proteinshader.structure.io.demos. PDBStructureReaderDemo 
  
   PATH   - 
Static variable in class org.proteinshader.structure.io.demos. SegmentDemo 
  
   PATH   - 
Static variable in class org.proteinshader.structure.visitor.demos. ModifierVisitorDemo 
  
   PATH   - 
Static variable in class org.proteinshader.structure.visitor.demos. VisitorDemo 
  
   PATTERNS_CONFIG   - 
Static variable in class org.proteinshader.graphics.textures. TextureFactory 
 Stores the name of the patterns configuration file,
        which is currently set to 'patterns_textures.conf'.
   PATTERNS_DIRECTORY   - 
Static variable in class org.proteinshader.graphics.textures. TextureFactory 
 Stores the relative pathname of the patterns textures
        directory.
   PDBAtomFieldExtractor   - Class in  org.proteinshader.structure.io  Extracts fields from a PDB ATOM or HETATM record.   PDBAtomFieldExtractor()   - 
Constructor for class org.proteinshader.structure.io. PDBAtomFieldExtractor 
 Constructs a PDBFieldExtractor.
   PDBBetaStrandFieldExtractor   - Class in  org.proteinshader.structure.io  Extracts fields from a PDB SHEET record.   PDBBetaStrandFieldExtractor()   - 
Constructor for class org.proteinshader.structure.io. PDBBetaStrandFieldExtractor 
 Constructs a PDBBetaStrandFieldExtractor.
   PDBConnectFieldExtractor   - Class in  org.proteinshader.structure.io  Extracts fields from a PDB CONECT record.   PDBConnectFieldExtractor()   - 
Constructor for class org.proteinshader.structure.io. PDBConnectFieldExtractor 
 Constructs a PDBFieldExtractor.
   PDBFieldExtractorException   - Exception in  org.proteinshader.structure.io.exceptions  Used to report that an error occurred while trying to extract a field
from a PDB record.   PDBFieldExtractorException()   - 
Constructor for exception org.proteinshader.structure.io.exceptions. PDBFieldExtractorException 
 Sets the default message to "An error occurred while extracting a
    field from a PDB record".
   PDBFieldExtractorException(String)   - 
Constructor for exception org.proteinshader.structure.io.exceptions. PDBFieldExtractorException 
 The message given this constructor can be retrieved using
    getMessage().
   PDBFileFilter   - Class in  org.proteinshader.structure.io.filters  Checks if a file has a ".pdb" file ending.   PDBFileFilter()   - 
Constructor for class org.proteinshader.structure.io.filters. PDBFileFilter 
 Constructs a PDBFileFilter.
   PDBHelixFieldExtractor   - Class in  org.proteinshader.structure.io  Extracts fields from a PDB HELIX record.   PDBHelixFieldExtractor()   - 
Constructor for class org.proteinshader.structure.io. PDBHelixFieldExtractor 
 Constructs a PDBHelixFieldExtractor.
   PDBLineParser   - Class in  org.proteinshader.structure.io  Parses a line from a PDB file and adds it to a Structure.   PDBLineParser(Structure)   - 
Constructor for class org.proteinshader.structure.io. PDBLineParser 
 Constructs a PDBLineParser for the Structure given as an argument.
   PDBLineParserException   - Exception in  org.proteinshader.structure.io.exceptions  Used to report that an error occurred while parsing a line from a PDB
file.   PDBLineParserException()   - 
Constructor for exception org.proteinshader.structure.io.exceptions. PDBLineParserException 
 Sets the default message to "An error occurred while parsing a
    line in a PDB file".
   PDBLineParserException(String)   - 
Constructor for exception org.proteinshader.structure.io.exceptions. PDBLineParserException 
 The message given this constructor can be retrieved using
    getMessage().
   PDBStructureReader   - Class in  org.proteinshader.structure.io  This concrete implementation of interface StructureReader reads a
protein structure entry from a '.pdb' file formated according to the
 
PDB Contents Guide   (Version 2.2, 20 Dec 1996) and creates a
Structure.   PDBStructureReader()   - 
Constructor for class org.proteinshader.structure.io. PDBStructureReader 
 Creates a PDBStructureReader.
   PDBStructureReaderDemo   - Class in  org.proteinshader.structure.io.demos  Temporary class for testing the PDBStructureReader class.   PDBStructureReaderDemo()   - 
Constructor for class org.proteinshader.structure.io.demos. PDBStructureReaderDemo 
 Constructs a PDBStructureReaderDemo.
   PI   - 
Static variable in class org.proteinshader.graphics. Shape 
 Pi is defined as 3.14159265358979.
   PI   - 
Static variable in class org.proteinshader.gui.viewing. Rotation 
 Pi is defined as 3.14159265358979, 
        and is equal to 180 degrees.
   PI   - 
Static variable in class org.proteinshader.structure.io. TubeVertices 
 Pi is defined as 3.14159265358979.
   Point3d   - Class in  org.proteinshader.math  This class is used to create a point with 3 elements of type double.   Point3d()   - 
Constructor for class org.proteinshader.math. Point3d 
 Constructs a Point3d at the origin.
   Point3d(double, double, double)   - 
Constructor for class org.proteinshader.math. Point3d 
 Constructs a point with the requested xyz-coordinates.
   printAngstroms(boolean)   - 
Method in class org.proteinshader.gui. Renderer 
 Setting print angstroms to true will cause object to camera distances to be 
    printed to standard out for testing and debugging purposes (and will also
    cause tiling numbers to be printed if automatic tiling is in use).
   printAutoTilingNumbers(boolean)   - 
Method in class org.proteinshader.graphics.adapter. StructureToGraphics 
 If automatic tiling is in use, setting this method to true will
    cause the tiling numbers to be printed to standard out for
    testing and debugging purposes.
   printAutoTilingNumbers(boolean)   - 
Method in class org.proteinshader.graphics.displaylists. CylinderReferences 
 If automatic tiling is in use, giving this method an argument of
    true will cause the tiling numbers to be printed to standard out
    for testing and debugging purposes.
   printAutoTilingNumbers(boolean)   - 
Method in class org.proteinshader.graphics.displaylists. SphereReferences 
 If automatic tiling is in use, giving this method an argument of
    true will cause the tiling numbers to be printed to standard out
    for testing and debugging purposes.
   printBadLines(List<String>)   - 
Method in class org.proteinshader.structure.io.demos. PDBStructureReaderDemo 
 Prints the bad lines from the PDB file to standard out.
   printBadLines(List<String>)   - 
Method in class org.proteinshader.structure.io.demos. SegmentDemo 
 Prints the bad lines from the PDB file to standard out.
   printBadLines(List<String>)   - 
Method in class org.proteinshader.structure.visitor.demos. ModifierVisitorDemo 
 Prints the bad lines from the PDB file to standard out.
   printBadLines(List<String>)   - 
Method in class org.proteinshader.structure.visitor.demos. VisitorDemo 
 Prints the bad lines from the PDB file.
   printBanner(String, String)   - 
Method in class org.proteinshader.math. LocalFrameDemo 
 Prints a banner with a row of asterisks above and below the title,
    and then a message right below the banner.
   printBanner(String, String)   - 
Method in class org.proteinshader.math. QuaternionDemo 
 Prints a banner with a row of asterisks above and below the title,
    and then a message right below the banner.
   printBanner(String, String)   - 
Method in class org.proteinshader.math. SlerpDemo 
 Prints a banner with a row of asterisks above and below the title,
    and then a message right below the banner.
   printColumnVectors(Vec3d, Vec3d, Vec3d)   - 
Method in class org.proteinshader.math. QuaternionDemo 
 Prints the 3 column vectors to standard out.
   printColumnVectors(Vec3d, Vec3d, Vec3d)   - 
Method in class org.proteinshader.math. SlerpDemo 
 Prints the 3 column vectors to standard out.
   printCurve(Hermite)   - 
Static method in class org.proteinshader.math. HermiteDemo 
 Uses the Hermite object to obtain and print interpolated points
    from parameter t = 0.0 to t = 1.0.
   printInterpolation(Quaternion, Quaternion, double[])   - 
Method in class org.proteinshader.math. QuaternionDemo 
 For each value in the array t, the slerp() method of class
    Quaternion will be used to calculate and print an interpolated
    Quaternion between the start and end Quaternions.
   printModelViewMatrix()   - 
Method in class org.proteinshader.gui.viewing. Camera 
 This debugging method prints the copy of the ModelViewMatrix
    stored n m_modelViewMatrix.
   printStartAndEndPoints(Point3d, Point3d, Vec3d, Vec3d)   - 
Static method in class org.proteinshader.math. HermiteDemo 
 Prints the start and end points along with their tangents.
   printTextLabel(TextLabel)   - 
Static method in class org.proteinshader.graphics.typography. GlyphDemo 
 Prints information from the TextLabel, including the bitmap.
   printXYZ()   - 
Method in class org.proteinshader.gui.viewing. Camera 
 Prints the xyz-coordinates for the camera.
   processID(String, String)   - 
Method in class org.proteinshader.structure. Atom 
 Returns the ID after trimming any leading or trailing whitespace.
   processID(String, String)   - 
Method in class org.proteinshader.structure. Chain 
 Returns the ID after trimming any leading or trailing whitespace.
   processID(String, String)   - 
Method in class org.proteinshader.structure. Heterogen 
 Overrides the processID() method of Residue in order to add
    guarantee that the residueID of a Heterogen always begins with
    the HETEROGEN_PREFIX.
   processID(String, String)   - 
Method in interface org.proteinshader.structure. IDTest 
 Returns the ID after trimming any leading or trailing whitespace.
   processID(String, String)   - 
Method in class org.proteinshader.structure. Model 
 Returns the Model ID after trimming any leading or trailing
    whitespace.
   processID(String, String)   - 
Method in class org.proteinshader.structure. Region 
 Returns the ID after trimming any leading or trailing whitespace.
   processID(String, String)   - 
Method in class org.proteinshader.structure. Residue 
 Returns the ID after trimming any leading or trailing whitespace.
   processID(String, String)   - 
Method in class org.proteinshader.structure. Structure 
 Returns the ID after trimming any leading or trailing whitespace.
   processSecondaryStructures()   - 
Method in class org.proteinshader.structure.io. PDBLineParser 
 Any Helix and BetaStrand records that were found while parsing
    lines will now be added to the Structure.
   ProteinShader   - Class in  org.proteinshader.gui  Opens the main GUI window for the ProteinShader program.   ProteinShader()   - 
Constructor for class org.proteinshader.gui. ProteinShader 
  
   ProteinShaderGUI   - Class in  org.proteinshader.gui  Provides the main GUI window for the ProteinShader program.   ProteinShaderGUI()   - 
Constructor for class org.proteinshader.gui. ProteinShaderGUI 
 Constructs a ProteinShaderGUI.
   ProteinShaderSplasher   - Class in  org.proteinshader.gui  Opens a splash screen, ProteinShaderLogo.png, while the ProteinShaderGUI is in
the process of starting up.   ProteinShaderSplasher()   - 
Constructor for class org.proteinshader.gui. ProteinShaderSplasher 
  
 
 
    
 Q  
 
   Quaternion   - Class in  org.proteinshader.math  This class is used to create a quaternion, a four-dimensional
complex number that is typically used to represent a rotation in
three-dimensional space.   Quaternion()   - 
Constructor for class org.proteinshader.math. Quaternion 
 Constructs the multiplication identity quaternion (x, y, z, w) =
    (0, 0, 0, 1).
   Quaternion(double, double, double, double)   - 
Constructor for class org.proteinshader.math. Quaternion 
 Constructs a quaternion.
   Quaternion(Vec3d, Vec3d, Vec3d)   - 
Constructor for class org.proteinshader.math. Quaternion 
 Creates a quaternion that is equivalent to the rotation contained
    in the 3 x 3 matrix [N B T].
   Quaternion(Vec3d, double)   - 
Constructor for class org.proteinshader.math. Quaternion 
 Creates a quaternion that is equivalent to the rotation specified
    by the axis and angle (in radians) given as aguments.
   QuaternionDemo   - Class in  org.proteinshader.math  Performs several tests on the Quaternion class, including a test
of the SLERP (Spherical Linear intERPolation) algorithm.   QuaternionDemo()   - 
Constructor for class org.proteinshader.math. QuaternionDemo 
 Creates a QuaternionDemo object with several vector and
    quaternions that can be used for testing purposes.
   QUIT   - 
Static variable in class org.proteinshader.gui.components.menubar. FileMenu 
 The QUIT keyboard command is
        KeyEvent.VK_Q and ActionEvent.CTRL_MASK.
 
 
    
 R  
 
   RADIANS_PER_DEGREE   - 
Static variable in class org.proteinshader.gui.viewing. Rotation 
 Pi divided by 180 degrees.
   RadioButtonEnum   - Enum in  org.proteinshader.gui.enums  Provides an enumeration of the types of radio buttons in the
RadioPanel class.   RadioPanel   - Class in  org.proteinshader.gui.components.controlpanel  Presents a common set of radio buttons and menus that are used by
other panels (color, visibility, and scale panels).   RadioPanel(Mediator, Frame, RadioPanel.Type)   - 
Constructor for class org.proteinshader.gui.components.controlpanel. RadioPanel 
 Constructs a RadioPanel.
   RadioPanel.Type   - Enum in  org.proteinshader.gui.components.controlpanel  There are two slightly different
        versions of the RadioPanel.   RadioPanelListenerFactory   - Class in  org.proteinshader.gui.listeners.controlpanel  Creates the listener for the  RadioPanel.   RadioPanelListenerFactory(RadioPanel)   - 
Constructor for class org.proteinshader.gui.listeners.controlpanel. RadioPanelListenerFactory 
 Constructs a RadioPanelListenerFactory.
   readConfigFile(String)   - 
Method in interface org.proteinshader.graphics.textures. ConfigReader 
 Reads a configuration file to obtain a list of menu names with a matching
    filename for each menu name.
   readConfigFile(File)   - 
Method in interface org.proteinshader.graphics.textures. ConfigReader 
 Reads a configuration file to obtain a list of menu names with a matching
    filename for each menu name.
   readConfigFile(String)   - 
Method in class org.proteinshader.graphics.textures. EqualSignConfigReader 
 Reads a configuration file to obtain a list of menu names with a matching
    filename for each menu name.
   readConfigFile(File)   - 
Method in class org.proteinshader.graphics.textures. EqualSignConfigReader 
 Reads a configuration file to obtain a list of menu names with a matching
    filename for each menu name.
   readConfigFile(String)   - 
Method in class org.proteinshader.graphics.typography. GlyphSetFactoryFactory 
 Reads the config file and stores its key-value pairs in a
    Properties object, which is a type of hash.
   readFontFile(GL)   - 
Method in class org.proteinshader.gui.utils. FpsLabel 
 Reads the fpsfont.glf file and stores an OpenGL texture object with the 
    characters to be used for printing frames per second on the canvas during 
    an animation.
   readGlyphSet()   - 
Method in class org.proteinshader.graphics.adapter. StructureToGraphics 
 Obtains a set of glyphs by using the default '.conf' configuration
    file in the fonts directory.
   readGlyphSet(Properties)   - 
Method in class org.proteinshader.graphics.typography. GLF2GlyphSetFactory 
 Reads information from a '.glf' file and constructs a GlyphSet
    object.
   readGlyphSet(Properties)   - 
Method in interface org.proteinshader.graphics.typography. GlyphSetFactory 
 Reads information from a source and constructs a GlyphSet object.
   readGlyphSet()   - 
Method in class org.proteinshader.graphics.typography. TextLabelFactory 
 Obtains a set of glyphs by using the default '.conf' configuration
    file in the fonts directory.
   readGlyphSet(String)   - 
Method in class org.proteinshader.graphics.typography. TextLabelFactory 
 Reads the config file given as an argument and use the info to
    create a GlyphSet.
   readGlyphSet()   - 
Method in class org.proteinshader.graphics.typography. TextLabelManager 
 Obtains a set of glyphs by using the default '.conf' configuration
    file in the fonts directory.
   readStructure(File)   - 
Method in interface org.proteinshader.gui. Mediator 
 Reads in a new Structure to display.
   readStructure(File)   - 
Method in class org.proteinshader.gui. MediatorImpl 
 Reads in a new Structure to display.
   readStructure(String)   - 
Method in class org.proteinshader.structure.io. PDBStructureReader 
 Reads a protein structure from a ".pdb" file and returns a
    Structure object.
   readStructure(File)   - 
Method in class org.proteinshader.structure.io. PDBStructureReader 
 Reads a protein structure from a ".pdb" file and returns a
    Structure object.
   readStructure(String)   - 
Method in interface org.proteinshader.structure.io. StructureReader 
 Reads a protein structure from a file and returns a Structure
    object.
   readStructure(File)   - 
Method in interface org.proteinshader.structure.io. StructureReader 
 Reads a protein structure from a file and returns a Structure
    object.
   readTextureFiles(GL)   - 
Method in class org.proteinshader.graphics.textures. TextureManager 
 Calls on the TextureFactory to read the texture files specified in
    the texture config file.
   Record   - Class in  org.proteinshader.structure  A Record object serves as a container for the lines of a PDB record.   Record(String, String)   - 
Constructor for class org.proteinshader.structure. Record 
 Constructs a Record with the name specified as an argument.
   RecordEnum   - Enum in  org.proteinshader.structure.io.enums  This file provides an enumeration of the record types defined in the
 
Protein Data Bank Content Guide  (Version 2.2, 20 Dec 1996).   RecordEnumDemo   - Class in  org.proteinshader.structure.io.enums  RecordEnumDemo is for testing RecordEnum.   RecordEnumDemo()   - 
Constructor for class org.proteinshader.structure.io.enums. RecordEnumDemo 
  
   redrawCanvas()   - 
Method in interface org.proteinshader.gui. Mediator 
 Redraws the canvas.
   redrawCanvas()   - 
Method in class org.proteinshader.gui. MediatorImpl 
 Redraws the canvas.
   redrawCanvas()   - 
Method in class org.proteinshader.gui. ProteinShaderGUI 
 Redraws the canvas.
   Region   - Class in  org.proteinshader.structure  Abstract class that contains references to a region of a Chain of
AminoAcids.   Region(String, String, Chain)   - 
Constructor for class org.proteinshader.structure. Region 
 Constructor for use by subclasses.
   RegionColorEnum   - Enum in  org.proteinshader.structure.enums  PrProvides default colors for regions of protein secondary structure
(alpha-helices, beta-strands and general loop regions).   RegionEnum   - Enum in  org.proteinshader.structure.enums  Provides a Region type enumeration (Loop, Helix,
or BetaStrand) useful for marking AminoAcids.   RegionMode   - Enum in  org.proteinshader.structure.visitor.enums  Provides an enumeration that can be used to control whether a Visitor
object traverses Regions (Helices or BetaStrands).   removeBond(Atom)   - 
Method in class org.proteinshader.structure. Atom 
 Removes the Bond from this Atom (the source) to the destination
    Atom.
   removeBond(Bond)   - 
Method in class org.proteinshader.structure. Atom 
 Removes the Bond if it exists.
   removeCategory(String)   - 
Method in class org.proteinshader.structure. Description 
 Removes a Category.
   removeLine(String)   - 
Method in class org.proteinshader.structure. Record 
 Removes the first occurrence of a line from the Record.
   removeRecord(String)   - 
Method in class org.proteinshader.structure. Category 
 Removes a Record.
   Renderer   - Class in  org.proteinshader.gui  Knows how to render a protein image onto a GLCanvas.   Renderer(Mediator)   - 
Constructor for class org.proteinshader.gui. Renderer 
 Constructs a Renderer.
   replaceLowerPanel(JPanel)   - 
Method in class org.proteinshader.gui.components.controlpanel. RadioPanel 
 Replaces the lower panel of the RadioPanel.
   resetDistance()   - 
Method in class org.proteinshader.gui.viewing. Camera 
 Sets the camera position based on the initial camera distance calculated 
    when a new Structure is loaded.
   reshape(GLAutoDrawable, int, int, int, int)   - 
Method in class org.proteinshader.gui. Renderer 
 Called on when the GLAutoDrawable object has changed its size so
    that the perspective and viewport can be recalculated.
   reshape(GL, int, int, int, int)   - 
Method in class org.proteinshader.gui.viewing. Camera 
 Called on when the GLAutoDrawable object has changed its size so
    that the perspective and viewport can be recalculated.
   Residue   - Class in  org.proteinshader.structure  This abstract class serves as a container for one or more Atoms.   Residue(String, String, String, String)   - 
Constructor for class org.proteinshader.structure. Residue 
 Constructor for use by subclasses.
   ResidueEnum   - Enum in  org.proteinshader.structure.enums  Provides an enumeration that can be used to restrict a Visitor to
traversing only a particular Residue type.   ResidueMode   - Enum in  org.proteinshader.structure.visitor.enums  Provides an enumeration that can be used to control the traversal
pattern of a Visitor object.   reverseXYAxesOfMiddleRotation()   - 
Method in class org.proteinshader.structure. Segment 
 Modifies the middle rotation quaternion such that if it is
    converted to the matrix [N B T], the N (x-axis) and B (y-axis)
    column vectors are inverted.
   Ribbon   - Class in  org.proteinshader.graphics  Draws a segment of a three-dimensional ribbon by using a
rectangular-shaped waist polygon.   Ribbon()   - 
Constructor for class org.proteinshader.graphics. Ribbon 
 Constructs a Ribbon using the default values for a LOOP.
   Ribbon(RegionEnum)   - 
Constructor for class org.proteinshader.graphics. Ribbon 
 Constructs a Ribbon using default values based on the Region type
    (Helix, BetaStrand, or Loop).
   Ribbon(double, double, double, double, boolean, double, double)   - 
Constructor for class org.proteinshader.graphics. Ribbon 
 Constructs a Ribbon.
   rotate(Point3d)   - 
Method in class org.proteinshader.math. LocalFrame 
 Returns a new point produced by rotating the point given as an
    argument by the quaternion held in this local frame.
   rotate(Vec3d)   - 
Method in class org.proteinshader.math. LocalFrame 
 Returns a new vector produced by rotating the vector given as an
    argument by the quaternion held in this local frame.
   rotateAboutXY(double, double)   - 
Method in class org.proteinshader.gui. Renderer 
 Rotates the model about the x-axis and y-axis.
   rotateAboutXY(double, double)   - 
Method in class org.proteinshader.gui.viewing. Rotation 
 Rotates the model about the x-axis and y-axis.
   rotateAboutZ(double)   - 
Method in class org.proteinshader.gui. Renderer 
 Rotates the model about the z-axis.
   rotateAboutZ(double)   - 
Method in class org.proteinshader.gui.viewing. Rotation 
 Rotates the model about the z-axis.
   rotateScaleAndCenterModel(GL)   - 
Method in class org.proteinshader.gui.viewing. Rotation 
 This helper method for display() rotates, scales, and centers the model.
   Rotation   - Class in  org.proteinshader.gui.viewing  Keeps track of rotation transformations of the current model.   Rotation(Mediator, FpsLabel)   - 
Constructor for class org.proteinshader.gui.viewing. Rotation 
 Constructs a Renderer.
 
 
    
 S  
 
   S_MAX   - 
Static variable in class org.proteinshader.graphics. Tube 
 The ending s-coordinate for texture mapping is 2.0,
        which will cause a texture image to wrap around the
        tube twice.
   S_MIN   - 
Static variable in class org.proteinshader.graphics. Tube 
 The starting s-coordinate for texture mapping is 0.0.
   scale(double)   - 
Method in class org.proteinshader.math. Vec3d 
 Creates a new vector by scaling the calling vector by the
    magnitude given as an argument (the calling vector is NOT
    modified).
   SCALE_INCREMENT   - 
Static variable in class org.proteinshader.gui.components.controlpanel. AtomScalePanel 
 The increment for the scale spinner is 0.1.
   SCALE_INIT   - 
Static variable in class org.proteinshader.gui.components.controlpanel. AtomScalePanel 
 The initial value for the scale spinner is 1.0.
   SCALE_MAX   - 
Static variable in class org.proteinshader.gui.components.controlpanel. AtomScalePanel 
 The maximum radius scale factor is 10.0.
   SCALE_MIN   - 
Static variable in class org.proteinshader.gui.components.controlpanel. AtomScalePanel 
 The minimum radius scale factor is 0.1.
   scaleBallRadius(double)   - 
Method in class org.proteinshader.structure. Atom 
 Scales the ball radius for use in stick-and-ball type models.
   scaleBallRadius(double)   - 
Method in class org.proteinshader.structure.visitor.modifiers. AtomModifier 
 Stores a memory that the ball radius (used in stick-and-ball
    models) needs to be multiplied by the scale factor given as an
    argument.
   scaleMe(double)   - 
Method in class org.proteinshader.math. Vec3d 
 Scales the calling vector by the value given as an argument.
   ScalePanelListenerFactory   - Class in  org.proteinshader.gui.listeners.controlpanel  Creates the listeners for the AtomScalePanel.   ScalePanelListenerFactory(Mediator, AtomScalePanel)   - 
Constructor for class org.proteinshader.gui.listeners.controlpanel. ScalePanelListenerFactory 
 Constructs a ScalePanelListenerFactory.
   scaleRadius(double)   - 
Method in class org.proteinshader.structure. Drawable 
 Multiplies the default radius by the scale factor given as an
    argument.
   scaleRadius(double)   - 
Method in class org.proteinshader.structure.visitor.modifiers. DrawableModifier 
 Stores a memory that the radius needs to be multiplied by the
    scale factor given as an argument.
   ScreenShot   - Class in  org.proteinshader.gui.viewing  Knows how to take a screen shot of the image on a GLCanvas and save it as a 
JPEG, PNG, or GIF file.   ScreenShot(ImageFormatEnum, File)   - 
Constructor for class org.proteinshader.gui.viewing. ScreenShot 
 Constructs a ScreenShot.
   ScreenShotException   - Exception in  org.proteinshader.gui.exceptions  Used to report an error that prevented a screen shot from being taken.   ScreenShotException(ImageFormatEnum, File, String, String)   - 
Constructor for exception org.proteinshader.gui.exceptions. ScreenShotException 
 The message given this constructor can be retrieved using
    getMessage().
   ScreenShotException(ImageFormatEnum, File, String)   - 
Constructor for exception org.proteinshader.gui.exceptions. ScreenShotException 
 The message given this constructor can be retrieved using
    getMessage().
   Segment   - Class in  org.proteinshader.structure  When a protein is drawn as a tube-like structure, a Segment is a
length of the tube that corresponds to an AminoAcid.   Segment(AminoAcid, Hermite, Hermite, Quaternion, Quaternion, Quaternion, boolean, boolean)   - 
Constructor for class org.proteinshader.structure. Segment 
 Creates a Segment.
   SEGMENT_LENGTH   - 
Static variable in class org.proteinshader.structure.factory. SegmentFactory 
 If an AminoAcid does not have an adjacent AminoAcid before
        or after it, then the Segment representing the AminoAcid will
        have it length determined by this constant, which currently
        is set at 2.6 angstroms in length.
   SegmentDemo   - Class in  org.proteinshader.structure.io.demos  Prints Segment data for troubleshooting.   SegmentDemo()   - 
Constructor for class org.proteinshader.structure.io.demos. SegmentDemo 
 Constructs a SegmentDemo.
   SegmentException   - Exception in  org.proteinshader.structure.exceptions  Used to report an error while trying to create a Segment object.   SegmentException()   - 
Constructor for exception org.proteinshader.structure.exceptions. SegmentException 
 Sets the default message to "ERROR: A Segment object could not be
    created".
   SegmentException(String)   - 
Constructor for exception org.proteinshader.structure.exceptions. SegmentException 
 The message given this constructor can be retrieved using
    getMessage().
   SegmentFactory   - Class in  org.proteinshader.structure.factory  Creates Segment objects for a Region (Loop, Helix, or BetaStrand).   SegmentFactory()   - 
Constructor for class org.proteinshader.structure.factory. SegmentFactory 
 No-arg constructor, which will be called automatically by concrete
    subclasses.
   SegmentListInfo   - Class in  org.proteinshader.graphics.displaylists  Stores information on an OpenGL display list for a Segment.   SegmentListInfo()   - 
Constructor for class org.proteinshader.graphics.displaylists. SegmentListInfo 
 Constructs a SegmentListInfo.
   SegmentListInfo(int, StyleEnum)   - 
Constructor for class org.proteinshader.graphics.displaylists. SegmentListInfo 
 Constructs a SegmentListInfo.
   SegmentModifier   - Class in  org.proteinshader.structure.visitor.modifiers  This helper class for the ModifierVisitor knows how to accept a
Segment object and modify its attributes.   SegmentModifier()   - 
Constructor for class org.proteinshader.structure.visitor.modifiers. SegmentModifier 
 Constructs an SegmentModifier.
   SegmentReferences   - Class in  org.proteinshader.graphics.displaylists  Stores information on multiple OpenGL display lists that can be used
for rendering Segments with different degrees of detail (this first
version only holds one OpenGL display list for each Segment, but a
future version will likely hold multiple OpenGL display lists for
each Segment (with varying tiling number) so that the level of detail
to be used can be calculated based on camera distance).   SegmentReferences()   - 
Constructor for class org.proteinshader.graphics.displaylists. SegmentReferences 
 Constructs a SegmentReferences object.
   SegmentRenderer   - Class in  org.proteinshader.graphics.utils  This class is intended only as a testing utility: it allows tube or ribbons 
segments to be drawn on-the-fly, rather than from OpenGL display lists, soley 
for the purpose of measure the performance advantage of caching geometry in 
OpenGL display lists.   SegmentRenderer()   - 
Constructor for class org.proteinshader.graphics.utils. SegmentRenderer 
 Constructs a SegmentReferences object.
   SEGMENTS   - 
Static variable in class org.proteinshader.math. HermiteDemo 
 The number of segments to divide the curve into is 10,
        so the total number of points printed will be 11,
        including the points for t = 0.0 and t = 1.0.
   SelectorPanel   - Class in  org.proteinshader.gui.components.controlpanel  Provides a panel with menus and lists for selecting a Structure's
Models, Chains, Residues, Atoms, Helices, and BetaStrands.   SelectorPanel(Mediator, Frame)   - 
Constructor for class org.proteinshader.gui.components.controlpanel. SelectorPanel 
 Constructs a SelectorPanel.
   SelectorPanelListenerFactory   - Class in  org.proteinshader.gui.listeners.controlpanel  Creates the listeners for the components of the SelectorPanel.   SelectorPanelListenerFactory(Mediator, SelectorPanel)   - 
Constructor for class org.proteinshader.gui.listeners.controlpanel. SelectorPanelListenerFactory 
 Constructs a SelectorPanelListenerFactory.
   SeparatorListCellRenderer   - Class in  org.proteinshader.gui.utils  Uses a Matte Border to add a separator (a dividing line) to the top of
a menu item.   SeparatorListCellRenderer(String[], Color)   - 
Constructor for class org.proteinshader.gui.utils. SeparatorListCellRenderer 
 Creates a SeparatorListCellRenderer that will add a separator (a
    dividing line) at the top of each menu item with a String matching
    one of the Strings given the constuctor.
   SERIAL_NO_CACHE_CAPACITY   - 
Static variable in class org.proteinshader.structure. Structure 
 Default starting capacity for Atom serial number cache.
   setAABondVisibility(VisibilityEnum)   - 
Method in class org.proteinshader.structure.visitor. BondGeneratorVisitor 
 Determines what visibility status the Bond generator will use when
    generating Bonds for AminoAcids.
   setAccPerspective(GLAutoDrawable, GL, double, double)   - 
Method in class org.proteinshader.gui.viewing. Camera 
 Sets the projection matrix by using the glFrustrum() method of OpenGL.
   setActiveRadioButton(RadioButtonEnum)   - 
Method in class org.proteinshader.gui.components.controlpanel. RadioPanel 
 Sets the RadioButtonEnum corresponding to the currently active
    radio button: SELECTED, HELICES, STRANDS, LOOPS, or GLOBAL.
   setActiveRadioButton(JComboBox)   - 
Method in class org.proteinshader.gui.components.controlpanel. RadioPanel 
 This method is called by the menu action listener in order to
    update the active radio button.
   setAllCheckBoxesSelected(boolean)   - 
Method in class org.proteinshader.gui.components.menubar. VisibilityMenu 
 Sets the selection state on all check box menu items.
   setAllStackNumbersToDefault()   - 
Method in class org.proteinshader.graphics.displaylists. SegmentReferences 
 Sets all stacks numbers to the defaults defined in classes Tube
    and Ribbon (for loops, alpha-helices, and beta-strands).
   setAllStackNumbersToDefault()   - 
Method in class org.proteinshader.graphics.utils. SegmentRenderer 
 Sets all stacks numbers to the defaults defined in classes Tube
    and Ribbon (for loops, alpha-helices, and beta-strands).
   setAlpha(float)   - 
Method in class org.proteinshader.structure. Drawable 
 Sets the alpha component of the RGBA color.
   setAlpha(float)   - 
Method in class org.proteinshader.structure.visitor.modifiers. DrawableModifier 
 Stores a memory that the alpha component of the RGBA color needs
    to be set to the value given as an argument.
   setAlphaRibbonStacks(int)   - 
Method in class org.proteinshader.graphics.displaylists. SegmentReferences 
 Sets the number of stacks for drawing an alpha-helix as a ribbon.
   setAlphaRibbonStacks(int)   - 
Method in class org.proteinshader.graphics.utils. SegmentRenderer 
 Sets the number of stacks for drawing an alpha-helix as a ribbon.
   setAlphaRibbonStacksToDefault()   - 
Method in class org.proteinshader.graphics.displaylists. SegmentReferences 
 Sets the number of stacks for drawing an alpha-helix as a ribbon
    to the default value defined in class Ribbon.
   setAlphaRibbonStacksToDefault()   - 
Method in class org.proteinshader.graphics.utils. SegmentRenderer 
 Sets the number of stacks for drawing an alpha-helix as a ribbon
    to the default value defined in class Ribbon.
   setAlphaToDefault()   - 
Method in class org.proteinshader.structure. Atom 
 Uses the Atom type to set the alpha component of the color to the
    default value specified in AtomEnum.
   setAlphaToDefault()   - 
Method in class org.proteinshader.structure. Drawable 
 Sets the alpha component of the RGBA color to DEFAULT_ALPHA.
   setAlphaToDefault()   - 
Method in class org.proteinshader.structure.visitor.modifiers. DrawableModifier 
 Stores a memory that the alpha value of the RGBA color needs to be
    set to its default.
   setAlphaTubeStacks(int)   - 
Method in class org.proteinshader.graphics.displaylists. SegmentReferences 
 Sets the number of stacks for drawing an alpha-helix as a tube.
   setAlphaTubeStacks(int)   - 
Method in class org.proteinshader.graphics.utils. SegmentRenderer 
 Sets the number of stacks for drawing an alpha-helix as a tube.
   setAlphaTubeStacksToDefault()   - 
Method in class org.proteinshader.graphics.displaylists. SegmentReferences 
 Sets the number of stacks for drawing an alpha-helix as a tube to
    the default value defined in class Tube.
   setAlphaTubeStacksToDefault()   - 
Method in class org.proteinshader.graphics.utils. SegmentRenderer 
 Sets the number of stacks for drawing an alpha-helix as a tube to
    the default value defined in class Tube.
   setAltAtom(Atom)   - 
Method in class org.proteinshader.structure. Atom 
 Sets the alternate Atom.
   setAminoAcidsCheckBoxSelected(boolean)   - 
Method in class org.proteinshader.gui.components.menubar. VisibilityMenu 
 Sets the selection state on the 'Amino Acids' check box menu item.
   setAminoAcidType(AminoAcidEnum)   - 
Method in class org.proteinshader.structure. Atom 
 Marks the Atom as belonging to a particular type of AminoAcid.
   setAngstromsPerPixel(double)   - 
Method in class org.proteinshader.structure. Drawable 
 The Camera class should use this method to set a conversion factor that
    will allow an x or y axis distance in viewport pixels be converted to an
    x or y distance in world coordinates (angstroms) at the current object to
    camera distance.
   setAtomList(Residue)   - 
Method in class org.proteinshader.gui.components.controlpanel. SelectorPanel 
 Sets the Atom list for a Residue.
   setAutoTiling(boolean)   - 
Method in class org.proteinshader.graphics.adapter. StructureToGraphics 
 If automatic tiling is set to true, then the level of detail
    (tiling number) for a sphere or cylinder will be calculated based
    on the camera distance for each Atom or Bond, respectively.
   setAutoTiling(boolean)   - 
Method in interface org.proteinshader.gui. Mediator 
 If automatic tiling is set to true, then the level of detail
    (tiling number) for a sphere or cylinder will be calculated based
    on the camera distance for each Atom or Bond, respectively.
   setAutoTiling(boolean)   - 
Method in class org.proteinshader.gui. MediatorImpl 
 If automatic tiling is set to true, then the level of detail
    (tiling number) for a sphere or cylinder will be calculated based
    on the camera distance for each Atom or Bond, respectively.
   setBackgroundColor(float, float, float)   - 
Method in interface org.proteinshader.gui. Mediator 
 Sets the background color for the canvas.
   setBackgroundColor(float, float, float)   - 
Method in class org.proteinshader.gui. MediatorImpl 
 Sets the background color for the canvas.
   setBackgroundColor(float, float, float)   - 
Method in class org.proteinshader.gui. Renderer 
 Sets the background color for the canvas.
   setBallRadius(double)   - 
Method in class org.proteinshader.structure. Atom 
 Sets the ball radius for use in stick-and-ball type models.
   setBallRadius(double)   - 
Method in class org.proteinshader.structure.visitor.modifiers. AtomModifier 
 Stores a memory that the ball radius needs to be set to the value
    given as an argument.
   setBallRadiusToDefault()   - 
Method in class org.proteinshader.structure. Atom 
 Uses the AtomEnum type to set the ball radius to a fraction of the
    covalent radius.
   setBallRadiusToDefault()   - 
Method in class org.proteinshader.structure.visitor.modifiers. AtomModifier 
 Stores a memory that the ball radius needs to be set to its
    default.
   setBaseCapped(boolean)   - 
Method in class org.proteinshader.graphics.displaylists. CylinderListInfo 
 Sets a boolean indicating whether the base of the cylinder is
    capped with a small sphere.
   setBaseRadius(double)   - 
Method in class org.proteinshader.graphics.displaylists. CylinderListInfo 
 Sets the base radius that was used to draw the cylinder in the display list.
   setBendTexture(int)   - 
Method in class org.proteinshader.structure. Segment 
 Sets the name (an integer) of an OpenGL texture object (stored on
    the graphics card) that can be used as a second texture for adding
    extra lines in the middle of segments that have a sharp bend to
    them.
   setBendTexture(int)   - 
Method in class org.proteinshader.structure.visitor.modifiers. SegmentModifier 
 Stores a memory that the Segment needs to have its OpenGL bend
    texture (an integer reference) set.
   setBetaRibbonStacks(int)   - 
Method in class org.proteinshader.graphics.displaylists. SegmentReferences 
 Sets the number of stacks for drawing a beta-strand as a ribbon.
   setBetaRibbonStacks(int)   - 
Method in class org.proteinshader.graphics.utils. SegmentRenderer 
 Sets the number of stacks for drawing a beta-strand as a ribbon.
   setBetaRibbonStacksToDefault()   - 
Method in class org.proteinshader.graphics.displaylists. SegmentReferences 
 Sets the number of stacks for drawing a beta-strand as a ribbon to
    the default value defined in class Ribbon.
   setBetaRibbonStacksToDefault()   - 
Method in class org.proteinshader.graphics.utils. SegmentRenderer 
 Sets the number of stacks for drawing a beta-strand as a ribbon to
    the default value defined in class Ribbon.
   setBetaTubeStacks(int)   - 
Method in class org.proteinshader.graphics.displaylists. SegmentReferences 
 Sets the number of stacks for drawing a beta-strand as a tube.
   setBetaTubeStacks(int)   - 
Method in class org.proteinshader.graphics.utils. SegmentRenderer 
 Sets the number of stacks for drawing a beta-strand as a tube.
   setBetaTubeStacksToDefault()   - 
Method in class org.proteinshader.graphics.displaylists. SegmentReferences 
 Sets the number of stacks for drawing a beta-strand as a tube to
    the default value defined in class Tube.
   setBetaTubeStacksToDefault()   - 
Method in class org.proteinshader.graphics.utils. SegmentRenderer 
 Sets the number of stacks for drawing a beta-strand as a tube to
    the default value defined in class Tube.
   setCameraDepth(double)   - 
Method in class org.proteinshader.structure. Drawable 
 The camera class should use this method to set a memory of the camera
    depth (z-coordinate of Drawable object in camera space).
   setCameraDistance(double)   - 
Method in class org.proteinshader.structure. Drawable 
 The camera class should use this method to set a memory of the distance
    between the xyz-center of this object and the camera.
   setCapEnd(boolean)   - 
Method in class org.proteinshader.structure. Segment 
 Sets a boolean value to remember if the end of the Segment should
    always be capped when it is drawn.
   setCapSlices(int)   - 
Method in class org.proteinshader.graphics.displaylists. CylinderListInfo 
 Sets the number of slices that were used to draw the capping
    sphere at one or both ends of the cylinder (if a capping sphere
    was used).
   setCapStacks(int)   - 
Method in class org.proteinshader.graphics.displaylists. CylinderListInfo 
 Sets the number of stacks that were used to draw the capping
    sphere at one or both ends of the cylinder (if a capping sphere
    was used).
   setCapStart(boolean)   - 
Method in class org.proteinshader.structure. Segment 
 Sets a boolean value to remember if the start of the Segment
    should be always be capped when it is drawn.
   setCenter(double, double, double)   - 
Method in class org.proteinshader.gui.viewing. Rotation 
 Sets the xyz-coordinates for the center of gravity of the current model.
   setCenterPanelComponentsEnabled(boolean)   - 
Method in class org.proteinshader.gui.components.controlpanel. OptimizePanel 
 Setting enabled to false will cause the center panel components
    to be grayed out (so they cannot be used).
   setChainMenu(Model)   - 
Method in class org.proteinshader.gui.components.controlpanel. SelectorPanel 
 Adds the Model's Chains to the Chain menu.
   setCharge(int)   - 
Method in class org.proteinshader.structure. Atom 
 Sets the electrical charge on the Atom.
   setColor(float, float, float)   - 
Method in class org.proteinshader.gui.components.controlpanel. AtomColorPanel 
 Clears the Atom modifier and sets the RGB colors.
   setColor(float, float, float)   - 
Method in class org.proteinshader.gui.components.controlpanel. CartoonColorPanel 
 Clears the SegmentModifier and sets the RGB colors.
   setColor(float, float, float, float)   - 
Method in class org.proteinshader.structure. Drawable 
 Sets the (red, green, blue, alpha ) components of the RGBA color.
   setColor(float, float, float)   - 
Method in class org.proteinshader.structure. Drawable 
 Sets the (red, green, blue) components of the RGBA color.
   setColor(float[])   - 
Method in class org.proteinshader.structure. Drawable 
 Sets the RGBA color by copying the values from the color array
    given as an argument.
   setColor(float, float, float)   - 
Method in class org.proteinshader.structure.visitor.modifiers. AtomModifier 
 Stores a memory that the (red, green, blue) components of the RGBA
    color need to be set to the values given as arguments.
   setColor(float, float, float, float)   - 
Method in class org.proteinshader.structure.visitor.modifiers. DrawableModifier 
 Stores a memory that the (red, green, blue, alpha) components of
    the RGBA color all need to be set to the values given as
    arguments.
   setColor(float, float, float)   - 
Method in class org.proteinshader.structure.visitor.modifiers. DrawableModifier 
 Stores a memory that the (red, green, blue) components of the RGBA
    color need to be set to the values given as arguments.
   setColor(float, float, float)   - 
Method in class org.proteinshader.structure.visitor.modifiers. SegmentModifier 
 Stores a memory that the (red, green, blue) components of the RGBA
    color need to be set to the values given as arguments.
   setColorToDefault()   - 
Method in class org.proteinshader.structure. Bond 
 Uses the Bond type to set the Drawable color to the default CPK
    color value specified in BondEnum.
   setControlPanelCheckBoxState(boolean)   - 
Method in class org.proteinshader.gui.components.menubar. ToolsMenu 
 Sets the selection state on the control panel check box menu item.
   setCurrentAtoms(Atom[])   - 
Method in interface org.proteinshader.gui. Mediator 
 Sets the current Atoms.
   setCurrentAtoms(Atom[])   - 
Method in class org.proteinshader.gui. MediatorImpl 
 Sets the current Atoms.
   setCurrentChain(Chain)   - 
Method in interface org.proteinshader.gui. Mediator 
 Sets the current Chain.
   setCurrentChain(Chain)   - 
Method in class org.proteinshader.gui. MediatorImpl 
 Sets the current Chain.
   setCurrentModel(Model)   - 
Method in interface org.proteinshader.gui. Mediator 
 Sets the current Model.
   setCurrentModel(Model)   - 
Method in class org.proteinshader.gui. MediatorImpl 
 Sets the current Model.
   setCurrentResidues(Residue[])   - 
Method in interface org.proteinshader.gui. Mediator 
 Sets the current Residues.
   setCurrentResidues(Residue[])   - 
Method in class org.proteinshader.gui. MediatorImpl 
 Sets the current Residues.
   setDebug(boolean)   - 
Method in class org.proteinshader.graphics.typography. GLF2GlyphSetFactory 
 Setting debug to true will activate several print statements that
    are useful for troubleshooting.
   setDebug(boolean)   - 
Method in interface org.proteinshader.graphics.typography. GlyphSetFactory 
 Setting debug to true will activate several print statements that
    are useful for troubleshooting.
   setDebug(boolean)   - 
Method in class org.proteinshader.graphics.typography. TextLabelFactory 
 Setting debug to true will turn on debugging (which prints data
    to standard out) for the GlyphSetFactory.
   setDebug(boolean)   - 
Method in class org.proteinshader.graphics.typography. TextLabelManager 
 Setting debug to true will turn on debugging (which prints data
    to standard out).
   setDebug(boolean)   - 
Method in class org.proteinshader.structure.visitor. Visitor 
 Sets debug to true or false.
   setDecoration(DecorationEnum)   - 
Method in class org.proteinshader.gui.components.controlpanel. DecorationsPanel 
 Sets up a SegmentModifier to change the decoration type and then
    passes the SegmentModifier to the RadioPanel.
   setDecoration(DecorationEnum)   - 
Method in class org.proteinshader.structure. Segment 
 Sets the decoration type for this Segment (PLAIN, WIREFRAME,
    TEXT_LABEL, or HALFTONING).
   setDecoration(DecorationEnum)   - 
Method in class org.proteinshader.structure.visitor.modifiers. SegmentModifier 
 Stores a memory that the decoration (PLAIN, WIREFRAME, TEXT_LABEL,
    or HALFTONING) needs to be set on the Segment.
   setDefaultRadioButtonAndMenu()   - 
Method in class org.proteinshader.gui.components.controlpanel. RadioPanel 
 Sets the default radio button and menu, which are currently the
    radio button labeled "Selected:" and the first menu item, which
    is "Model".
   setDefaultTextures()   - 
Method in class org.proteinshader.gui.components.controlpanel. DecorationsPanel 
 If the default decoration type for a Segment, Segment.DECORATION, is
    PATTERNS or HALFTONING, texture maps will be applied.
   setDimensions(double, double)   - 
Method in class org.proteinshader.graphics. Ribbon 
 Sets the width (x-axis dimension) and the height (y-axis
    dimension) of the rectangular waist polygon.
   setDisplayListName(int)   - 
Method in class org.proteinshader.graphics.displaylists. GeometricListInfo 
 Sets the name of the OpenGL display list that this GeometricListInfo object
    stores information on.
   setEndCapName(int)   - 
Method in class org.proteinshader.graphics.displaylists. SegmentListInfo 
 Sets the reference to the OpenGL display list for the end cap.
   setExtraLines(boolean)   - 
Method in class org.proteinshader.graphics.adapter. StructureToGraphics 
 If tubes or ribbons are being rendered, lines will be drawn at the
    beginning and end of each segment if extra lines is set to true.
   setExtraLines(boolean)   - 
Method in interface org.proteinshader.gui. Mediator 
 Controls whether extra lines (at the beginning and end of each
    segment of a tube or ribbon) will be shown when the display is
    in cartoon mode.
   setExtraLines(boolean)   - 
Method in class org.proteinshader.gui. MediatorImpl 
 Controls whether extra lines (at the beginning and end of each segment of a
    tube or ribbon) will be shown when the style is a cartoon type.
   setFile(File)   - 
Method in class org.proteinshader.graphics.textures. Texture 
 Sets the File with the complete pathname for the file the texture
    was read from.
   setFilename(String)   - 
Method in class org.proteinshader.graphics.textures. NamePair 
 Sets the name of the file that the texture is to be read from.
   setFilename(String)   - 
Method in class org.proteinshader.graphics.textures. Texture 
 Sets the name of the file that the texture was read from.
   setFPSTextureAndShader(GL, int)   - 
Method in class org.proteinshader.graphics.adapter. StructureToGraphics 
 Sets the texture object and shaders to be used when drawing an
    fps (frames per second) label on the canvas.
   setFragFile(File)   - 
Method in class org.proteinshader.graphics.shader. ShaderProgram 
 Sets the fragment shader source code File.
   setFragShaderName(int)   - 
Method in class org.proteinshader.graphics.shader. ShaderProgram 
 Sets the name (an integer) of the OpenGL fragment shader object.
   setFrenetFramesShaders(ShaderManager)   - 
Method in class org.proteinshader.graphics.displaylists. SegmentReferences 
 Makes the ShaderManager object available for use in the FrenetFrames
    object.
   setGluPerspective(GLAutoDrawable, GL)   - 
Method in class org.proteinshader.gui.viewing. Camera 
 Sets the projection matrix by using the gluPerspective() method of OpenGL.
   setHalftoningTexture(int)   - 
Method in class org.proteinshader.structure. Segment 
 Sets the name (an integer) of an OpenGL texture object (stored on
    the graphics card) that can be used for real-time halftoning with
    the Segment.
   setHalftoningTexture(int)   - 
Method in class org.proteinshader.structure.visitor.modifiers. SegmentModifier 
 Stores a memory that the Segment needs to have its OpenGL
    halftoning texture (an integer reference) set.
   setHeight(double)   - 
Method in class org.proteinshader.graphics.displaylists. CylinderListInfo 
 Sets the height that was used to draw the cylinder stored in the display 
    list.
   setHeightFactor(double)   - 
Method in class org.proteinshader.gui.components. SpringLoadedJFrame 
 Sets the height factor.
   setHeterogensCheckBoxSelected(boolean)   - 
Method in class org.proteinshader.gui.components.menubar. VisibilityMenu 
 Sets the selection state on the 'Heterogens' check box menu item.
   setImageOrientation(OrientationEnum)   - 
Method in interface org.proteinshader.gui. Mediator 
 Sets the image to the requested orientation (Front, Back, Left,
     etc .).
   setImageOrientation(OrientationEnum)   - 
Method in class org.proteinshader.gui. MediatorImpl 
 Sets the image to the requested orientation (Front, Back, Left,
     etc .).
   setImageOrientation(OrientationEnum)   - 
Method in class org.proteinshader.gui. Renderer 
 Resets the orientation of the image by resetting the angle of rotation 
    about the x-axis and the angle of rotation about the y-axis.
   setImageOrientation(OrientationEnum)   - 
Method in class org.proteinshader.gui.viewing. Rotation 
 Resets the orientation of the image by resetting the angle of rotation about
    the x-axis and the angle of rotation about the y-axis.
   setImageScale(double)   - 
Method in interface org.proteinshader.gui. Mediator 
 Sets the scale factor for the image.
   setImageScale(double)   - 
Method in class org.proteinshader.gui. MediatorImpl 
 Sets the scale factor for the image.
   setInitialCameraDistance(int)   - 
Method in class org.proteinshader.gui.viewing. Camera 
 Sets the initial camera distance on the z-axis.
   setListsToFill(List<Segment>, List<Drawable>, List<Drawable>)   - 
Method in class org.proteinshader.structure.visitor. VisibilityVisitor 
 Sets the lists to place opaque and translucent Drawable objects
    in.
   setLoopRibbonStacks(int)   - 
Method in class org.proteinshader.graphics.displaylists. SegmentReferences 
 Sets the number of stacks for drawing a loop as a ribbon.
   setLoopRibbonStacks(int)   - 
Method in class org.proteinshader.graphics.utils. SegmentRenderer 
 Sets the number of stacks for drawing a loop as a ribbon.
   setLoopRibbonStacksToDefault()   - 
Method in class org.proteinshader.graphics.displaylists. SegmentReferences 
 Sets the number of stacks for drawing a loop as a ribbon to
    the default value defined in class Ribbon.
   setLoopRibbonStacksToDefault()   - 
Method in class org.proteinshader.graphics.utils. SegmentRenderer 
 Sets the number of stacks for drawing a loop as a ribbon to
    the default value defined in class Ribbon.
   setLoopTubeStacks(int)   - 
Method in class org.proteinshader.graphics.displaylists. SegmentReferences 
 Sets the number of stacks for drawing a loop as a tube.
   setLoopTubeStacks(int)   - 
Method in class org.proteinshader.graphics.utils. SegmentRenderer 
 Sets the number of stacks for drawing a loop as a tube.
   setLoopTubeStacksToDefault()   - 
Method in class org.proteinshader.graphics.displaylists. SegmentReferences 
 Sets the number of stacks for drawing a loop as a tube to the
    default value defined in class Tube.
   setLoopTubeStacksToDefault()   - 
Method in class org.proteinshader.graphics.utils. SegmentRenderer 
 Sets the number of stacks for drawing a loop as a tube to the
    default value defined in class Tube.
   setMaterial(GL, float, float, float, float, float)   - 
Method in class org.proteinshader.graphics. Shape 
 Sets the material properties for the object about to be drawn.
   setMenuName(String)   - 
Method in class org.proteinshader.graphics.textures. NamePair 
 Sets a menu name for the texture.
   setMenuName(String)   - 
Method in class org.proteinshader.graphics.textures. Texture 
 Sets the menu name for the texture.
   setMinBondLength(double)   - 
Method in class org.proteinshader.structure.visitor. BondGeneratorVisitor 
 Sets the minimum Bond length value used for Bond prediction.
   setMinBondLength(double)   - 
Method in class org.proteinshader.structure.visitor. BondPredictor 
 Sets the minimum acceptable Bond length.
   setMinMaxXYZ(double, double, double, double, double, double)   - 
Method in class org.proteinshader.structure. Model 
 Sets the minimum and maximum values for the xyz-coordinates.
   setMode(RegionMode)   - 
Method in class org.proteinshader.structure.visitor. Visitor 
 Sets the traversal mode with regard to Regions: NO_REGIONS, LOOPS,
    HELICES, BETA_STRANDS, or ALL_REGIONS.
   setMode(ResidueMode)   - 
Method in class org.proteinshader.structure.visitor. Visitor 
 Sets the traversal mode with regard to what kind of Residues
    should be visited: ALL, AMINO_ACIDS, AA_AND_HETEROGENS,
    AA_AND_WATERS, HETEROGENS, WATERS, HET_AND_WATERS, or NONE.
   setMode(AAPortionMode)   - 
Method in class org.proteinshader.structure.visitor. Visitor 
 Sets the traversal mode with regard to what portion of each
    AminoAcid should be visited: ENTIRE_AA, BACKBONE, SIDE_CHAIN, N,
    CA, C, O, or OXT.
   setMode(AminoAcidEnum)   - 
Method in class org.proteinshader.structure.visitor. Visitor 
 Restricts the traversal to a particular AminoAcid type (this mode
    can also be used to restrict which Segments are visited).
   setMode(AtomEnum)   - 
Method in class org.proteinshader.structure.visitor. Visitor 
 Sets the traversal mode with regard to Atom type.
   setMode(String)   - 
Method in class org.proteinshader.structure.visitor. Visitor 
 Restricts the traversal to an atomID (as examples, all alpha
    carbons have the atomID of "CA", and all carbonyl oxygens have
    the atomID of "O").
   setModelMenu(Structure)   - 
Method in class org.proteinshader.gui.components.controlpanel. SelectorPanel 
 Adds the Structure's Models to the Model menu.
   setName(int)   - 
Method in class org.proteinshader.graphics.textures. Texture 
 Sets the OpenGL reference (an integer) for a texture object stored
    on the graphics card.
   setNewStructureFlag(boolean)   - 
Method in class org.proteinshader.gui. Renderer 
 Informs the Renderer if a new Structure has been loaded so that the camera
    distance can be reset before the image is shown.
   setNextSegment(Segment)   - 
Method in class org.proteinshader.structure. Segment 
 Stores a reference to the next Segment within the same Region.
   setPair(ShaderEnum)   - 
Method in class org.proteinshader.graphics.shader. ShaderProgram 
 Sets the type of shader pair (vertex and fragment pair).
   setPatternsTexture(int)   - 
Method in class org.proteinshader.structure. Segment 
 Sets the name (an integer) of an OpenGL texture object (stored on
    the graphics card) that can be used for applying a pattern to the
    surface of this Segment.
   setPatternsTexture(int)   - 
Method in class org.proteinshader.structure.visitor.modifiers. SegmentModifier 
 Stores a memory that the Segment needs to have its OpenGL patterns
    texture (an integer reference) set.
   setPercentField(float)   - 
Method in class org.proteinshader.gui.components.controlpanel. VisibilityPanel 
 Sets the value for the "% Translucent" text field.
   setPrevSegment(Segment)   - 
Method in class org.proteinshader.structure. Segment 
 Stores a reference to the previous Segment within the same Region.
   setProgramName(int)   - 
Method in class org.proteinshader.graphics.shader. ShaderProgram 
 Sets the name (an integer) of the OpenGL Shading Language
    "program" object.
   setRadioPanelSelectedMenu(String)   - 
Method in class org.proteinshader.gui.components.controlpanel. ControlPanel 
 Sets the "Selected:" menu in the RadioPanel (for both atom and cartoon
    modifier panels) of the ModiferPanel.
   setRadioPanelSelectedMenu(String)   - 
Method in class org.proteinshader.gui.components.controlpanel. ModifierPanel 
 Sets the "Selected:" menu in the RadioPanel (for both atom and cartoon
    modifier panels) to the item given as an argument.
   setRadioPanelSelectedMenu(String)   - 
Method in interface org.proteinshader.gui. Mediator 
 Sets the "Selected:" menu in the RadioPanel (for both atom and cartoon
    modifier panels) of the ModiferPanel.
   setRadioPanelSelectedMenu(String)   - 
Method in class org.proteinshader.gui. MediatorImpl 
 Sets the "Selected:" menu in the RadioPanel (for both atom and cartoon
    modifier panels) of the ModiferPanel.
   setRadius(double)   - 
Method in class org.proteinshader.graphics.displaylists. SphereListInfo 
 Sets the radius used to draw the sphere in the display list.
   setRadius(double)   - 
Method in class org.proteinshader.graphics. Tube 
 Sets the radius used for drawing the waist polygon, which
    approximates a circle as the number of vertices becomes large.
   setRadius(double)   - 
Method in class org.proteinshader.structure. Drawable 
 Sets the radius.
   setRadius(double)   - 
Method in class org.proteinshader.structure.io. TubeVertices 
 Sets the radius used for drawing the waist polygon, which
    approximates a circle as the number of vertices becomes large.
   setRadius(double)   - 
Method in class org.proteinshader.structure.visitor.modifiers. DrawableModifier 
 Stores a memory that the radius needs to be set to the value given
    as an argument.
   setRadiusToDefault()   - 
Method in class org.proteinshader.structure. Atom 
 Uses the AtomEnum type to set the radius to the van der Waals
    radius.
   setRadiusToDefault()   - 
Method in class org.proteinshader.structure. Bond 
 Uses the Bond type to set the radius to the default radius
    specified in BondEnum.
   setRadiusToDefault()   - 
Method in class org.proteinshader.structure. Drawable 
 Sets the radius to DEFAULT_RADIUS.
   setRadiusToDefault()   - 
Method in class org.proteinshader.structure.visitor.modifiers. DrawableModifier 
 Stores a memory that the radius needs to be set to its default.
   setRegionIDAndType(String, RegionEnum)   - 
Method in class org.proteinshader.structure. AminoAcid 
 Sets the regionID and the Region type.
   setResidueList(Chain)   - 
Method in class org.proteinshader.gui.components.controlpanel. SelectorPanel 
 Checks the currently selected Residue type (AminoAcids,
    Heterogens, or Waters) and copies them from the Chain to the
    Residue list.
   setRGBAToDefault()   - 
Method in class org.proteinshader.structure. Drawable 
 Sets the color by calling on setRGBToDefault() and
    setAlphaToDefault().
   setRGBAToDefault()   - 
Method in class org.proteinshader.structure.visitor.modifiers. DrawableModifier 
 Stores a memory that the (red, green, blue, alpha) components of
    the RGBA color all need to be set to their defaults.
   setRGBToDefault()   - 
Method in class org.proteinshader.structure. Atom 
 Uses the Atom type to set the RGB (red, green, blue) color
    components to the default CPK color value specified in AtomEnum.
   setRGBToDefault()   - 
Method in class org.proteinshader.structure. Drawable 
 Sets the (red, green, blue) components of the color to
    DEFAULT_RED, DEFAULT_GREEN, and DEFAULT_BLUE, respectively.
   setRGBToDefault()   - 
Method in class org.proteinshader.structure.visitor.modifiers. AtomModifier 
 Stores a memory that the (red, green, blue) components of the RGBA
    color need to be set to their default.
   setRGBToDefault()   - 
Method in class org.proteinshader.structure.visitor.modifiers. DrawableModifier 
 Stores a memory that the (red, green, blue) components of the RGBA
    color need to be set to their default.
   setRGBToDefault()   - 
Method in class org.proteinshader.structure.visitor.modifiers. SegmentModifier 
 Stores a memory that the (red, green, blue) components of the RGBA
    color need to be set to their default.
   setRotation(double, double, double, double)   - 
Method in class org.proteinshader.math. LocalFrame 
 Set the rotation to the quaternion xyzw-values given as arguments.
   setRotation(Quaternion)   - 
Method in class org.proteinshader.math. LocalFrame 
 Holds on to the Quaternion given as an argument so that it can be
    used as the rotation of the local coordinate frame.
   setRotation(Quaternion)   - 
Method in class org.proteinshader.structure. AminoAcid 
 Sets the rotation (the equivalent of a Frenet Frame) that will be
    needed for creating a Segment object based on this AminoAcid.
   setRotation(double, double, double, double)   - 
Method in class org.proteinshader.structure. AminoAcid 
 Sets the rotation (the equivalent of a Frenet Frame) that will be
    needed for creating a Segment object based on this AminoAcid.
   setScale(double)   - 
Method in class org.proteinshader.gui. Renderer 
 Sets the scale factor for the image.
   setScale(double)   - 
Method in class org.proteinshader.gui.viewing. Rotation 
 Sets the scale factor for the image.
   setSCoordParameters(double, double, boolean)   - 
Method in class org.proteinshader.graphics. Ribbon 
 Sets the lowest and highest s-coordinate for texture, and also
    sets the direction in which the s-texture coordinate increases.
   setSegment(Segment)   - 
Method in class org.proteinshader.structure. Atom 
 Sets the Segment the Atom is associated with.
   setSegment(Segment)   - 
Method in class org.proteinshader.structure. Bond 
 Sets the Segment the Bond is associated with.
   setSegmentAlpha(float)   - 
Method in class org.proteinshader.structure. Region 
 Sets the alpha component of the RGBA color for all Segments held
    by this Region.
   setSegmentAlphaToDefault()   - 
Method in class org.proteinshader.structure. Region 
 The defalult alpha value is 1.0.
   setSegmentRGB(float, float, float)   - 
Method in class org.proteinshader.structure. Region 
 Sets the RGB color on all Segments held by this Region.
   setSegmentRGBToDefault()   - 
Method in class org.proteinshader.structure. Region 
 The default RGB color is determined by the Region type.
   setSegmentsToAminoAcidColors()   - 
Method in class org.proteinshader.structure. Region 
 Sets the color of each Segment in the Region to the AAColorEnum
    for the AminoAcid the Segment corresponds to.
   setSegmentsToRegionColor()   - 
Method in class org.proteinshader.structure. Region 
 Sets the color of each Segment in the Region to the default color
    for the type of Region the Segment belongs to.
   setSelectedMenu(String)   - 
Method in class org.proteinshader.gui.components.controlpanel. RadioPanel 
 Sets the "Selected:" menu to the item given as an argument.
   setSelectionToDefaultSubpanel()   - 
Method in class org.proteinshader.gui.components.controlpanel. ModifierPanel 
 Sets the subpanel menu to the default subpanel.
   setShaders(ShaderManager)   - 
Method in class org.proteinshader.graphics. FrenetFrames 
 The ShaderManager is only needed because of an apparent bug on some 
    Mac OS X machines where material color can only be set once after a 
    custom shader is set, so to draw Frenet frames with more than one
    color, the shaders must be reset each time.
   setShape(HelixShapeEnum)   - 
Method in class org.proteinshader.structure. Helix 
 Sets the shape that should be used to represent the Helix.
   setShapeToDefault()   - 
Method in class org.proteinshader.structure. Helix 
 Sets the shape the default value, DEFAULT_SHAPE.
   setSideChainStyle(SideChainEnum)   - 
Method in class org.proteinshader.gui.components.controlpanel. CartoonSideChainsPanel 
 Applies the requested side chain style (NONE, SPACE_FILLING,
    BALLS_AND_STICKS, or STICKS) to whatever item or items are currently
    selected in the RadioPanel.
   setSideChainStyle(SideChainEnum, boolean)   - 
Method in class org.proteinshader.structure. Segment 
 Sets the side chain style (NONE, SPACE_FILLING, STICK_AND_BALLS,
    or STICKS) and determines whether the alpha-carbon should be
    shown.
   setSideChainStyle(SideChainEnum, boolean)   - 
Method in class org.proteinshader.structure.visitor.modifiers. SegmentModifier 
 Stores a memory that the side chain display needs to be set.
   setSlices(int)   - 
Method in class org.proteinshader.graphics.displaylists. CylinderListInfo 
 Sets the number of slices that were used to draw the cylinder
    stored in thedisplay list.
   setSlices(int)   - 
Method in class org.proteinshader.graphics.displaylists. SphereListInfo 
 Sets the number of slices that were used to draw the sphere stored
    in the display list.
   setSlices(int)   - 
Method in class org.proteinshader.structure.io. TubeVertices 
 Sets the number of slices that the nearly circular waist polygon
    would be divided into if a line was drawn from the center of the
    polygon to each vertex.
   setSlicesAndSCoordParameters(int, float, float, boolean)   - 
Method in class org.proteinshader.graphics. Tube 
 Sets the direction in which the s-texture coordinate increases
    from sCoordStart to sCoordEnd and then calls on
    setSlicesAndSCoordRange().
   setSlicesAndSCoordRange(int, float, float)   - 
Method in class org.proteinshader.graphics. Tube 
 Sets the number of slices that the nearly circular waist polygon
    would be divided into if a line was drawn from the center of the
    polygon to each vertex.
   setSliderValue(int)   - 
Method in class org.proteinshader.gui.components.controlpanel. VisibilityPanel 
 Sets the current value for the slider.
   setSpecularExp(float)   - 
Method in class org.proteinshader.structure. Drawable 
 Sets the specular exponent to be used in lighting calculations.
   setSpecularExp(float)   - 
Method in class org.proteinshader.structure.visitor.modifiers. DrawableModifier 
 Stores a memory that the specular exponent needs to be set to the
    value given as an argument.
   setSpecularExpToDefault()   - 
Method in class org.proteinshader.structure. Drawable 
 Sets the specular exponent to the default value,
    DEFAULT_SPECULAR_EXP.
   setSpecularExpToDefault()   - 
Method in class org.proteinshader.structure.visitor.modifiers. DrawableModifier 
 Stores a memory that the specular exponent needs to be set to its
    default.
   setSpinnerToCylinderCapValue()   - 
Method in class org.proteinshader.gui.components.controlpanel. OptimizePanel 
 Sets the value in the tiling spinner to the value displayed under
    the radio button for cylinder caps.
   setSpinnerToCylinderValue()   - 
Method in class org.proteinshader.gui.components.controlpanel. OptimizePanel 
 Sets the value in the tiling spinner to the value displayed under
    the radio button for cylinders.
   setSpinnerToSphereValue()   - 
Method in class org.proteinshader.gui.components.controlpanel. OptimizePanel 
 Sets the value in the tiling spinner to the value displayed under
    the radio button for spheres.
   setStacks(int)   - 
Method in class org.proteinshader.graphics.displaylists. CylinderListInfo 
 Sets the number of stacks that were used to draw the cylinder in
    the display list.
   setStacks(int)   - 
Method in class org.proteinshader.graphics.displaylists. SphereListInfo 
 Sets the number of stacks that were used to draw the sphere in the
    display list.
   setStartCapName(int)   - 
Method in class org.proteinshader.graphics.displaylists. SegmentListInfo 
 Sets the reference to the OpenGL display list for the start cap.
   setStyle(StyleEnum)   - 
Method in class org.proteinshader.graphics.adapter. StructureToGraphics 
 Sets the style (TUBES, RIBBONS, RIBBONS_AND_TUBES, FRENET_FRAMES, 
    SPACE_FILLING, BALLS_AND_STICKS, or STICKS).
   setStyle(StyleEnum)   - 
Method in class org.proteinshader.graphics.displaylists. GeometricListInfo 
 Sets the style that this geometric object is intended for: SPACE_FILLING, 
    BALLS_AND_STICKS, or STICKS.
   setStyle(StyleEnum)   - 
Method in interface org.proteinshader.gui. Mediator 
 Sets the current style (TUBES, RIBBONS, RIBBONS_AND_TUBES, FRENET_FRAMES,
    SPACE_FILLING, BALLS_AND_STICKS, or STICKS).
   setStyle(StyleEnum)   - 
Method in class org.proteinshader.gui. MediatorImpl 
 Sets the current style (TUBES, RIBBONS, RIBBONS_AND_TUBES, FRENET_FRAMES,
    SPACE_FILLING, BALLS_AND_STICKS, or STICKS).
   setThinSideMinAndMaxS(double, double)   - 
Method in class org.proteinshader.graphics. Ribbon 
 Sets the minimum and maximum values for the texture s-coordinate
    for the thin sides of the ribbon.
   setThinSidesName(int)   - 
Method in class org.proteinshader.graphics.displaylists. SegmentListInfo 
 Sets the reference to the OpenGL display list for the thin sides
    of a Ribbon.
   setToAminoAcidColor()   - 
Method in class org.proteinshader.structure. Atom 
 Sets the color of this Atom to the color of the AminoAcid it
    belongs to (if any).
   setToAminoAcidColor()   - 
Method in class org.proteinshader.structure. Segment 
 Sets the Segment color to the AAColorEnum for the AminoAcid this
    Segment corresponds to.
   setToAminoAcidColor()   - 
Method in class org.proteinshader.structure.visitor.modifiers. AtomModifier 
 Stores a memory that the Atom should be set to the default
    color for the AminoAcid that it belongs to.
   setToAminoAcidColor()   - 
Method in class org.proteinshader.structure.visitor.modifiers. SegmentModifier 
 Stores a memory that the Segment should be set to the default
    color for the AminoAcid that it represents.
   setToDefault()   - 
Method in class org.proteinshader.gui.components.menubar. StyleMenu 
 Sets the "Style" menu to the default INITIAL_STYLE.
   setTolerance(double)   - 
Method in class org.proteinshader.structure.visitor. BondGeneratorVisitor 
 Sets the tolerance value used for Bond prediction.
   setTolerance(double)   - 
Method in class org.proteinshader.structure.visitor. BondPredictor 
 Sets the Bond length tolerance.
   setTopCapped(boolean)   - 
Method in class org.proteinshader.graphics.displaylists. CylinderListInfo 
 Sets a boolean indicating whether the top of the cylinder is
    capped with a small sphere.
   setTopRadius(double)   - 
Method in class org.proteinshader.graphics.displaylists. CylinderListInfo 
 Sets the top radius that was used to draw the cylinder in the display list.
   setToRegionColor()   - 
Method in class org.proteinshader.structure. Segment 
 Sets the Segment color to the RegionColorEnum for the type of
    Region this Segment belongs to.
   setToRegionColor()   - 
Method in class org.proteinshader.structure.visitor.modifiers. SegmentModifier 
 Stores a memory that the Segment should be set to the default
    color for the Region that it belongs to.
   setTranslation(double, double, double)   - 
Method in class org.proteinshader.math. LocalFrame 
 Set the translation to the xyz-values given as arguments.
   setTranslation(Vec3d)   - 
Method in class org.proteinshader.math. LocalFrame 
 Holds on the the vector given as an argument so that it can be
    used as the translation for the local coordinate frame.
   setType(BondEnum)   - 
Method in class org.proteinshader.structure. Bond 
 Sets the Bond's type as an enum (SINGLE, DOUBLE, PEPTIDE,
     etc. ).
   setType(HelixEnum)   - 
Method in class org.proteinshader.structure. Helix 
 Sets the type of Helix.
   setVertFile(File)   - 
Method in class org.proteinshader.graphics.shader. ShaderProgram 
 Sets the vertex shader source code File.
   setVertShaderName(int)   - 
Method in class org.proteinshader.graphics.shader. ShaderProgram 
 Sets the name (an integer) of the OpenGL vertex shader object.
   setVisibility(VisibilityEnum)   - 
Method in class org.proteinshader.structure. Drawable 
 Sets the visibility status of the Drawable object.
   setVisibility(VisibilityEnum)   - 
Method in class org.proteinshader.structure.visitor.modifiers. DrawableModifier 
 Stores a memory that the visibility status needs to be set to the
    value given as an argument.
   setVisible(boolean, boolean)   - 
Method in class org.proteinshader.gui.components. SpringLoadedJFrame 
 If animate is true, a timer is used to gradually open or close the
    frame.
   setVisibleDrawables(Model, StyleEnum)   - 
Method in class org.proteinshader.gui. Renderer 
 An array of opaque Segments, and array of opaque Atoms and Bonds, and an 
    array of translucent Drawable objects will be obtained from the Model.
   setVisitSegments(boolean)   - 
Method in class org.proteinshader.structure.visitor. Visitor 
 Sets a boolean to indicate that Segments should be visited
   setWaterBondVisibility(VisibilityEnum)   - 
Method in class org.proteinshader.structure.visitor. BondGeneratorVisitor 
 Determines what visibility status the Bond generator use when
    generating Bonds for Waters.
   setWatersCheckBoxSelected(boolean)   - 
Method in class org.proteinshader.gui.components.menubar. VisibilityMenu 
 Sets the selection state on the 'Waters' check box menu item.
   setWidthFactor(double)   - 
Method in class org.proteinshader.gui.components. SpringLoadedJFrame 
 Sets the width factor.
   setXYZ(double, double, double)   - 
Method in class org.proteinshader.math. Point3d 
 Sets the xyz-values of the point.
   setXYZ(double, double, double)   - 
Method in class org.proteinshader.math. Vec3d 
 Sets the xyz-values of the vector.
   setXYZ(Vec3d)   - 
Method in class org.proteinshader.math. Vec3d 
 Sets the xyz-values of the vector to the xyz-values of the vector
    given as an argument.
   setXYZW(double, double, double, double)   - 
Method in class org.proteinshader.math. Quaternion 
 Sets the xyzw-values of the quaternion.
   setXYZW(Quaternion)   - 
Method in class org.proteinshader.math. Quaternion 
 Sets the xyzw-values of the quaternion to the xyzw-values of the
    quaternion given as an argument.
   setXYZW(Vec3d, Vec3d, Vec3d)   - 
Method in class org.proteinshader.math. Quaternion 
 Calculates and sets (x, y, z, w) of this quaternion based on
    the rotation contained in the 3 x 3 matrix [N B T].
   setXYZW(Vec3d, double)   - 
Method in class org.proteinshader.math. Quaternion 
 Calculates and sets (x, y, z, w) of this quaternion based on
    the axis and angle (in radians) given as aguments.
   SFWriterVisitor   - Class in  org.proteinshader.structure.visitor  A simple file writer subclass of Visitor that writes to a file all of
the data from each Model in a Structure.   SFWriterVisitor()   - 
Constructor for class org.proteinshader.structure.visitor. SFWriterVisitor 
 Constructs a SFWriterVisitor.
   SFWriterVisitor(String)   - 
Constructor for class org.proteinshader.structure.visitor. SFWriterVisitor 
 Constructs a SFWriterVisitor that will write to the output file
    given as an argument.
   SHADER_DIRECTORY   - 
Static variable in class org.proteinshader.graphics.shader. ShaderProgramFactory 
 Specifies the directory the OpenGL Shading Language
        vertex and fragment shaders are kept in.
   ShaderEnum   - Enum in  org.proteinshader.graphics.enums  Specifies the types of vertex and fragment shader pairs used in
the ProteinShader program, and provides a place to specify the
names of the shader files.   ShaderException   - Exception in  org.proteinshader.graphics.exceptions  To report errors that occur while creating or using an OpenGL shader.   ShaderException()   - 
Constructor for exception org.proteinshader.graphics.exceptions. ShaderException 
 Sets the default message to "An error occurred while using an
    OpenGL shader".
   ShaderException(String)   - 
Constructor for exception org.proteinshader.graphics.exceptions. ShaderException 
 The message given this constructor can be retrieved using
    getMessage().
   ShaderManager   - Class in  org.proteinshader.graphics.shader  Knows how to use a ShaderProgramFactory to compile OpenGL shader
programs and has methods to enable shaders.   ShaderManager()   - 
Constructor for class org.proteinshader.graphics.shader. ShaderManager 
 Constructs a ShaderManager.
   ShaderProgram   - Class in  org.proteinshader.graphics.shader  Stores information on an OpenGL Shading Language "program" object.   ShaderProgram(File, File, ShaderEnum)   - 
Constructor for class org.proteinshader.graphics.shader. ShaderProgram 
 Constructs a ShaderProgram object to store info on an OpenGL
    Shading Language "program" object.
   ShaderProgram()   - 
Constructor for class org.proteinshader.graphics.shader. ShaderProgram 
 Constructs a ShaderProgram object to store info on an OpenGL
    Shading Language "program" object.
   ShaderProgramFactory   - Class in  org.proteinshader.graphics.shader  Knows how to compile and link an OpenGL Shading Language vertex shader
and fragment shader to get an OpenGL "program" object.   ShaderProgramFactory()   - 
Constructor for class org.proteinshader.graphics.shader. ShaderProgramFactory 
 Constructs a ShaderProgramFactory.
   Shape   - Class in  org.proteinshader.graphics  Contains common methods needed by classes that draw geometric objects.   Shape()   - 
Constructor for class org.proteinshader.graphics. Shape 
 Constructor for use by subclasses.
   showAminoAcids(boolean)   - 
Method in interface org.proteinshader.gui. Mediator 
 Controls whether the Renderer will display AminoAcids.
   showAminoAcids(boolean)   - 
Method in class org.proteinshader.gui. MediatorImpl 
 Controls whether the Renderer will display AminoAcids.
   showAminoAcids(boolean)   - 
Method in class org.proteinshader.gui. Renderer 
 Controls whether the Renderer will display AminoAcids.
   showHeterogens(boolean)   - 
Method in interface org.proteinshader.gui. Mediator 
 Controls whether the Renderer will display Heterogens.
   showHeterogens(boolean)   - 
Method in class org.proteinshader.gui. MediatorImpl 
 Controls whether the Renderer will display Heterogens.
   showHeterogens(boolean)   - 
Method in class org.proteinshader.gui. Renderer 
 Controls whether the Renderer will display Heterogens.
   showSaveDialog(Component)   - 
Method in class org.proteinshader.gui.utils. ImageFileChooser 
 Sets the default filename for saving a file and then calls on the
    showSaveDialog(c) of the super class, JFileChooser, to pop open a 
    "Save File" file chooser dialog.
   showWaters(boolean)   - 
Method in interface org.proteinshader.gui. Mediator 
 Controls whether the Renderer will display Waters.
   showWaters(boolean)   - 
Method in class org.proteinshader.gui. MediatorImpl 
 Controls whether the Renderer will display Waters.
   showWaters(boolean)   - 
Method in class org.proteinshader.gui. Renderer 
 Controls whether the Renderer will display Waters.
   SideChainEnum   - Enum in  org.proteinshader.structure.enums  Provides an enumeration to specify how an amino
acid side chain should be displayed is it is shown
in combination with a tube or ribbon segment.   Silhouette   - Class in  org.proteinshader.graphics  Uses jitter to repeatedly render a translucent black silhouette of a Drawable
object for the purpose of antialiasing (smoothing jagged edges).   Silhouette(ShaderManager)   - 
Constructor for class org.proteinshader.graphics. Silhouette 
 Constructs a Silhouette object.
   SIN_HALF_FOVY   - 
Static variable in class org.proteinshader.gui.viewing. Camera 
 The sine of HALF_FOVY_RADIANS is used in pixel-angstrom conversions.
   slerp(Quaternion, double)   - 
Method in class org.proteinshader.math. Quaternion 
 Uses SLERP (Spherical Linear intERPolation) to calculate a
    Quaternion (a rotation) at any point t between the calling
    Quaternion and the argument Quaternion.
   SlerpDemo   - Class in  org.proteinshader.math  Tests the SLERP algorithm.   SlerpDemo()   - 
Constructor for class org.proteinshader.math. SlerpDemo 
 Creates a SlerpDemo object vectors and quaternions that can be
    used for testing.
   SLICES   - 
Static variable in class org.proteinshader.graphics. FrenetFrames 
 The number of slices for a cylinder is 10.
   SLIDER_INIT   - 
Static variable in class org.proteinshader.gui.components.controlpanel. VisibilityPanel 
 The initial value for the slider is 75 %.
   sortBetaStrands()   - 
Method in class org.proteinshader.structure. Chain 
 After all BetaStrand objects have been added to the Chain, this method can
    be called to sort the linked hash map (the linked list part does have order)
    according to the starting residue for each beta-strand.
   sortBetaStrands()   - 
Method in class org.proteinshader.structure. Model 
 Iterates through all Chains of the Model and modifies the linked hash map
    of beta-strands so that when an iterator for beta-strands is handed over
    (based on the linked list part) the beta-strands will be ordered according
    to the starting amino acid.
   sortBetaStrands()   - 
Method in class org.proteinshader.structure. Structure 
 Iterates through all Models of the Structure and modifies the linked hash
    map of beta-strands for each Chain so that when an iterator for beta-strands
    is handed over (based on the linked list part) the beta-strands will be 
    ordered according to the starting amino acid.
   SOURCEFORGE_WEB_SITE   - 
Static variable in class org.proteinshader.gui.components.menubar. HelpMenu 
  
   SPECULAR_COLOR   - 
Static variable in class org.proteinshader.graphics. Shape 
 The material specular color is (0.95, 0.95, 0.95, 1.0).
   SPECULAR_EXP   - 
Static variable in class org.proteinshader.graphics. FrenetFrames 
 The value for the specular exponent is 20.0.
   SPECULAR_LIGHT   - 
Static variable in class org.proteinshader.gui.viewing. Lighting 
 The specular light is { 0.95f, 0.95f, 0.95f, 0.0f }.
   Sphere   - Class in  org.proteinshader.graphics  Knows how to draw a sphere with texture coordinates, a surface normal,
and a tangent vector for each vertex.   Sphere()   - 
Constructor for class org.proteinshader.graphics. Sphere 
 Constructs a Sphere.
   SphereListInfo   - Class in  org.proteinshader.graphics.displaylists  Stores information on an OpenGL display list for a sphere.   SphereListInfo()   - 
Constructor for class org.proteinshader.graphics.displaylists. SphereListInfo 
 Constructs a SphereListInfo.
   SphereListInfo(int, StyleEnum, double, int, int)   - 
Constructor for class org.proteinshader.graphics.displaylists. SphereListInfo 
 Constructs a SphereListInfo.
   SphereReferences   - Class in  org.proteinshader.graphics.displaylists  Stores information on multiple OpenGL display lists that can be used
for rendering spheres with different degrees of detail.   SphereReferences()   - 
Constructor for class org.proteinshader.graphics.displaylists. SphereReferences 
 Constructs a SphereReferences object.
   SPINNER_MAX   - 
Static variable in class org.proteinshader.gui.components.controlpanel. OptimizePanel 
 The maximum value in a spinner is 100.
   SPINNER_MIN   - 
Static variable in class org.proteinshader.gui.components.controlpanel. OptimizePanel 
 The minimum value in a spinner is
        3 (same as Sphere.MIN_TILING).
   SpringLoadedJFrame   - Class in  org.proteinshader.gui.components  Provides a spring-loaded (retractable) JFrame that will adjust its
size and position relative to a parent JFrame.   SpringLoadedJFrame(JFrame, OrientationEnum)   - 
Constructor for class org.proteinshader.gui.components. SpringLoadedJFrame 
 Constructs a SpringLoadedJFrame using default width and height
    factors based on whether the spring-loaded frame is attached to
    its parent frame at the TOP, BOTTOM, LEFT, or RIGHT (see comments
    for DEFAULT_SHORT_FACTOR and DEFAULT_LONG_FACTOR).
   SpringLoadedJFrame(JFrame, OrientationEnum, double, double)   - 
Constructor for class org.proteinshader.gui.components. SpringLoadedJFrame 
 Constructs a SpringLoadedJFrame.
   STACKS   - 
Static variable in class org.proteinshader.graphics. FrenetFrames 
 The number of stacks for a cylinder is 1.
   START_CAP_COLOR   - 
Static variable in class org.proteinshader.graphics. ExtrudedShape 
 Segment start (amino end) caps are colored light blue
        because that is the CPK color for nitrogen.
   START_INDEX   - 
Static variable in class org.proteinshader.graphics.typography. GLF2Header 
 The start char integer starts at index 12 of the header.
   startAnimation()   - 
Method in class org.proteinshader.gui.components.controlpanel. MotionPanel 
 Start a rotation animation using the current slider values for the
    X-Axis speed and Y-Axis speed.
   startAnimation(double, double)   - 
Method in interface org.proteinshader.gui. Mediator 
 Starts an animation that rotates the image about its x-axis and/or
    y-axis based on the speeds given as argument.
   startAnimation(double, double)   - 
Method in class org.proteinshader.gui. MediatorImpl 
 Starts an animation that rotates the image about its x-axis and/or
    y-axis based on the speeds given as argument.
   startAnimation(double, double, GLCanvas)   - 
Method in class org.proteinshader.gui. Renderer 
 Uses an Animator object to rotate the protein image about its x-axis 
    and/or y-axis.
   startAnimation(double, double, GLCanvas)   - 
Method in class org.proteinshader.gui.viewing. Rotation 
 Uses an Animator object to rotate the protein image about its
    x-axis and/or y-axis.
   stopAnimation()   - 
Method in class org.proteinshader.gui.components.controlpanel. MotionPanel 
 Stops the rotation the was started with the startAnimation()
    button.
   stopAnimation()   - 
Method in interface org.proteinshader.gui. Mediator 
 Stops the rotation that was started with the startAnimation()
    method.
   stopAnimation()   - 
Method in class org.proteinshader.gui. MediatorImpl 
 Stops the rotation that was started with the startAnimation()
    method.
   stopAnimation()   - 
Method in class org.proteinshader.gui. Renderer 
 Stops the rotation animation that is started with startAnimation().
   stopAnimation()   - 
Method in class org.proteinshader.gui.viewing. Rotation 
 Stops the rotation animation that is started with
    startAnimation().
   Structure   - Class in  org.proteinshader.structure  Structure is the top-level container for objects that store
information on a protein structure from the
 Protein Data Bank .   Structure(String)   - 
Constructor for class org.proteinshader.structure. Structure 
 Constructs a Structure with the requested structureID.
   StructureReader   - Interface in  org.proteinshader.structure.io  StructureReader provides an interface for reading a protein structure
from a file.   StructureReaderException   - Exception in  org.proteinshader.structure.io.exceptions  Used to report that an error occurred while reading a file to create a
Structure.   StructureReaderException()   - 
Constructor for exception org.proteinshader.structure.io.exceptions. StructureReaderException 
 Sets the default message to "An error occurred while reading the
    file".
   StructureReaderException(String)   - 
Constructor for exception org.proteinshader.structure.io.exceptions. StructureReaderException 
 The message given this constructor can be retrieved using
    getMessage().
   StructureToGraphics   - Class in  org.proteinshader.graphics.adapter  Knows how to get numbers (such as zyz-coordinates and colors) from a
Drawable object (Atom, Bond, or Segment) and plug them into the right
graphics class for  rendering a Shape (Sphere, Cylinder, or Segment).   StructureToGraphics()   - 
Constructor for class org.proteinshader.graphics.adapter. StructureToGraphics 
 Constructs a StructureToGraphics object.
   StyleEnum   - Enum in  org.proteinshader.gui.enums  Provides an enumeration to specify what style should be used to display a
Structure: Cartoon (Tubes and Ribbons) or Atom (Spheres and Cylinders).   StyleMenu   - Class in  org.proteinshader.gui.components.menubar  Provides a menu for selecting the style to use when displaying a structure: 
Cartoon (Tubes or Ribbons) or Atom (Space Filling, Balls-andSticks, or Sticks).   StyleMenu(Mediator)   - 
Constructor for class org.proteinshader.gui.components.menubar. StyleMenu 
 Constructs a StyleMenu.
   StyleMenuListenerFactory   - Class in  org.proteinshader.gui.listeners.menubar  Creates all event listeners needed by the StyleMenu of the MainMenuBar.   StyleMenuListenerFactory(Mediator)   - 
Constructor for class org.proteinshader.gui.listeners.menubar. StyleMenuListenerFactory 
 Constructs a StyleMenuListenerFactory.
 
 
    
 T  
 
   TABLA_RASA_HEIGHT   - 
Static variable in class org.proteinshader.graphics.typography. TextLabelManager 
 The blank texture map is 128 pixels high.
   TABLA_RASA_WIDTH   - 
Static variable in class org.proteinshader.graphics.typography. TextLabelManager 
 The blank texture map is 128 pixels wide.
   takeScreenShot(ImageFormatEnum, File)   - 
Method in interface org.proteinshader.gui. Mediator 
 Causes the Render to take a screen shot of the current canvas image and save
    it as a JPEG, PNG or GIF file.
   takeScreenShot(ImageFormatEnum, File)   - 
Method in class org.proteinshader.gui. MediatorImpl 
 Causes the Render to take a screen shot of the current canvas image and save
    it as a JPEG, PNG or GIF file.
   takeScreenShot(ImageFormatEnum, File)   - 
Method in class org.proteinshader.gui. Renderer 
 Sets up a screen shot so that the next call to display() will cause the
    current image on the canvas to be saved to a JPEG, PNG or GIF file.
   TAN_HALF_FOVY   - 
Static variable in class org.proteinshader.gui.viewing. Camera 
 The tangent of HALF_FOVY_RADIANS is used in pixel-angstrom 
        conversions.
   test2ArgConstructor()   - 
Method in class org.proteinshader.math. LocalFrameDemo 
 Uses a rotation quaternion and a translation vector to create a
    LocalFrame and then prints the LocalFrame by using its toString()
    method.
   test6ArgConstructor()   - 
Method in class org.proteinshader.math. LocalFrameDemo 
 Uses a matrix of 3 column vectors, [N B T], and 3 doubles
    (x, y, z) to create a LocalFrame and then prints the LocalFrame.
   test7ArgConstructor()   - 
Method in class org.proteinshader.math. LocalFrameDemo 
 Uses xyzw-values for a quaternion and xyz-values for a translation
    vector to create a LocalFrame and then prints the LocalFrame.
   testMatrixToQuaternionConversion()   - 
Method in class org.proteinshader.math. QuaternionDemo 
 TEST A: Creates two matrices with known quaternion solutions and
    prints the matrices, the known solutions, and the solutions
    generated by class Quaternion.
   testMultiplyPointByQuaternion()   - 
Method in class org.proteinshader.math. QuaternionDemo 
 TEST C: Prints the results of several Point3d x Quaternion
    multiplications.
   testMultiplyPointMethod()   - 
Method in class org.proteinshader.math. LocalFrameDemo 
 Tests the multiply(Point3d) method of LocalFrame.
   testQuaternionToMatrixConversion()   - 
Method in class org.proteinshader.math. QuaternionDemo 
 TEST B: Prints the result of converting a Matrix to a Quaternion
    and then converting the Quaternion back to a Matrix.
   testSetRotationMethods()   - 
Method in class org.proteinshader.math. LocalFrameDemo 
 Creates a LocalFrame with the zero-arg constructor and then tests
    the setRotation() methods.
   testSetTranslationMethods()   - 
Method in class org.proteinshader.math. LocalFrameDemo 
 Creates a LocalFrame with the zero-arg constructor and then tests
    the setTranslation() methods.
   testSlerp()   - 
Method in class org.proteinshader.math. QuaternionDemo 
 TEST D: Tests the SLERP (Spherical Linear intERPolation) algorithm
    of class Quaternion.
   testVisitor(Structure)   - 
Method in class org.proteinshader.structure.visitor.demos. VisitorDemo 
 Creates a Visitor and tests it by activating debug mode so that it
    will print the ID of each object in the Structure.
   testZeroArgConstructor()   - 
Method in class org.proteinshader.math. LocalFrameDemo 
 Uses the zero-argument constructor to create and then print a
    LocalFrame.
   TextLabel   - Class in  org.proteinshader.graphics.typography  Stores a byte buffer with the bitmap for a text label that can be
applied to the curved surface of a 3D tube or ribbon.   TextLabel(String, int, int, int, int, int, ByteBuffer)   - 
Constructor for class org.proteinshader.graphics.typography. TextLabel 
 Constructs a TextLabel.
   TextLabelFactory   - Class in  org.proteinshader.graphics.typography  Uses a GlyphSet to create short text labels to place on segments of
a tube or ribbon.   TextLabelFactory()   - 
Constructor for class org.proteinshader.graphics.typography. TextLabelFactory 
 Constructs a TextLabelFactory.
   TextLabelManager   - Class in  org.proteinshader.graphics.typography  Knows how to create and cache OpenGL display lists holding the
glTexSubImage() with a bitmap for a text label that can be pasted
onto curved surfaces.   TextLabelManager()   - 
Constructor for class org.proteinshader.graphics.typography. TextLabelManager 
 Constructs a TextLabelManager.
   Texture   - Class in  org.proteinshader.graphics.textures  Stores information on an OpenGL texture object.   Texture(int, String, String, File)   - 
Constructor for class org.proteinshader.graphics.textures. Texture 
 Constructs a Texture object.
   TextureException   - Exception in  org.proteinshader.graphics.exceptions  To report errors that occur while loading a texture.   TextureException()   - 
Constructor for exception org.proteinshader.graphics.exceptions. TextureException 
 Sets the default message to "An error occurred while using a
    texture map".
   TextureException(String)   - 
Constructor for exception org.proteinshader.graphics.exceptions. TextureException 
 The message given this constructor can be retrieved using
    getMessage().
   TextureFactory   - Class in  org.proteinshader.graphics.textures  Reads texture files and creates OpenGL texture objects that can be
used for real-time halftoning.   TextureFactory()   - 
Constructor for class org.proteinshader.graphics.textures. TextureFactory 
 Constructs a TextureFactory.
   TextureManager   - Class in  org.proteinshader.graphics.textures  Uses a TextureFactory object to read textures from files and then
stores Java Texture objects that have  references to OpenGL texture
objects and menu names that can be used for selecting the textures.   TextureManager()   - 
Constructor for class org.proteinshader.graphics.textures. TextureManager 
 Constructs a TextureManager.
   THIN_SIDES_COLOR   - 
Static variable in class org.proteinshader.graphics. Ribbon 
 The default color for the narrow sides of the
        three-dimensional ribbon is gray.
   TIP_FRACTION   - 
Static variable in class org.proteinshader.graphics. FrenetFrames 
 The length of an arrow tip as a fraction of the axis length
        is 0.1.
   TIP_RADIUS   - 
Static variable in class org.proteinshader.graphics. FrenetFrames 
 The radius of the large end of an
        arrow tip is 0.08 angstroms.
   toggleBlendObjectEdges()   - 
Method in class org.proteinshader.graphics.adapter. StructureToGraphics 
 Toggles the boolean value that is set with the blendObjectsEdges() method.
   toggleBlendObjectEdges()   - 
Method in class org.proteinshader.gui. Renderer 
 Toggles the boolean value that is set with the blendObjectsEdges() method.
   togglePrintAngstroms()   - 
Method in class org.proteinshader.gui. Renderer 
 Toggles the memory of whether object to camera distances in angstroms (and
    tiling numbers) should be printed.
   ToolsMenu   - Class in  org.proteinshader.gui.components.menubar  Provides the Tools menu for the MainMenuBar, which can be used to open
and close the control panel on the right side of the GUI.   ToolsMenu(Mediator)   - 
Constructor for class org.proteinshader.gui.components.menubar. ToolsMenu 
 Constructs a ToolsMenu.
   ToolsMenuListenerFactory   - Class in  org.proteinshader.gui.listeners.menubar  Creates all event listeners needed by the ToolsMenu of the
MainMenuBar.   ToolsMenuListenerFactory(Mediator)   - 
Constructor for class org.proteinshader.gui.listeners.menubar. ToolsMenuListenerFactory 
 Constructs a ToolsMenuListenerFactory.
   TOP_MARGIN   - 
Static variable in class org.proteinshader.gui. ProteinShaderGUI 
 The top margin is the minimum number of
        pixels to leave between the top of the GUI
        window and the top of the monitor.
   toString()   - 
Method in class org.proteinshader.graphics.textures. NamePair 
 Returns the menu name and filename pair.
   toString()   - 
Method in class org.proteinshader.graphics.textures. Texture 
 Returns the menu name of the Texture.
   toString()   - 
Method in class org.proteinshader.gui.components.controlpanel. AntialiasingPanel 
 Returns a name suitable for use in a menu.
   toString()   - 
Method in class org.proteinshader.gui.components.controlpanel. AtomColorPanel 
 Returns a name suitable for use in a menu.
   toString()   - 
Method in class org.proteinshader.gui.components.controlpanel. AtomScalePanel 
 Returns a name suitable for use in a menu.
   toString()   - 
Method in class org.proteinshader.gui.components.controlpanel. AtomVisibilityPanel 
 Returns a name suitable for use in a menu.
   toString()   - 
Method in class org.proteinshader.gui.components.controlpanel. CartoonColorPanel 
 Returns a name suitable for use in a menu.
   toString()   - 
Method in class org.proteinshader.gui.components.controlpanel. CartoonSideChainsPanel 
 Returns a name suitable for use in a menu.
   toString()   - 
Method in class org.proteinshader.gui.components.controlpanel. CartoonVisibilityPanel 
 Returns a name suitable for use in a menu.
   toString()   - 
Method in class org.proteinshader.gui.components.controlpanel. DecorationsPanel 
 Returns a name suitable for use in a menu.
   toString()   - 
Method in class org.proteinshader.gui.components.controlpanel. MotionPanel 
 Returns a name suitable for use in a menu.
   toString()   - 
Method in class org.proteinshader.gui.components.controlpanel. OptimizePanel 
 Returns a name suitable for use in a menu.
   toString()   - 
Method in enum org.proteinshader.gui.enums. ImageFormatEnum 
 Returns the name of the ImageFormatEnum in a form suitable for use in
    a menu.
   toString()   - 
Method in enum org.proteinshader.gui.enums. JitterEnum 
 Returns the number of xy-coordinate pairs as a String so that the enum
    can be used in a Swing GUI menu.
   toString()   - 
Method in enum org.proteinshader.gui.enums. OrientationEnum 
 Returns the name of the OrientationEnum in a form suitable for use in
    a menu.
   toString()   - 
Method in enum org.proteinshader.gui.enums. StyleEnum 
 Returns the name of the StyleEnum in a form suitable for use in
    a menu.
   toString()   - 
Method in class org.proteinshader.math. LocalFrame 
 As a convenience for debugging, the toString() method returns
    the xyzw-components of the quaternion and the translation vector
    in the form "{ (x, y, z, w), (x, y, z) }".
   toString()   - 
Method in class org.proteinshader.math. Point3d 
 As a convenience for debugging, the toString() method returns
    the xyz-coordinates of the point in the form "(x, y, z)".
   toString()   - 
Method in class org.proteinshader.math. Quaternion 
 As a convenience for debugging, the toString() method returns
    the xyzw-components of the quaternion in the form "(x, y, z, w)".
   toString()   - 
Method in class org.proteinshader.math. Vec3d 
 As a convenience for debugging, the toString() method returns
    the xyz-coordinates of the vector in the form "(x, y, z)".
   toString()   - 
Method in class org.proteinshader.structure. Atom 
 Returns the ID of this Atom.
   toString()   - 
Method in class org.proteinshader.structure. BetaStrand 
 Returns the BetaStrand ID.
   toString()   - 
Method in class org.proteinshader.structure. Bond 
 Returns a String with the IDs of the source and destination atoms.
   toString()   - 
Method in class org.proteinshader.structure. Chain 
 Returns the chainID, which is usually a single letter.
   toString()   - 
Method in enum org.proteinshader.structure.enums. RegionEnum 
 Returns the menu name for the Region.
   toString()   - 
Method in enum org.proteinshader.structure.enums. ResidueEnum 
 Returns the Residue type as a String suitable for use in a menu:
    for example, "Amino Acids" rather then "AMINO_ACIDS".
   toString()   - 
Method in enum org.proteinshader.structure.enums. SideChainEnum 
 Returns the name of the SideChainEnum in a form suitable for use
    in a menu.
   toString()   - 
Method in class org.proteinshader.structure. Helix 
 Returns the Helix ID.
   toString()   - 
Method in class org.proteinshader.structure. Loop 
 Returns the Loop ID.
   toString()   - 
Method in class org.proteinshader.structure. Model 
 Returns the modelID, which is the Model's number.
   toString()   - 
Method in class org.proteinshader.structure. Residue 
 Returns the ID of this Residue.
   toString()   - 
Method in class org.proteinshader.structure. Segment 
 Returns the ID of this Segment.
   toString()   - 
Method in class org.proteinshader.structure. Structure 
 Returns the structureID, which is the sames as the PDB unique ID
    code.
   toString()   - 
Method in enum org.proteinshader.structure.visitor.enums. AAPortionMode 
 Returns the name of the AAPortionMode in a form suitable for use
    in a menu.
   translate(Point3d)   - 
Method in class org.proteinshader.math. LocalFrame 
 The vertex given as an argument is modified by adding to it the
    xyz-coordinates of the translation vector held by this LocalFrame.
   Tube   - Class in  org.proteinshader.graphics  Draws a segment of a three-dimensional tube by using a waist polygon
that approximates a circle as the number of vertices in the waist
polygon becomes large.   Tube()   - 
Constructor for class org.proteinshader.graphics. Tube 
 Constructs a Tube object that can be used for rendering tubes with
    a circular-shaped waist polygon.
   Tube(RegionEnum)   - 
Constructor for class org.proteinshader.graphics. Tube 
 Constructs a Tube object that can be used for rendering tubes with
    a circular-shaped waist polygon.
   Tube(double, int, float, float, boolean)   - 
Constructor for class org.proteinshader.graphics. Tube 
 Constructs a Tube object that can be used for rendering
    tubes with a circular-shaped waist polygon.
   TubeVertices   - Class in  org.proteinshader.structure.io  Creates the vertices for a segment of a three-dimensional tube by
using a waist polygon that approximates a circle as the number of
vertices in the waist polygon becomes large.   TubeVertices()   - 
Constructor for class org.proteinshader.structure.io. TubeVertices 
 Constructs a Tube object that can be used for rendering tubes with
    a circular-shaped waist polygon.
   TubeVertices(RegionEnum)   - 
Constructor for class org.proteinshader.structure.io. TubeVertices 
 Constructs a TubeVertices object that can be used for rendering
    tubes with a circular-shaped waist polygon.
   TubeVertices(double, int)   - 
Constructor for class org.proteinshader.structure.io. TubeVertices 
 Constructs a TubeVertices object that can be used for rendering
    tubes with a circular-shaped waist polygon.
   TWO_PI   - 
Static variable in class org.proteinshader.graphics. Shape 
 2*Pi is defined as 6.28318530717958.
   TWO_PI   - 
Static variable in class org.proteinshader.gui.viewing. Rotation 
 2*Pi is defined as 6.28318530717958,
        and is equal to 360 degrees.
   TWO_PI   - 
Static variable in class org.proteinshader.structure.io. TubeVertices 
 2*Pi is defined as 6.28318530717958.
 
 
    
 U  
 
   updateAtomScalePanel(StyleEnum)   - 
Method in class org.proteinshader.gui.components.controlpanel. ControlPanel 
 Tells the ModifierPanel to update the AtomScalePanel with the
    current style.
   updateAtomScalePanel(StyleEnum)   - 
Method in class org.proteinshader.gui.components.controlpanel. ModifierPanel 
 Updates the AtomScalePanel so that it knows the current style.
   updateCylinderCapsLabel(int)   - 
Method in class org.proteinshader.gui.components.controlpanel. OptimizePanel 
 Updates the tiling number text label below the 'Cylinder Caps'
    combo box menu, updates the spinner value to match, and also sets
    the 'Cylinder Caps' radio button as being selected.
   updateCylindersLabel(int)   - 
Method in class org.proteinshader.gui.components.controlpanel. OptimizePanel 
 Updates the tiling number text label below the 'Cylinders' combo
    box menu, updates the spinner value to match, and also sets the
    'Cylinders' radio button as being selected.
   updateFPSDisplay(double)   - 
Method in interface org.proteinshader.gui. Mediator 
 During a rotation animation the Renderer will call this method to
    update whatever GUI components display the current frames per
    second the animation is operating at.
   updateFPSDisplay(double)   - 
Method in class org.proteinshader.gui. MediatorImpl 
 During a rotation animation the Renderer will call this method to
    update the ControlPanel with the current frames per second the
    animation is operating at.
   updateFramesPerSecondDisplay(double)   - 
Method in class org.proteinshader.gui.components.controlpanel. ControlPanel 
 Tells the ModifierPanel to update the current frames per second
    that a rotation animation is running at.
   updateFramesPerSecondDisplay(double)   - 
Method in class org.proteinshader.gui.components.controlpanel. ModifierPanel 
 Tells the MotionPanel to update the current frames per second that
    a rotation animation is running at.
   updateFramesPerSecondDisplay(double)   - 
Method in class org.proteinshader.gui.components.controlpanel. MotionPanel 
 Updates the text field for the current frames per second that a
    rotation animation is running at.
   updateListsOfVisibles(boolean, boolean, boolean, boolean, boolean, boolean)   - 
Method in class org.proteinshader.structure. Model 
 Finds visible Drawable objects and transfers them to a list of opaque
    Segments, opaque Atoms and Bonds, or a list of translucent Drawables.
   updateModelInfo()   - 
Method in class org.proteinshader.gui.components.controlpanel. ControlPanel 
 This method should be called whenever a new Model has been
    selected, so that the ModifierPanel can call on any of its
    subpanels that need to update themselves.
   updateModelInfo()   - 
Method in class org.proteinshader.gui.components.controlpanel. ModifierPanel 
 Tells the RadioPanel to update the 'Loop:', 'Helices:' and
    'Strands:' menu choices.
   updateModelInfo()   - 
Method in class org.proteinshader.gui.components.controlpanel. RadioPanel 
 Updates the "Helices:", "BetaStrands:", and "Loops:" menus with
    the Helices and BetaStrands of the current Model.
   updateModelViewMatrix(GL)   - 
Method in class org.proteinshader.gui.viewing. Camera 
 Updates a copy of the OpenGL model view matrix.
   updateRenderer()   - 
Method in interface org.proteinshader.gui. Mediator 
 Updates the arrays of visible objects (opaque and translucent
    Drawables) held by the Renderer and then has the canvas call display().
   updateRenderer()   - 
Method in class org.proteinshader.gui. MediatorImpl 
 Updates the arrays of visible objects (opaque and translucent
    Drawables) held by the Renderer and then has the canvas call display().
   updateSpheresLabel(int)   - 
Method in class org.proteinshader.gui.components.controlpanel. OptimizePanel 
 Updates the tiling number text label below the "Spheres" combo
    box menu, updates the spinner value to match, and also sets the
    "Spheres" radio button as being selected.
   updateStyle(StyleEnum)   - 
Method in class org.proteinshader.gui.components.controlpanel. AtomScalePanel 
 Changes will be applied only if the style is SPACE_FILLING or 
    BALLS_AND_STICKS.
   usageMessage   - 
Static variable in class org.proteinshader.structure.io.demos. PDBStructureReaderDemo 
  
   usageMessage   - 
Static variable in class org.proteinshader.structure.io.demos. SegmentDemo 
  
   usageMessage   - 
Static variable in class org.proteinshader.structure.visitor.demos. ModifierVisitorDemo 
  
   usageMessage   - 
Static variable in class org.proteinshader.structure.visitor.demos. VisitorDemo 
  
   useTextFieldsToUpdateSpeed()   - 
Method in class org.proteinshader.gui.components.controlpanel. MotionPanel 
 Gets the speed values from the text fields and uses them to update
    the sliders (without firing a change event for the sliders), and,
    if an animation is in progress, the new speeds will be forwarded
    to the Mediator.
   useXAxisSpinnerToUpdateSpeed()   - 
Method in class org.proteinshader.gui.components.controlpanel. MotionPanel 
 Uses the speed from the X-Axis slider to update the X-Axis text
    field, and, if an animation is in progress, the new speed will be
    sent to the Mediator.
   useYAxisSpinnerToUpdateSpeed()   - 
Method in class org.proteinshader.gui.components.controlpanel. MotionPanel 
 Uses the speed from the Y-Axis slider to update the Y-Axis text
    field, and, if an animation is in progress, the new speed will be
    sent to the Mediator.
 
 
    
 V  
 
   valueOf(String)   - 
Static method in enum org.proteinshader.graphics.enums. ShaderEnum 
 Returns the enum constant of this type with the specified name.
   valueOf(String)   - 
Static method in enum org.proteinshader.gui.components.controlpanel. RadioPanel.Type 
 Returns the enum constant of this type with the specified name.
   valueOf(String)   - 
Static method in enum org.proteinshader.gui.enums. ImageFormatEnum 
 Returns the enum constant of this type with the specified name.
   valueOf(String)   - 
Static method in enum org.proteinshader.gui.enums. JitterEnum 
 Returns the enum constant of this type with the specified name.
   valueOf(String)   - 
Static method in enum org.proteinshader.gui.enums. OrientationEnum 
 Returns the enum constant of this type with the specified name.
   valueOf(String)   - 
Static method in enum org.proteinshader.gui.enums. RadioButtonEnum 
 Returns the enum constant of this type with the specified name.
   valueOf(String)   - 
Static method in enum org.proteinshader.gui.enums. StyleEnum 
 Returns the enum constant of this type with the specified name.
   valueOf(String)   - 
Static method in enum org.proteinshader.gui.listeners. CanvasMouseListener.Mode 
 Returns the enum constant of this type with the specified name.
   valueOf(String)   - 
Static method in enum org.proteinshader.structure.enums. AAColorEnum 
 Returns the enum constant of this type with the specified name.
   valueOf(String)   - 
Static method in enum org.proteinshader.structure.enums. AminoAcidEnum 
 Returns the enum constant of this type with the specified name.
   valueOf(String)   - 
Static method in enum org.proteinshader.structure.enums. AtomEnum 
 Returns the enum constant of this type with the specified name.
   valueOf(String)   - 
Static method in enum org.proteinshader.structure.enums. BondEnum 
 Returns the enum constant of this type with the specified name.
   valueOf(String)   - 
Static method in enum org.proteinshader.structure.enums. CPKColorEnum 
 Returns the enum constant of this type with the specified name.
   valueOf(String)   - 
Static method in enum org.proteinshader.structure.enums. DecorationEnum 
 Returns the enum constant of this type with the specified name.
   valueOf(String)   - 
Static method in enum org.proteinshader.structure.enums. DrawableEnum 
 Returns the enum constant of this type with the specified name.
   valueOf(String)   - 
Static method in enum org.proteinshader.structure.enums. HelixEnum 
 Returns the enum constant of this type with the specified name.
   valueOf(int)   - 
Static method in enum org.proteinshader.structure.enums. HelixEnum 
 Returns the HelixEnum corresponding to the PDB classification
    number given as an argument.
   valueOf(String)   - 
Static method in enum org.proteinshader.structure.enums. HelixShapeEnum 
 Returns the enum constant of this type with the specified name.
   valueOf(String)   - 
Static method in enum org.proteinshader.structure.enums. RegionColorEnum 
 Returns the enum constant of this type with the specified name.
   valueOf(String)   - 
Static method in enum org.proteinshader.structure.enums. RegionEnum 
 Returns the enum constant of this type with the specified name.
   valueOf(String)   - 
Static method in enum org.proteinshader.structure.enums. ResidueEnum 
 Returns the enum constant of this type with the specified name.
   valueOf(String)   - 
Static method in enum org.proteinshader.structure.enums. SideChainEnum 
 Returns the enum constant of this type with the specified name.
   valueOf(String)   - 
Static method in enum org.proteinshader.structure.enums. VisibilityEnum 
 Returns the enum constant of this type with the specified name.
   valueOf(String)   - 
Static method in enum org.proteinshader.structure.io.enums. CategoryEnum 
 Returns the enum constant of this type with the specified name.
   valueOf(String)   - 
Static method in enum org.proteinshader.structure.io.enums. RecordEnum 
 Returns the enum constant of this type with the specified name.
   valueOf(String)   - 
Static method in enum org.proteinshader.structure.visitor.enums. AAPortionMode 
 Returns the enum constant of this type with the specified name.
   valueOf(String)   - 
Static method in enum org.proteinshader.structure.visitor.enums. RegionMode 
 Returns the enum constant of this type with the specified name.
   valueOf(String)   - 
Static method in enum org.proteinshader.structure.visitor.enums. ResidueMode 
 Returns the enum constant of this type with the specified name.
   valueOfMenuName(String)   - 
Static method in enum org.proteinshader.gui.enums. ImageFormatEnum 
 Returns the ImageFormatEnum with the same menu name as the String given 
    as an argument.
   valueOfMenuName(String)   - 
Static method in enum org.proteinshader.gui.enums. OrientationEnum 
 Returns the OrientationEnum with the same menu name as the String
    given as an argument.
   valueOfMenuName(String)   - 
Static method in enum org.proteinshader.gui.enums. StyleEnum 
 Returns the StyleEnum with the same menu name as the String
    given as an argument.
   valueOfMenuName(String)   - 
Static method in enum org.proteinshader.structure.enums. SideChainEnum 
 Returns the SideChainEnum with the same menu name as the String
    given as an argument.
   valueOfMenuName(String)   - 
Static method in enum org.proteinshader.structure.visitor.enums. AAPortionMode 
 Returns the AAPortionMode with the same menu name as the String
    given as an argument.
   values()   - 
Static method in enum org.proteinshader.graphics.enums. ShaderEnum 
 Returns an array containing the constants of this enum type, in
the order they're declared.
   values()   - 
Static method in enum org.proteinshader.gui.components.controlpanel. RadioPanel.Type 
 Returns an array containing the constants of this enum type, in
the order they're declared.
   values()   - 
Static method in enum org.proteinshader.gui.enums. ImageFormatEnum 
 Returns an array containing the constants of this enum type, in
the order they're declared.
   values()   - 
Static method in enum org.proteinshader.gui.enums. JitterEnum 
 Returns an array containing the constants of this enum type, in
the order they're declared.
   values()   - 
Static method in enum org.proteinshader.gui.enums. OrientationEnum 
 Returns an array containing the constants of this enum type, in
the order they're declared.
   values()   - 
Static method in enum org.proteinshader.gui.enums. RadioButtonEnum 
 Returns an array containing the constants of this enum type, in
the order they're declared.
   values()   - 
Static method in enum org.proteinshader.gui.enums. StyleEnum 
 Returns an array containing the constants of this enum type, in
the order they're declared.
   values()   - 
Static method in enum org.proteinshader.gui.listeners. CanvasMouseListener.Mode 
 Returns an array containing the constants of this enum type, in
the order they're declared.
   values()   - 
Static method in enum org.proteinshader.structure.enums. AAColorEnum 
 Returns an array containing the constants of this enum type, in
the order they're declared.
   values()   - 
Static method in enum org.proteinshader.structure.enums. AminoAcidEnum 
 Returns an array containing the constants of this enum type, in
the order they're declared.
   values()   - 
Static method in enum org.proteinshader.structure.enums. AtomEnum 
 Returns an array containing the constants of this enum type, in
the order they're declared.
   values()   - 
Static method in enum org.proteinshader.structure.enums. BondEnum 
 Returns an array containing the constants of this enum type, in
the order they're declared.
   values()   - 
Static method in enum org.proteinshader.structure.enums. CPKColorEnum 
 Returns an array containing the constants of this enum type, in
the order they're declared.
   values()   - 
Static method in enum org.proteinshader.structure.enums. DecorationEnum 
 Returns an array containing the constants of this enum type, in
the order they're declared.
   values()   - 
Static method in enum org.proteinshader.structure.enums. DrawableEnum 
 Returns an array containing the constants of this enum type, in
the order they're declared.
   values()   - 
Static method in enum org.proteinshader.structure.enums. HelixEnum 
 Returns an array containing the constants of this enum type, in
the order they're declared.
   values()   - 
Static method in enum org.proteinshader.structure.enums. HelixShapeEnum 
 Returns an array containing the constants of this enum type, in
the order they're declared.
   values()   - 
Static method in enum org.proteinshader.structure.enums. RegionColorEnum 
 Returns an array containing the constants of this enum type, in
the order they're declared.
   values()   - 
Static method in enum org.proteinshader.structure.enums. RegionEnum 
 Returns an array containing the constants of this enum type, in
the order they're declared.
   values()   - 
Static method in enum org.proteinshader.structure.enums. ResidueEnum 
 Returns an array containing the constants of this enum type, in
the order they're declared.
   values()   - 
Static method in enum org.proteinshader.structure.enums. SideChainEnum 
 Returns an array containing the constants of this enum type, in
the order they're declared.
   values()   - 
Static method in enum org.proteinshader.structure.enums. VisibilityEnum 
 Returns an array containing the constants of this enum type, in
the order they're declared.
   values()   - 
Static method in enum org.proteinshader.structure.io.enums. CategoryEnum 
 Returns an array containing the constants of this enum type, in
the order they're declared.
   values()   - 
Static method in enum org.proteinshader.structure.io.enums. RecordEnum 
 Returns an array containing the constants of this enum type, in
the order they're declared.
   values()   - 
Static method in enum org.proteinshader.structure.visitor.enums. AAPortionMode 
 Returns an array containing the constants of this enum type, in
the order they're declared.
   values()   - 
Static method in enum org.proteinshader.structure.visitor.enums. RegionMode 
 Returns an array containing the constants of this enum type, in
the order they're declared.
   values()   - 
Static method in enum org.proteinshader.structure.visitor.enums. ResidueMode 
 Returns an array containing the constants of this enum type, in
the order they're declared.
   Vec3d   - Class in  org.proteinshader.math  This class is used to create a vector with 3 elements of type double.   Vec3d()   - 
Constructor for class org.proteinshader.math. Vec3d 
 Constructs a vector of 3 elements {x, y, z} filled with zeros.
   Vec3d(double, double, double)   - 
Constructor for class org.proteinshader.math. Vec3d 
 Constructs a vector with three public attributes: x, y, and z.
   VectorAndPointDemo   - Class in  org.proteinshader.math  Performs some simple tests on the Vec3d and Point3d classes.   VectorAndPointDemo()   - 
Constructor for class org.proteinshader.math. VectorAndPointDemo 
  
   VisibilityEnum   - Enum in  org.proteinshader.structure.enums  Provides an enumeration to specify if a Drawable object is opaque,
translucent, or invisible.   VisibilityMenu   - Class in  org.proteinshader.gui.components.menubar  Provides the Vi menu for the MainMenuBar.   VisibilityMenu(Mediator)   - 
Constructor for class org.proteinshader.gui.components.menubar. VisibilityMenu 
 Constructs a VisibilityMenu.
   VisibilityMenuListenerFactory   - Class in  org.proteinshader.gui.listeners.menubar  Creates all event listeners needed by the VisibilityMenu of the MainMenuBar.   VisibilityMenuListenerFactory(Mediator)   - 
Constructor for class org.proteinshader.gui.listeners.menubar. VisibilityMenuListenerFactory 
 Constructs a VisibilityMenuListenerFactory.
   VisibilityPanel   - Class in  org.proteinshader.gui.components.controlpanel  This control panel allows the user to modify the visibility status and
alpha value of Drawable objects.   VisibilityPanel(Mediator, Frame, RadioPanel)   - 
Constructor for class org.proteinshader.gui.components.controlpanel. VisibilityPanel 
 Constructs a VisibilityPanel.
   VisibilityPanelListenerFactory   - Class in  org.proteinshader.gui.listeners.controlpanel  Creates the listener for the VisibilityPanel.   VisibilityPanelListenerFactory(Mediator, VisibilityPanel)   - 
Constructor for class org.proteinshader.gui.listeners.controlpanel. VisibilityPanelListenerFactory 
 Constructs a VisibilityPanelListenerFactory.
   VisibilityVisitor   - Class in  org.proteinshader.structure.visitor  Knows how to traverse the Structure object hierarchy and find all
visible Drawable objects.   VisibilityVisitor()   - 
Constructor for class org.proteinshader.structure.visitor. VisibilityVisitor 
 Constructs a VisibilityVisitor.
   visit(AminoAcid)   - 
Method in class org.proteinshader.structure.visitor. AminoAcidLabelVisitor 
 Adds a residueID to the list of labels if it has not been seen
    before.
   visit(Atom)   - 
Method in class org.proteinshader.structure.visitor. BondDestroyerVisitor 
 Removes all Bonds on the Atom.
   visit(Structure)   - 
Method in class org.proteinshader.structure.visitor. BondGeneratorVisitor 
 Generates all standard Bonds (AminoAcids and Waters) for the
    Structure.
   visit(Model)   - 
Method in class org.proteinshader.structure.visitor. BondGeneratorVisitor 
 Generates all standard Bonds (AminoAcids and Waters) for the
    Model.
   visit(Chain)   - 
Method in class org.proteinshader.structure.visitor. BondGeneratorVisitor 
 Generates all standard Bonds (AminoAcids and Waters) for the
    Chain.
   visit(AminoAcid)   - 
Method in class org.proteinshader.structure.visitor. BondGeneratorVisitor 
 Uses Atom distances to generate Bonds within the AminoAcid, and
    also uses the memory of the last carbonyl carbon seen to check for
    a possible peptide Bond.
   visit(Heterogen)   - 
Method in class org.proteinshader.structure.visitor. BondGeneratorVisitor 
 NO OPERATION: This method is not needed because non-water
    heterogen bonds are given explicitly in the PDB structure entry.
   visit(Water)   - 
Method in class org.proteinshader.structure.visitor. BondGeneratorVisitor 
 If the Water includes hydrogen atoms, then Bonds will be created
    between the oxygen and each hydrogen (after checking that the Bond
    lengths are reasonable).
   visit(Atom)   - 
Method in class org.proteinshader.structure.visitor. BondGeneratorVisitor 
 Checks if the Atom is a possible Bond destination Atom for any
    previously seen Atoms of the same Residue, and then adds the Atom
    to the list of Atoms to remember.
   visit(Bond)   - 
Method in class org.proteinshader.structure.visitor. BondGeneratorVisitor 
 NO OPERATION: This Visitor method is not needed by the bond
    generator.
   visit(Model)   - 
Method in class org.proteinshader.structure.visitor. BoundsVisitor 
 Finds the min and max values for x, y, and z of the Model and then
    calls setter methods on the Model so that these values are
    remembered.
   visit(Atom)   - 
Method in class org.proteinshader.structure.visitor. BoundsVisitor 
 Checks xyz-coordinates to look for any new min or new max.
   visit(Chain)   - 
Method in class org.proteinshader.structure.visitor. FrenetFrameGeneratorVisitor 
 All AminoAcids of the Chain will be visited.
   visit(AminoAcid)   - 
Method in class org.proteinshader.structure.visitor. FrenetFrameGeneratorVisitor 
 The AminoAcid being visited is considered as CA(i+1), while the
    last two AminoAcids are considered to be CA(i) and CA(i-1).
   visit(Model)   - 
Method in class org.proteinshader.structure.visitor. LoopGeneratorVisitor 
 Generates all Loop objects needed by the Model.
   visit(Chain)   - 
Method in class org.proteinshader.structure.visitor. LoopGeneratorVisitor 
 Generates Loop objects from AminoAcids that do not already
    belong to any Region.
   visit(AminoAcid)   - 
Method in class org.proteinshader.structure.visitor. LoopGeneratorVisitor 
 Checks whether a start AminoAcid and an end AminoAcid for a Loop
    have been found, and creates a new Loop as needed.
   visit(Segment)   - 
Method in class org.proteinshader.structure.visitor. ModifierVisitor 
 If a SegmentModifier has been added, it will be used to modify
    the Segment.
   visit(Atom)   - 
Method in class org.proteinshader.structure.visitor. ModifierVisitor 
 If an AtomModifier has been added, it will be used to modify
    the Atom.
   visit(Bond)   - 
Method in class org.proteinshader.structure.visitor. ModifierVisitor 
 If a BondModifier has been added, it will be used to modify the
    Bond.
   visit(Structure)   - 
Method in class org.proteinshader.structure.visitor. SFWriterVisitor 
 Writes a Structure's data to a simple text file.
   visit(Model)   - 
Method in class org.proteinshader.structure.visitor. SFWriterVisitor 
 Writes all information held by a Model.
   visit(Chain)   - 
Method in class org.proteinshader.structure.visitor. SFWriterVisitor 
 Writes all information held by a Chain.
   visit(Loop)   - 
Method in class org.proteinshader.structure.visitor. SFWriterVisitor 
 Writes all information held by a Loop.
   visit(Helix)   - 
Method in class org.proteinshader.structure.visitor. SFWriterVisitor 
 Writes all information held by a Helix.
   visit(BetaStrand)   - 
Method in class org.proteinshader.structure.visitor. SFWriterVisitor 
 Writes all information held by a BetaStrand.
   visit(Segment)   - 
Method in class org.proteinshader.structure.visitor. SFWriterVisitor 
 Writes information on a Segment.
   visit(AminoAcid)   - 
Method in class org.proteinshader.structure.visitor. SFWriterVisitor 
 Writes all information held by an AminoAcid.
   visit(Heterogen)   - 
Method in class org.proteinshader.structure.visitor. SFWriterVisitor 
 Writes all information held by a Heterogen.
   visit(Water)   - 
Method in class org.proteinshader.structure.visitor. SFWriterVisitor 
 Writes all information held by a Water.
   visit(Atom)   - 
Method in class org.proteinshader.structure.visitor. SFWriterVisitor 
 Writes all information held by an Atom.
   visit(Bond)   - 
Method in class org.proteinshader.structure.visitor. SFWriterVisitor 
 Writes all information held by a Bond.
   visit(Atom)   - 
Method in class org.proteinshader.structure.visitor. VisibilityVisitor 
 Tests the visiblity of the Atom in order to determine if it should
    be transferred to the opaque Atoms and Bonds list or the translucent
    Drawables list.
   visit(Bond)   - 
Method in class org.proteinshader.structure.visitor. VisibilityVisitor 
 Tests the visiblity of the Bond in order to determine if it should be
    transferred to the opaque Atoms and Bonds or translucent Drawables list.
   visit(Segment)   - 
Method in class org.proteinshader.structure.visitor. VisibilityVisitor 
 Tests the visiblity of the Segment in order to determine if it should be 
    transferred to the opaque Segments or translucent Drawables list.
   visit(Structure)   - 
Method in class org.proteinshader.structure.visitor. Visitor 
 Traverses all Models owned by the Structure.
   visit(Model)   - 
Method in class org.proteinshader.structure.visitor. Visitor 
 Traverses all Chains owned by the Model.
   visit(Chain)   - 
Method in class org.proteinshader.structure.visitor. Visitor 
 Uses the current ResidueMode to determine if it should traverse
    the AminoAcids, Heterogens, and/or Waters owned by the Chain.
   visit(Loop)   - 
Method in class org.proteinshader.structure.visitor. Visitor 
 If debug is true, the loopID will be printed to standard out.
   visit(Helix)   - 
Method in class org.proteinshader.structure.visitor. Visitor 
 If debug is true, the helixID, startResidueID, and endResidueID
    will be printed to standard out.
   visit(BetaStrand)   - 
Method in class org.proteinshader.structure.visitor. Visitor 
 If debug is true, the betaStrandID, startResidueID, and
    endResidueID will be printed to standard out.
   visit(AminoAcid)   - 
Method in class org.proteinshader.structure.visitor. Visitor 
 Traverses all Atoms owned by the AminoAcid if AAPortionMode is
    set to ENTIRE_AA.
   visit(Heterogen)   - 
Method in class org.proteinshader.structure.visitor. Visitor 
 Traverses all Atoms owned by the Heterogen.
   visit(Water)   - 
Method in class org.proteinshader.structure.visitor. Visitor 
 Traverses all Atoms owned by the Water.
   visit(Atom)   - 
Method in class org.proteinshader.structure.visitor. Visitor 
 Traverses all Bonds owned by the Atom.
   visit(Bond)   - 
Method in class org.proteinshader.structure.visitor. Visitor 
 If debug is true, the atomID of both the source Atom and the
    destination Atom will be printed to standard out.
   visit(Segment)   - 
Method in class org.proteinshader.structure.visitor. Visitor 
 If debug is true, the segmentID will be printed to standard out.
   Visitable   - Interface in  org.proteinshader.structure.visitor  This interface requires an accept( Visitor ) method, which should be
used to call back to the Visitor so that the Visitor can perform one
or more operations on the Visitable object.   Visitor   - Class in  org.proteinshader.structure.visitor  This self-propelled Visitor knows how to traverse the hierarchy of
data objects contained by a Structure.   Visitor()   - 
Constructor for class org.proteinshader.structure.visitor. Visitor 
 No-arg constuctor sets ResidueMode.ALL, RegionMode.NO_REGIONS,
    AAPortionMode to ENTIRE_AA, restrictions (AminoAcid type, Atom
    type, and Atom ID) to null, and debug to false.
   VisitorDemo   - Class in  org.proteinshader.structure.visitor.demos  For testing the traversal logic of the Visitor class.   VisitorDemo()   - 
Constructor for class org.proteinshader.structure.visitor.demos. VisitorDemo 
 Constructs a VisitorDemo.
   VisitorException   - Exception in  org.proteinshader.structure.visitor.exceptions  Used to report that an error occurred while visiting an object.   VisitorException()   - 
Constructor for exception org.proteinshader.structure.visitor.exceptions. VisitorException 
 Sets the default message to

    "ERROR: A problem occurred while visiting a Visitable object".
   VisitorException(String)   - 
Constructor for exception org.proteinshader.structure.visitor.exceptions. VisitorException 
 The message given this constructor can be retrieved using
    getMessage().
 
 
    
 W  
 
   w   - 
Variable in class org.proteinshader.math. Quaternion 
 The public w-component.
   Water   - Class in  org.proteinshader.structure  This concrete subclass of the abstract class Residue stores
information on a water molecule in a PDB structure entry.   Water(String, String, String, String)   - 
Constructor for class org.proteinshader.structure. Water 
 Constructs a Water with the requested residueID.
   WHEEL_SCALE_FACTOR   - 
Static variable in class org.proteinshader.gui.listeners. CanvasMouseListener 
 When the mouse wheel is used for zooming, this scaling factory
        will determine how many angstroms the camera moves in or out
        for each wheel click.
   WIDTH   - 
Static variable in class org.proteinshader.graphics.typography. TextLabel 
 The width is 128 pixels.
   WIDTH_INDEX   - 
Static variable in class org.proteinshader.graphics.typography. GLF2Header 
 The bitmap width integer starts at index 4 of the header.
   windowActivated(WindowEvent)   - 
Method in class org.proteinshader.gui.components. SpringLoadedJFrame 
 NOT USED: This method is called when the parent frame is set to
    be the active window.
   windowClosed(WindowEvent)   - 
Method in class org.proteinshader.gui.components. SpringLoadedJFrame 
 NOT USED: This method is called when the parent frame has been
    closed as the result of calling dispose on the window.
   windowClosing(WindowEvent)   - 
Method in class org.proteinshader.gui.components. SpringLoadedJFrame 
 NOT USED: This method is called when the user attempts to close
    the parent frame from its system menu.
   windowDeactivated(WindowEvent)   - 
Method in class org.proteinshader.gui.components. SpringLoadedJFrame 
 NOT USED: This method is called when the parent frame is no longer
    the active window.
   windowDeiconified(WindowEvent)   - 
Method in class org.proteinshader.gui.components. SpringLoadedJFrame 
 This method is called automatically when the parent frame is
    deiconified, and if this frame was hidden when the parent had been
    iconified, then this frame will be made visible again.
   windowIconified(WindowEvent)   - 
Method in class org.proteinshader.gui.components. SpringLoadedJFrame 
 This method is called automatically when the parent frame is
    iconified, and if this frame is visible, it will be hidden.
   WindowListenerFactory   - Class in  org.proteinshader.gui.listeners  Creates a window listener for closing the retractable control panel
that opens on the right-side of the GUI.   WindowListenerFactory(Mediator)   - 
Constructor for class org.proteinshader.gui.listeners. WindowListenerFactory 
 Constructs a WindowListenerFactory.
   windowOpened(WindowEvent)   - 
Method in class org.proteinshader.gui.components. SpringLoadedJFrame 
 NOT USED: This method is called when the parent frame is made
    visible for the first time.
   write(GL, GLAutoDrawable, double)   - 
Method in class org.proteinshader.gui.utils. FpsLabel 
 Updates the frames per second display on the canvas.
   writeDescription(Description)   - 
Method in class org.proteinshader.structure.visitor. SFWriterVisitor 
 writeDescription() - writes all information on a Description.
   writeFps(GL, GLAutoDrawable)   - 
Method in class org.proteinshader.gui.viewing. Rotation 
 Updates the frames per second display on the canvas.
   WriterVisitorException   - Exception in  org.proteinshader.structure.visitor.exceptions  Used to report that an error occurred while trying to write info on a
Visitable object.   WriterVisitorException()   - 
Constructor for exception org.proteinshader.structure.visitor.exceptions. WriterVisitorException 
 Sets the default message to

    "ERROR: A problem occurred while trying to write a file".
   WriterVisitorException(String)   - 
Constructor for exception org.proteinshader.structure.visitor.exceptions. WriterVisitorException 
 The message given this constructor can be retrieved using
    getMessage().
   writeStructure(Structure, String)   - 
Method in class org.proteinshader.structure.io.demos. PDBStructureReaderDemo 
 Writes a Structure to a file by using a SFWriterVisitor.
   writeStructure(Structure, String)   - 
Method in class org.proteinshader.structure.io.demos. SegmentDemo 
 Writes a Structure to a file by using a SFWriterVisitor.
   writeStructure(Structure, String)   - 
Method in class org.proteinshader.structure.visitor.demos. ModifierVisitorDemo 
 Writes a Structure to a file by using a SFWriterVisitor.
 
 
    
 X  
 
   X   - 
Static variable in class org.proteinshader.graphics. FrenetFrames 
 The x-axis is green.
   x   - 
Variable in class org.proteinshader.math. Point3d 
 The public x-coordinate of this three element point.
   x   - 
Variable in class org.proteinshader.math. Quaternion 
 The public x-component.
   x   - 
Variable in class org.proteinshader.math. Vec3d 
 The public x-coordinate of this three element vector.
   X1_INDEX   - 
Static variable in class org.proteinshader.graphics.typography. GLF2Header 
 The bitmap width integer starts at index 8 of the header.
   X2_INDEX   - 
Static variable in class org.proteinshader.graphics.typography. GLF2Header 
 The start char integer starts at index 16 of the header.
   X_AXIS   - 
Static variable in class org.proteinshader.gui.viewing. Rotation 
 Vector representing an x-axis.
   X_OFFSET   - 
Static variable in class org.proteinshader.graphics.typography. TextLabelManager 
 The x-offset for pasting a subimage is 47 pixels.
   X_SPEED_INIT   - 
Static variable in class org.proteinshader.gui.components.controlpanel. MotionPanel 
 The initial rotation speed for the
        X-Axis is 0 degrees per second.
 
 
    
 Y  
 
   Y   - 
Static variable in class org.proteinshader.graphics. FrenetFrames 
 The y-axis is yellow.
   y   - 
Variable in class org.proteinshader.math. Point3d 
 The public y-coordinate of this three element point.
   y   - 
Variable in class org.proteinshader.math. Quaternion 
 The public y-component.
   y   - 
Variable in class org.proteinshader.math. Vec3d 
 The public y-coordinate of this three element vector.
   Y1_INDEX   - 
Static variable in class org.proteinshader.graphics.typography. GLF2Header 
 The bitmap height integer starts at index 12 of the header.
   Y2_INDEX   - 
Static variable in class org.proteinshader.graphics.typography. GLF2Header 
 The end char integer starts at index 20 of the header.
   Y_AXIS   - 
Static variable in class org.proteinshader.gui.viewing. Rotation 
 Vector representing an y-axis.
   Y_OFFSET   - 
Static variable in class org.proteinshader.graphics.typography. TextLabelManager 
 The y-offset is 0 pasting a submimage is 0 pixels.
   Y_SPEED_INIT   - 
Static variable in class org.proteinshader.gui.components.controlpanel. MotionPanel 
 The initial rotation speed for the
        Y-Axis is 36 degrees per second.
 
 
    
 Z  
 
   Z   - 
Static variable in class org.proteinshader.graphics. FrenetFrames 
 The z-axis is red.
   z   - 
Variable in class org.proteinshader.math. Point3d 
 The public z-coordinate of this three element point.
   z   - 
Variable in class org.proteinshader.math. Quaternion 
 The public z-component.
   z   - 
Variable in class org.proteinshader.math. Vec3d 
 The public z-coordinate of this three element vector.
   Z_AXIS   - 
Static variable in class org.proteinshader.gui.viewing. Rotation 
 Vector representing an z-axis.
 
 
 A   B   C   D   E   F   G   H   I   J   K   L   M   N   O   P   Q   R   S   T   U   V   W   X   Y   Z  

 
   
  
 
 
 
   
 
   
          Overview     
        Package   
        Class   
        Use   
          Tree     
          Deprecated     
       Index    
          Help     
   
 
 
  
 
 
 

 
  
 PREV 
 NEXT  
  
    FRAMES     
   NO FRAMES     
  
    All Classes  ');
  }
  //-->
 
 
    All Classes  
 


  
 
 
  
 

 

                  Copyright © 2007-2008
